# Two Phosphodiesterase Genes, *PDEL* and *PDEH*, Regulate Development and Pathogenicity by Modulating Intracellular Cyclic AMP Levels in *Magnaporthe oryzae*

**DOI:** 10.1371/journal.pone.0017241

**Published:** 2011-02-28

**Authors:** Haifeng Zhang, Kaiyue Liu, Xing Zhang, Wei Tang, Jiansheng Wang, Min Guo, Qian Zhao, Xiaobo Zheng, Ping Wang, Zhengguang Zhang

**Affiliations:** 1 Department of Plant Pathology, College of Plant Protection, Nanjing Agricultural University, and Key Laboratory of Monitoring and Management of Crop Diseases and Pest Insects, Ministry of Agriculture, Nanjing, China; 2 Department of Pediatrics and the Research Institute for Children, Louisiana State University Health Sciences Center, New Orleans, Louisiana, United States of America; University of Medicine & Dentistry of New Jersey - New Jersey Medical School, United States of America

## Abstract

Cyclic AMP (cAMP) signaling plays an important role in regulating multiple cellular responses, such as growth, morphogenesis, and/or pathogenicity of eukaryotic organisms such as fungi. As a second messenger, cAMP is important in the activation of downstream effector molecules. The balance of intracellular cAMP levels depends on biosynthesis by adenylyl cyclases (ACs) and hydrolysis by cAMP phosphodiesterases (PDEases). The rice blast fungus *Magnaporthe oryzae* contains a high-affinity (PdeH/Pde2) and a low-affinity (PdeL/Pde1) PDEases, and a previous study showed that PdeH has a major role in asexual differentiation and pathogenicity. Here, we show that PdeL is required for asexual development and conidial morphology, and it also plays a minor role in regulating cAMP signaling. This is in contrast to PdeH whose mutation resulted in major defects in conidial morphology, cell wall integrity, and surface hydrophobicity, as well as a significant reduction in pathogenicity. Consistent with both PdeH and PdeL functioning in cAMP signaling, disruption of *PDEH* only partially rescued the mutant phenotype of *ΔmagB* and *Δpka1*. Further studies suggest that PdeH might function through a feedback mechanism to regulate the expression of pathogenicity factor Mpg1 during surface hydrophobicity and pathogenic development. Moreover, microarray data revealed new insights into the underlying cAMP regulatory mechanisms that may help to identify potential pathogenicity factors for the development of new disease management strategies.

## Introduction

Heterotrimeric G protein signaling is one of the most important mechanisms by which eukaryotic cells sense extracellular signals and integrate them into intrinsic signal transduction pathways, such as cAMP-dependent signaling pathway. cAMP is a ubiquitous second messenger produced in cells in response to hormones and nutrients [1]. The level of cAMP is dependent on the actions of many different proteins that affect its synthesis and degradation, such as PDEases. As a second messenger, cAMP plays an important role in activating downstream signaling components, such as phosphorylating enzyme protein kinase A (PKA). Both cAMP and PKA play key roles in the phosphorylation and regulation of enzyme substrates involved in intermediary metabolism [1].

In fungi, the highly conserved components of the cAMP-signaling cascade have been co-opted for a variety of purposes. In *Saccharomyces cerevisiae*, intracellular cAMP levels are regulated by the activity of the low- and high-affinity PDEases, Pde1 and Pde2, respectively. Pde2 is a high-affinity PDEase expressed in many organisms ranging from fungi to mammals [2]. In contrast, the low-affinity PDEase Pde1 is less well characterized but has been found in a wide range of organisms, including *S. cerevisiae*, *Schizosaccharomyces pombe*, *Candida albicans*, *Dictyostelium discoideum*, *Vibrio fischeri*, *Leishmania mexicana*, *Trypanosoma brucei*, and *Trypanosoma cruzi* [3], [4], [5], [6], [7], [8], [9], [10].

In *S. cerevisiae*, Pde2 modulates the basal level of cAMP, which is important in regulating nutrient sensing, pseudohyphal differentiation, cell cycle progression, and stress signaling [11], [12], [13], [14], [15], [16]. In contrast, Pde1 does not significantly affect basal levels of cAMP and does not have any obvious mutant phenotype. In *S. pombe*, mating, sporulation, and gluconeogenesis are regulated by cAMP/low-affinity Pde1 [17], [18], [4], [19]. In both *S. cerevisiae* and *S*. *pombe*, Pde1 regulates cAMP levels in responding to the presence of glucose [20], [21]. In *S. cerevisiae*, the cAMP degradation activity of Pde1 is positively regulated by the PKA catalytic subunits [22], whereas in *S. pombe* the regulation of Pde1 activity is also seen to be dependent on the PKA catalytic subunits or allosteric activation by cAMP [20], [23]. Pde2 was shown to control intracellular cAMP levels of human pathogenic fungus *C. albicans* and deletion of *PDE2* encoding Pde2 leads to defects in filamentation, nutrient sensing, entry into stationary phase, and cell wall and membrane integrity [24], [25]. Unlike *S. cerevisiae* and *C. albicans*, deletion of *PDE2* in the human fungal pathogen *Cryptococcus neoformans* resulted in subtle mutant phenotypes, in contrast to deletion of *PDE1*, which led to elevation of the basal intracellular cAMP levels. The *Δpde1* mutant displayed certain defects in sexual differentiation and several important characteristics of virulence, and, moreover, the Pde1 activity is regulated through PKA-derived phosphorylation [26]. Additionally, cAMP signaling is known to modulate dimorphic transition and virulence of the plant pathogenic fungus *Ustilago maydis* [27], [28], [29], [30], [31] and morphogenesis, cell polarity, and asexual development of *Neurospora crassa* and *Aspergillus nidulans* [32], [33], [34], [35], [36], [37], [38], [39], [40], [41], [42].

*Magnaporthe oryzae*, the causal agent of rice blast disease, is the most destructive pathogen of cultivated rice worldwide [43]. Several different stages of the disease cycle are essential for successful disease development. Upon contact, asexual conidia become firmly attached to the host leaves, with the aid of the mucilage stored in their tips. Subsequently, the conidia germinate and form appressoria towards the end of germ tubes. Enormous turgor pressure is generated to penetrate the plant cuticle by the appressoria [44], [45]. Initiation of appressorium formation in *M. oryzae* was shown to require cAMP signaling, because deletion of *MAC1* encoding adenylyl cyclase resulted in a defect in appressorium formation [46]. This defect could be restored by adding exogenous cAMP or by a second-site mutation in *SUM1* encoding the regulatory subunit of PKA, resulting in constitutive activation of the PKA catalytic subunit [47]. Consistent with these observations, cPKA (the catalytic subunit of PKA) replacement mutants showed delayed appressorium formation and formation of small, misshapen, and nonfunctional appressoria [48], [49]. Moreover, disruption of the *MAGB* gene encoding a Galpha subunit resulted in significant reductions in vegetative growth, conidiation, appressorium formation, and pathogenicity [50]. Conversely, expression of a dominant active MagB allele caused appressoria to form on non-inductive surfaces and addition of cAMP can restore appressorium formation in the *ΔmagB* mutant [50], [51], [52]. A regulator of G protein signaling Rgs1 was recently shown to negatively regulate G protein signaling and the cAMP pathway of *M. oryzae* [53]. Deletion of *RGS1* resulted in a significant increase in intracellular cAMP levels and formation of appressoria on hydrophylic surfaces, indicating that Rgs1 is an important negative regulator of appressorium development [53]. The low- and high-affinity PDEases, PdeL (Pde1) and PdeH (Pde2), were recently described for *M. oryzae* in an elegant study by Ramanujam and Naqvi [54]. The study demonstratied that PdeH is a key regulator of asexual and pathogenic development [54]. Here, we provide further evidence indicating that PdeL also plays a role in regulating the intracellular cAMP level, asexual development, and conidial morphology. Additionally, PdeH has a role in regulating intracellular cAMP levels during pathogenic and invasive growth, as deletion of *PDEH* resulted in defects in conidial morphology, cell wall integrity, surface hydrophobicity, and attenuated pathogenicity. Moreover, disruption of *PDEH* partially rescued mutant phenotypes of *ΔmagB* and *Δpka1*, and additional studies suggested that PdeH may function through a feedback mechanism to regulate expression of the pathogenicity factor Mpg1, which is involved in the regulation of the surface hydrophobicity and pathogenicity of *M. oryzae*.

## Materials and Methods

### Strains and culture conditions

Guy11 was used as a wild type strain in this study. All strains were cultured on complete medium (CM) agar plates [55]. Liquid CM medium was used to prepare the vegetative mycelia to extract DNA and RNA. For conidiation, strain blocks were maintained on straw decoction and corn agar media (SDC: 100 g of straw, 40 g of corn powder, 15 g of agar in 1 L of distilled water) at 28°C for 7 days in the dark followed by 3 days of continuous illumination under fluorescent light.

### Targeted gene deletion and complementation analysis

The *PDEL* and *PDEH* gene deletion mutants were generated using the standard one-step gene replacement strategy. First, two 1.0 kb of sequences flanking of targeted gene were PCR amplified with primer pairs FL656 & FL657, FL658 & FL659 (for *PDEL*) and FL660 & FL661, FL662 & FL663 (for *PDEH*) (Table S1), then a ∼2 kb fragment containing the two flanking sequences was amplified with primers FL656/FL659 (for *PDEL*) and FL660/FL663 (for *PDEH*) by overlap PCR. All amplified sequences and fragments were sequenced and ligated to flank the hygromycin resistance cassette in pMD19-T vector (Takara Co. Dalian, China). The ∼3.4-kb fragments amplified by primers FL656/FL659 (for *PDEL*) and FL660/FL663 (for *PDEH*) were transformed into protoplasts of wild type Guy11. The 3.3-kb and 4.9-kb fragments which contained the entire *PDEL* and *PDEH* genes were amplified by PCR with primers FL1033/FL1034 and FL1035/FL10346, respectively, and inserted into pCB1532 (sulphonylurea resistance) to complement the *ΔpdeL* and *ΔpdeH* strains. For double gene deletion in the *ΔpdeH* mutant, the same strategy was used and the hygromycin resistance cassette was replaced by sulphonylurea (*SUR*) resistance cassette to screen the transformants.

### Hyphal autolysis and surface hydrophobicity assays

For hyphal autolysis assay, small agar blocks were cut from the edge of 4-day-old cultures and placed onto CM medium with 1 M sorbitol and cultured in the dark at 28°C for two weeks. The size and morphology of the colonies were examined every day and photographed at the 14th day. The experiment was performed in triplicate. For surface hydrophobicity assay, the strains were plated onto CM agar plates and incubated at 28°C for 14-day. Sterile distilled water (10 ml) was placed on the surface of cultures. In addition, wettability of the aerial hyphae to solution containing both 0.02% SDS and 5 mM EDTA was also assessed as previously described [56].

### Conidiation and appressorium formation assays

For conidiation, 10-day-old conidia were collected with 3 ml of distilled water, filtered through three layers of lens paper and counted with a haemacytometer under a microscope. The conidial size was measured by a built-in microscope ruler. More than 200 conidia were measured for each strain. For appressorium formation, conidia were resuspended to a concentration of 5×10^4^ spores per milliliter in sterile water. Droplets (30 µl) of conidial suspension were placed on plastic cover slips (hydrophobic) and Gelbond films (hydrophylic) and incubated under humid conditions at room temperature as described previously [57]. Appressorium formation rate was counted at 24 hours post-inoculation (hpi) under the microscope, more than 200 appressoria were counted for each strain. Photographs were taken at 24 days post inoculation (hpi).

### Pathogenicity assay

Conidia were harvested as described above and resuspended to a concentration of 5×10^4^ spores per milliliter in a 0.2% (w/v) gelatin solution. Two-week-old seedlings of rice (*Oryza sativa* cv CO39) and 7-day-old seedlings of barley (cv Four-arris) were used for the assay. Three 20 µl droplets were placed onto the upper side of the detached barley leaves maintained on 4% (w/v) water agar plates. Pictures were taken 5 days after incubation at 25°C. For spray inoculation, 5 ml of conidial suspension of each treatment was sprayed onto rice with a sprayer. Inoculated plants were kept in a growth chamber at 25°C with 90% humidity and in the dark for the first 24 hours, followed by a 12/12 hours light/dark cycle. Lesion formation was observed daily. Photographs were taken 7 days after inoculation.

### Rice sheath penetration and turgor assay

For microscopic observation of penetration and infectious hyphae expansion on rice tissue, rice cultivar CO-39 was prepared as previously described [58] and inoculated with 100 µl of conidial suspension (1×10^4^ spores per milliliter) on the inner leaf sheath cuticle cells. After 48 hours incubation under humid conditions at room temperature, the leaf sheaths were observed under a microscope. Appressorium turgor was measured by incipient cytorrhysis (cell collapse) assay using a 1–5 M glycerol solution as described previously [44].

### Laccase activity assay

Laccase activity was monitored on 0.2 mM 2, 2′-azino-di-3-ethylbenzath- iazoline-6-sulfonate (ABTS) CM agar plate assays using mycelial blocks at 2 dpi in dark at 28°C. The enzyme activity was measured from 2-day-old CM liquid cultures. Mycelia were removed completely by filtration and centrifugation (5,000 g at 4°C) and processed using a colorimetric determination as described previously [59].

### Intracellular cAMP measurement

Two-day-old liquid mycelial cultures were harvested, frozen in liquid nitrogen and lyophilized for 16 hours. Intracellular cAMP extraction was followed as previously described [53]. The cAMP levels were quantified according to the cAMP Biotrak Immuno-assay System (Amersham Biosciences, NJ, USA).

### Construction of *MPG1* over-expression construct

To generate *MPG1* over-expression construct, the genomic DNA of Guy11 was amplified by PCR with primers FL3972/FL3973. The resulting PCR product contained *MPG1* gene sequence driven by TrpC promoter. It was then digested with *Hind*III and *Kpn*I and cloned into pCB1532, resulting in the *MPG1* expression vector pCB1532::*MPG1*. After transforming the pCB1532::*MPG1* into *ΔpdeH* mutant, the *SUR* resistant transformants were screened by qRT-PCR with primers MPG1_QF/MPG1_QR. The *ΔpdeH MPG1-3* was one of the transformants with the high *MPG1* expression level. The *ΔpdeH MPG1-2* with the low *MPG1* expression level than *ΔpdeH* mutant was used as a control.

### Nucleic acid manipulation and DNA microarray

The standard Southern blot protocol was utilized. The target gene probe and *HPH* probe were amplified with primer pairs FL467/FL2180 (for *PDEL*), FL468/FL2181 (for *PDEH*) and FL1111/FL1112 (for *HPH*), respectively. Probe labeling, hybridization and detection were performed with the DIG High Prime DNA Labeling and Detection Starter Kit I. RNA was isolated from frozen fungal mycelia, conidia (10-day old), appressoria (24 hpi) and infected rice leaves (72 hpi) with TRIzol Reagent (Invitrogen, USA). Quantitative Real-time PCR (qRT-PCR) was run on the Applied Biosystems 7300 Real Time PCR System with SYBR *Premix Ex Taq™* (Perfect Real Time, Takara, Japan). Normalization and comparison of mean Ct values were performed as described [60]. The experiment was conducted twice with three independent biological replicates.

For microarray, RNA was purified by QIAGEN RNAeasy mini kit (Qiagen Inc., Valencia, CA, USA) and quality analysis and quantification performed using the Agilent Bioanalyzer (Agilent Technologies, Inc., Wilmington, DE, USA) and the Nano Drop (NanoDrop Wilmington, DE, USA). Three biological replicates of each RNA sample were performed. Microarray data collection and correlation analysis was carried out by SBC (Shanghai Biochip Co., Ltd. China).

The above primers used in this paper were listed in Table S1.

### Gene Ontology and functional annotation

Orthologs were identified between *M. oryzae* predicted proteins and proteins in the GO database [61] via searching reciprocal best hits with the following cut-offs; e-value, 1.0e^-3^, and identity, 20%. Results from local alignment using BLAST, functional domain comparisons from NCBI and prediction of signal peptides from SignalP 3.0 software and a manual literature review were used to make final assignments to GO functional categories.

## Results

### *PDEH* is highly expressed during plant colonization but not in mycelial growth

To gain insight into functions of PdeL and PdeH, we first examined the gene expression profiles at different stages of fungal development by qRT-PCR. There was no significant difference in the abundance of *PDEL* transcripts. However, higher levels of *PDEH* expression were found in conidia, appressoria, and infection stages than in mycelia. In the infection stage, the expression of *PDEH* increased by almost 23-fold (Table 1). These observations suggested that PdeH might have an important role in *in planta* infection.

**Table 1:** Real-time RT-PCR quantification of PdeL and PdeH expression in *M. oryzae*.

*PdeL*			
RNA (Wild-type)	*PDEL* CT^a^	Actin CT	Normalized *PDEL* level relative to Actin^b^
Mycelium	30.24±0.07	24.13±0.09	1.00 (0.92–1.08)^c^
Conidium	30.37±0.17	24.15±0.06	0.93 (0.82–1.07)
Appressorium	31.41±0.17	25.54±0.02	1.19 (1.04–1.36)
Infection stage	35.41±0.41	29.22±0.14	0.95 (0.71–1.28)

*PdeH*			
RNA (Wild-type)	*PDEH* CT^a^	Actin CT	Normalized *PDEH* level relative to Actin^b^
Mycelium	32.78±0.14	24.13±0.09	1.00 (0.89–1.12)^c^
Conidium	30.86±0.14	24.15±0.06	3.86 (3.45–4.32)
Appressorium	32.18±0.10	25.54±0.02	4.06 (3.70–4.44)
Infection stage	33.38±0.06	29.22±0.14	22.68 (21.11–24.37)

a Cycle number at which the fluorescence crossed the threshold. Mean and standard deviation were calculated with data from three replicates.

b Relative quantity of PDE at different developmental stages of the wild-type strain Guy11.

c The mean and range of three replicates.

### Targeted *PDEL* and *PDEH* gene replacement

To examine the roles of PdeL and PdeH, *PDEL* and *PDEH* gene replacement constructs (Figure S1A) were generated (see Materials and Methods) and transformed into the protoplasts of the wild-type strain Guy11. The resulting hygromycin-resistant transformants were screened by PCR and confirmed by Southern blotting and RT-PCR analysis. No fragment was detected in the *ΔpdeL* or *ΔpdeH* mutants when hybridizing with gene-specific probes (probe1). RT-PCR analysis indicated that there was no *PDEL* or *PDEH* transcript in the respective mutants (Figure S1B). The *ΔpdeLΔpdeH* mutant, as well as *ΔpdeHΔmagB* and *ΔpdeHΔpka1* double mutants, was also confirmed by Southern blotting and RT-PCR analysis (Figure S1B).

### PdeL but not PdeH is indispensable for conidiogenesis

To examine functions of PdeL and PdeH, conidiation of Guy11, *ΔpdeL*, *ΔpdeH* mutants, and complemented mutant strains were examined. Conidiation in 10-day-old cultures of the *ΔpdeL* mutant was markedly reduced to approximately 8% of the wild-type strain, while conidia in the *ΔpdeH* mutant were only reduced approximately 80% (Table 2). In contrast, the wild-type strain and complement strains showed normal sporulation under the same conditions. The data suggested that PdeL is required for conidiation in *M. oryzae*.

**Table 2:** Comparison of mycological characteristics among strains.

	Mycelial growth^a^	Biomass^b^	Conidiation^c^	Appressorium formation^d^ %	
Strain	(cm)	(mg)	(x100/cm^2^)	Hydrophobic	Hydrophylic
Guy11	5.10±0.15	0.0721±0.0018	103.8±26.0	97.5±5.5	0
Δ*pdeL*	5.20±0.21	0.1025±0.0025	8.8±6.5	98.8±4.2	0
Δ*pdeH*	5.05±0.18	0.0684±0.0015	82.4±29.7	99.2±3.8	88.7±3.8
Δ*pdeL*Δ*pdeH*	4.50±0.05	0.0405±0.0010	-	-	-
Δ*pdeH*Δ*pka1*	5.15±0.32	0.0655±0.0015	8.0±4.5	45.6±1.6	3.4±0.2
Δ*pdeH*Δ*magB*	5.20±0.25	0.0772±0.0023	7.0±5.3	70.3±2.7	10.2±2.7
Δ*pdeL*/*PDEL*	5.15±0.20	0.0762±0.0020	100.3±19.5	99.0±4.5	0
Δ*pdeH*/*PDEH*	5.10±0.04	0.0738±0.0019	102.8±21.0	99.2±2.7	3.2±0.7

a Diameter of hyphal radii at day 10 after incubation on complete medium agar plates at room temperature.

b Dry weight of hyphal at day 2 after incubation in liquid complete medium at room temperature by shaken at 150 rpm.

c Number of conidia harvested from a 9 cm SDC plate at day 10 after incubation at room temperature.

### PdeL and PdeH are involved in conidial morphogenesis but dispensable for mycelial growth

**Figure 1:**
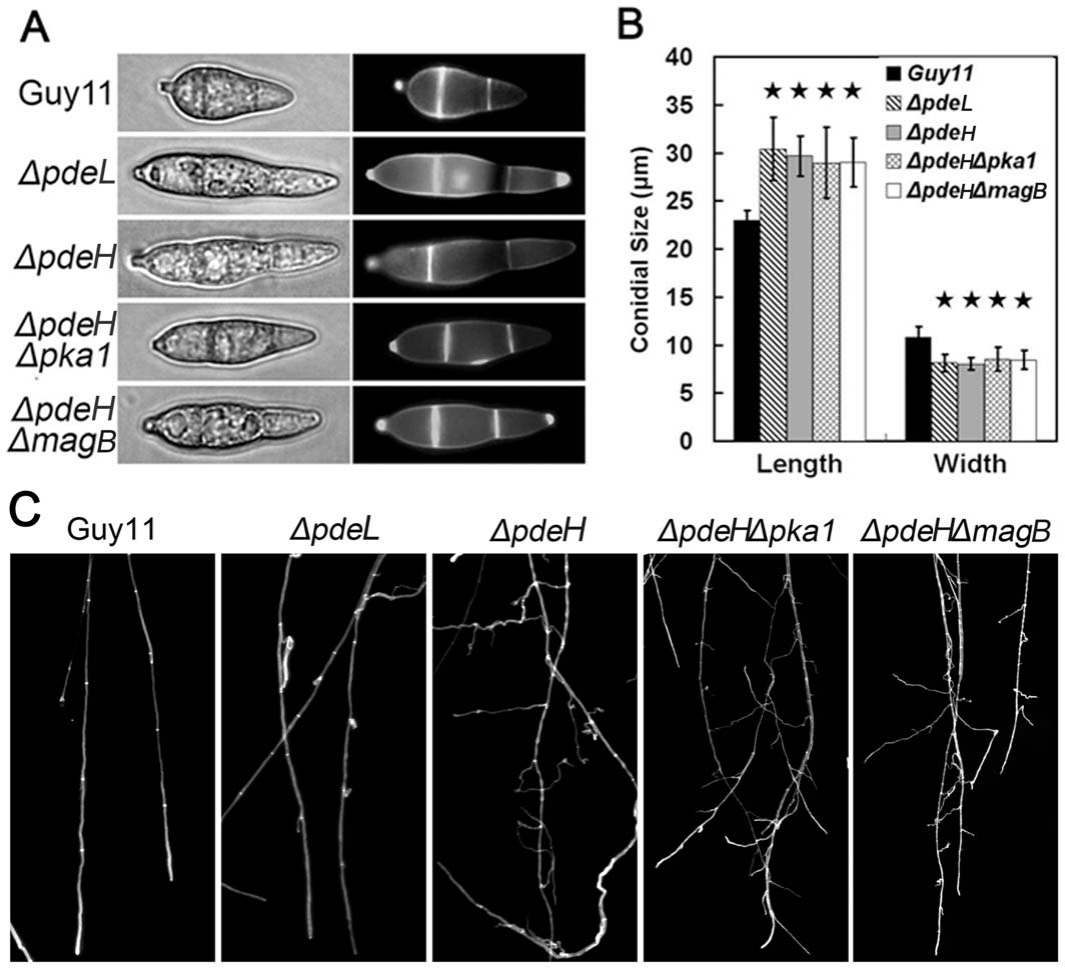
The *ΔpdeL* and *ΔpdeH* mutants have defects in conidial morphology and hyphal branching. (A) Conidia of the wild type and mutants were observed under an epi-fluorescence microscope. Conidia were stained with 1 µg/ml Calcofluor white (CFW) for 5 min in dark. (B) Conidial size of the wild type and mutants. Values are the mean ±SD from 100 conidia for each strain, which were measured using a microscope ruler. Length is the distance from the base to apex of conidia. And width is the size of the longest septum. (C) Branching patterns of mycelia on complete media slides at day 2 after incubation. Frequent branching occurs at the terminal mycelia of *ΔpdeH* and *ΔpdeHΔpdeL* double mutants. Calcofluor staining of mycelia is used to show the distance of septa.

To further explore the role of PdeL in conidiation, conidia of the *ΔpdeL* mutant was examined, along with and the *ΔpdeH* mutant and other control strains. Surprisingly, both *ΔpdeL* and *pdeH* mutants produced elongated and thinner conidia, which were uniform and readily detected under the microscope (Figure 1A). The conidia of the mutants were, on average, longer by ∼7 µm and thinner by ∼4 µm than those of the controls (Figure 1B). Frequent branching and curly tips were also observed at the terminal hyphae of the *ΔpdeH* deletion mutant. However, Calcofluor white (CFW) staining of mycelial cell walls showed that the septa were normal except for shorter intervals (Figure 1C). To determine whether PdeL and PdeH are involved in vegetative growth, the mutant and control strains were cultured on a variety of media including CM, OMA, SDC, or V8 juice agar media. No significant difference in colony morphology or growth rate was observed (Table 2). Combined, these findings suggested that PdeL and PdeH are indispensable for conidium morphology and but dispensable for mycelial growth.

### PdeH is essential for maintenance of cell wall integrity

**Figure 2:**
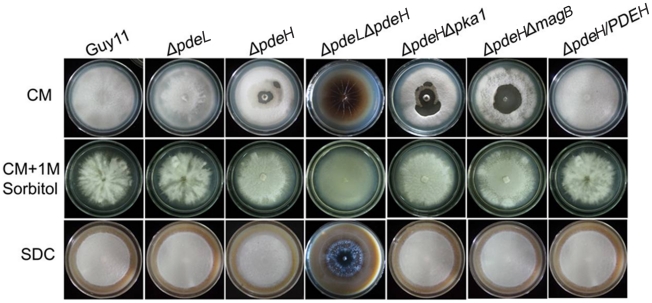
*ΔpdeH* mutants have a defect in cell-wall integrity. Growth of wild type and mutant strains on complete media (CM) without 1 M sorbitol (top); growth of strains on CM with 1 M sorbitol (middle); growth of strains on straw decoction and corn media (SDC) without sorbitol (bottom). The *ΔpdeH* and *ΔpdeH ΔpdeL* mutants undergo progressive autolysis on CM in the absence of osmotic stabilization. Radial growth rates are identical to those of the wild-type strains.

While the growth of the *ΔpdeH* mutant appeared normal on CM agar plates (Table 2), the *ΔpdeH* mutant did undergo progressive autolysis of mycelia after prolonged incubation for at least 14 days (Figure 2, top panel). Autolysis began at the center of the colony and radiated outward. The autolysis of the *ΔpdeH* mutant can be suppressed by addition of 1 M sorbitol to the culture medium (Figure 2, middle panel). The autolysis tended to be more severe in the *ΔpdeLΔpdeH* mutant than *ΔpdeH*, similar to the *Δmps1* and Δ*mck1* mutants that exhibited a defect in cell wall integrity [62], [63]. Interestingly, the *ΔpdeH* mutant did not undergo autolysis on DSC medium under the same conditions (Figure 2, bottom panel), indicating that PdeH may also be involved in sensing nutrients in the maintenance of cell wall integrity.

### PdeH is required for surface hydrophobicity

**Figure 3:**
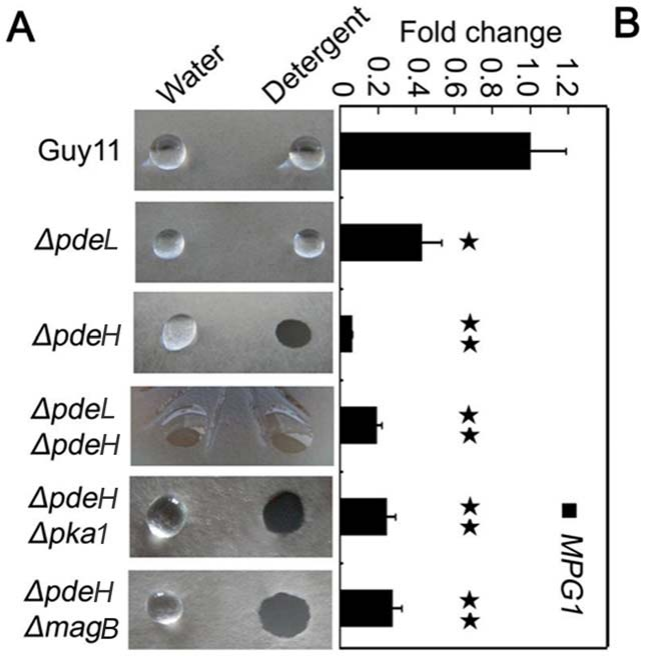
Detergent wettable phenotype of *ΔpdeH* and *ΔpdeHΔpdeL* mutants. (A) Ten microliters of water or detergent solution containing 0.02% SDS and 5 mM EDTA were placed on the colony surfaces of the wild type and mutants strains and photographed after 5 min. (B) Expression analysis of *MPG1* gene in wild type and mutant strains. The error bars indicate SD of three replicates. Asterisk indicates significant differences at *P* = 0.01.

In previous studies, disruption of several *M. oryzae* hydrophobin genes, including *MPG1* and *MHP1*, resulted in a water- or detergent-soaked easily wettable phenotype [64], [65], [66], [55], [67], [68], [69]. To determine whether PdeL and PdeH are involved in surface hydrophobicity, the mutant and wild type strains were tested with water and detergent solutions. Unlike *Δmpg1*, the *ΔpdeH* mutant did not show an easily wettable phenotype when incubated with water droplets (10 µl) for 24 hours. However, aerial hyphae of *ΔpdeH* mutants that were grown on CM agar were more readily wettable by a solution containing both 0.02% SDS and 5 mM EDTA within 5 min (Figure 3A), as seen in the *Δmhp1* mutant. The detergent-wettable phenotype shown by *ΔpdeH* mutant was stably maintained up to four successive generations, suggesting that this phenotype is mitotically stable (data not shown). Based on the results described above, we suggested that the surface hydrophobicity defect of *ΔpdeH* and *ΔpdeLΔpdeH* mutants might be related to Mpg1 and Mhp1. To assess this, we examined the levels of *MPG1* and *MHP1* expression in the mutant and wild-type strains. The *MPG1* expression level showed a 50% decrease in the *ΔpdeL* mutant compared with the wild-type control, while the expression reduced more than 90% in the *ΔpdeH* and *ΔpdeLΔpdeH* mutants (Figure 3B). In contrast, there was no apparent difference in *MHP1* expression in any of the mutants compared with the wild-type control (data not shown). These results indicated that the surface hydrophobicity defects of *ΔpdeH* and *ΔpdeLΔpdeH* mutants were likely due to the lowed *MPG1* expression.

### Over-expression of *MPG1* partially restores the surface hydrophobicity and pathogenicity to the *ΔpdeH* mutant

**Figure 4:**
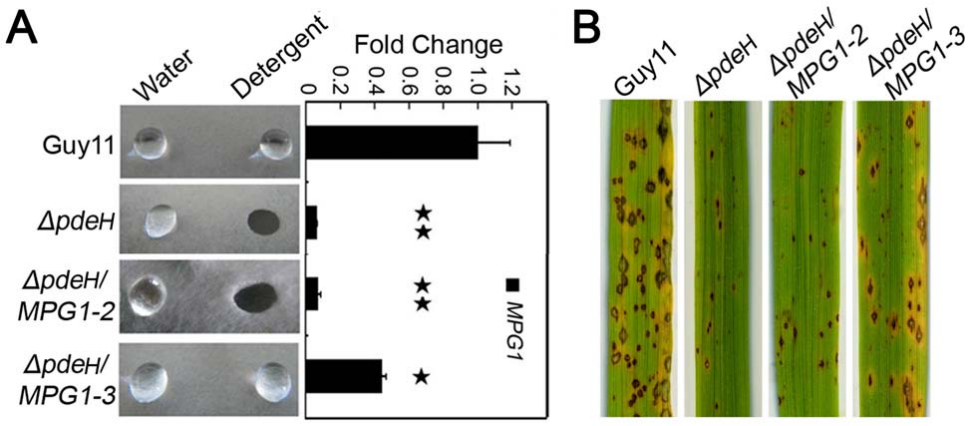
Over-expression *MPG1* in the *ΔpdeH* mutant partially restores the surface hydrophobicity and pathogenicity defects. (A) Ten microliters of water or detergent solution containing 0.02% SDS and 5 mM EDTA were placed on the colony surfaces of wild type, mutants and Mpg1 over-expression strains and photographed after 5 min. Expression analysis of *MPG1* gene in wild type and mutants and *MPG1* over-expression strains. The error bars indicate SD of three replicates. Asterisk indicates significant differences at *P* = 0.01. (B) Spraying assay. Five milliliters of conidial suspension (5×10^4^ spores/ml) of each strain were sprayed on rice seedlings. Diseased leaves were photographed 7 days after inoculation.

As mentioned above, the expression level of *MPG1* was significantly decreased in the *ΔpdeH* mutant. Additionally, Mpg1 is known as an important pathogenicity factor in *M. oryzae* [55]. We over-expressed *MPG1* in the *ΔpdeH* mutant to determine whether Mpg1 could restore the defects in surface hydrophobicity and pathogenicity. We screened the *ΔpdeH/MPG1* transformants by qRT-PCR to check the *MPG1* expression level and obtained four *MPG1*-over-expressing transformant strains. One of these, *ΔpdeH/MPG1-3*, in which the *MPG1* expression level was increased to 150% of that in the wild-type, was able to restore the defects of surface hydrophobicity and pathogenicity of the *ΔpdeH* mutant. However, the transformant *ΔpdeH/MPG1-2*, in which *MPG1* expression level was almost the same as that of the *ΔpdeH* mutant, could not complement the defects (Figure 4A and 4B). We concluded that the hydrophobicity and pathogenicity defects in the *ΔpdeH* mutant were primarily due to the low level of *MPG1* expression, and it is likely that the expression level of *MPG1* must remain at 150% of wild-type or more to maintain the surface hydrophobicity.

### PdeL and PdeH regulate intracellular cAMP levels

**Figure 5:**
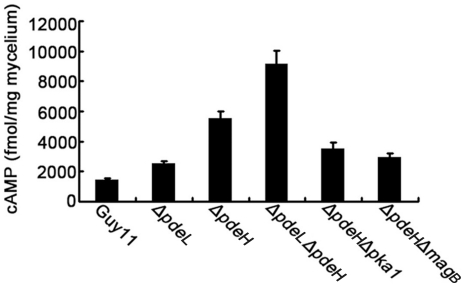
PdeL and PdeH regulate intracellular cAMP levels during pathogenesis. Loss of *PDEL* and *PDEH* leads to increased accumulation of cAMP levels. Bar chart showing quantification of intracellular cAMPs in the mycelia of the indicated strains cultured for 2 days in complete medium. Two biological repetitions with three replicates were assayed. The error bars represent SD of three replicates.

Abundant studies have shown that PDEases can regulate intracellular cAMP levels in various organisms [3], [4], [5], [6], [7], [8], [9], [10]. To determine whether PdeL and PdeH also regulate cAMP levels in *M. oryzae*, we measured the intracellular cAMP levels in the hyphal stage. The results indicated that the *ΔpdeL* mutant accumulated only ∼1.5-fold higher levels of cAMP than the wild-type strain, while the *ΔpdeH* mutant and the *ΔpdeL ΔpdeH* double mutant accumulated ∼3-fold and ∼4.5-fold higher levels cAMP compared with the wild-type, respectively (Figure 5).

### PdeH is required for appressorium differentiation, full virulence and elicitation of plant defense responses

**Figure 6:**
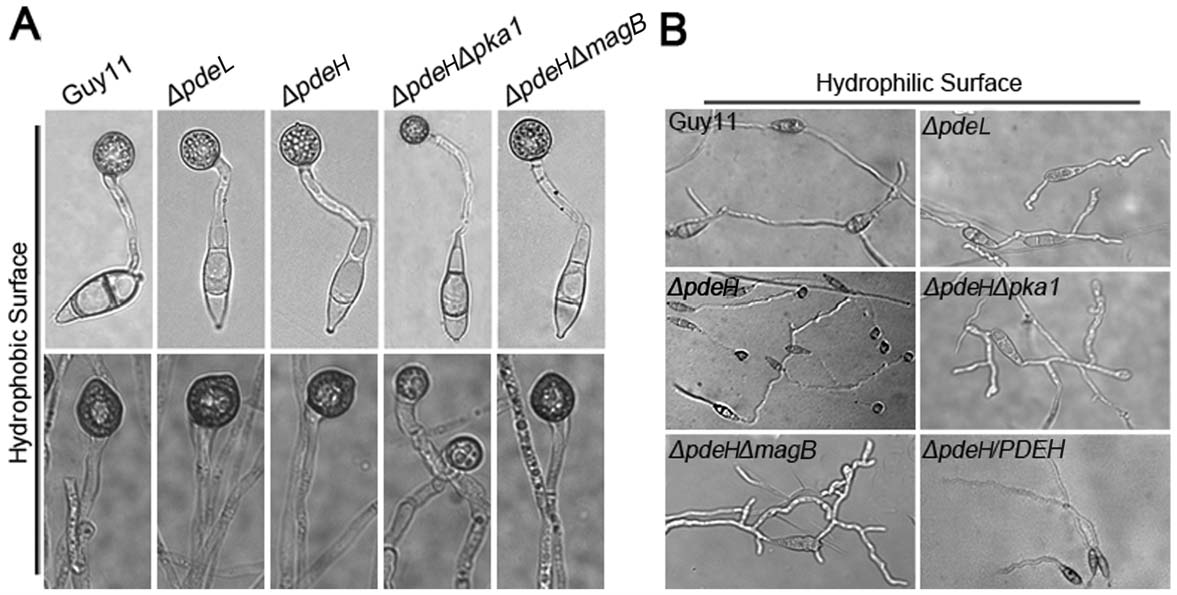
Appressorium formation assays. (A) Conidia of each strain were incubated on hydrophobic surfaces for 24 hours (up panel); hyphal plugs of each strain were incubated on hydrophobic surfaces for 48 hours (bottom panel). (B) Conidia of each strain were incubated on hydrophilic surfaces for 24 hours.

Physical cues of inductive surfaces, such as hardness and hydrophobicity, are necessary for appressorium formation [53]. The wild-type strain is unable to form appressoria on non-inductive surfaces, except in the presence of exogenous cAMP or inhibitors of PDEases [70]. To verify the effects of increased cAMP levels in the *ΔpdeL* and *ΔpdeH* mutants, we examined appressorium formation on inductive and non-inductive surfaces. The conidia and hyphal tips of the *ΔpdeL* and *ΔpdeH* mutants formed normal melanized appressoria on inductive surfaces, similar to the wild-type strain (Figure 6A, Table 2). However, the *ΔpdeH* mutant was able to form normal melanized appressoria on non-inductive surfaces (Figure 6B, Table 2), indicating that PdeH is an important negative regulator of appressorium formation.

**Figure 7:**
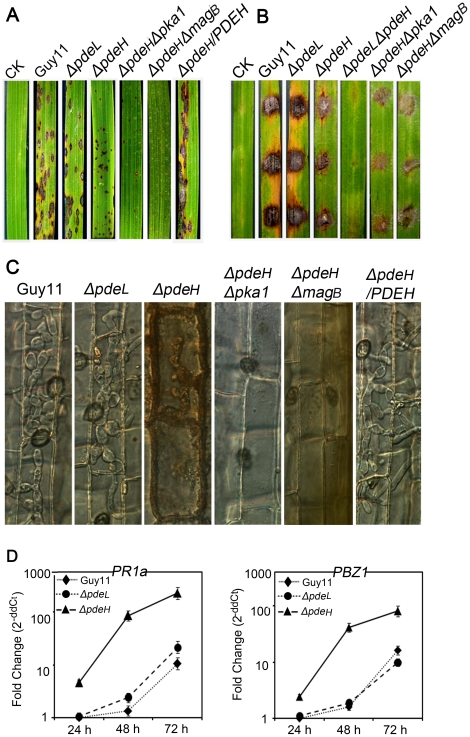
The loss of *PDEH* leads to reduced pathogenicity and induction of strong plant defense responses. (A) Spraying assay. Five milliliters of conidial suspension (5×10^4^ spores/ml) of each strain were sprayed on rice seedlings. Diseased leaves were photographed 7 days after inoculation. (B) Detached leaf assay. The hyphal plugs of each strain were placed onto the upside of detached rice seedling leaves. Diseased leaves were photographed 5 days after inoculation. (C) Observation of infectious growth. Excised rice sheath from 4-week-old rice seedlings was inoculated with conidial suspension (5×10^4^ spores/ml of each strain). Infectious growth was observed 48 hours after inoculation. (D) The expression of rice pathogenesis-related (PR) genes over time after inoculation. The transcriptional expression of *PR1a* and *PBZ1* in the infected rice was analyzed using quantitative RT-PCR. The graph was generated with three replicates in a representative data set, and similar results were obtained in another independent biological repetition. The error bars indicate SD of three replicates.

To determine whether *PDEL* and *PDEH* are involved in pathogenesis, conidial suspensions were sprayed onto susceptible rice seedlings and hyphal plugs were placed onto detached rice seedling leaves (CO-39 cv. *oryzae*) to develop rice blast lesions. In a spray-inoculation test, the *ΔpdeH* mutant produced tiny and restricted lesions, whereas the *ΔpdeL* mutant, wild-type, and complement strains showed susceptible-type spreading lesions (Figure 7A). In a detached-inoculation test, lesions caused by *ΔpdeH* mutant hyphal plugs spread very slowly and the lesion sizes were small, while those caused by *ΔpdeL* mutant, wild type, and complement strains spread rapidly and the neighboring lesions joined together by 5 days after inoculation (Figure 7B).

Because no defects in appressorium development by the *ΔpdeH* mutant were observed on hydrophobic surface, the development of infectious hyphae within the host cells was examined using excised leaf sheaths. In the wild-type, *ΔpdeL* mutant, and complement strains, infectious hyphae grew actively and extended into 3–4 cells neighboring the primary infected cells by 48 hours after inoculation (Figure 7C). However, infectious hyphae of the *ΔpdeH* mutant was mostly restricted to the primary infected cells and almost no infectious hyphae extended into neighboring cells, and accumulation of dark brown granules was seen along the infectious hyphae (Figure 7C). These results were consistent with those of rice infection assays. Additionally, the expression levels of the rice defense-related genes *PR1a* and *PBZ1* in response to *ΔpdeH* infection were much higher than those associated with infection by the wild-type, *ΔpdeL* mutant, and complement strains when analyzed by quantitative RT-PCR (Figure 7D). These observations indicated that the induction of plant defense responses in rice challenged by *ΔpdeH* mutant might contribute to the retardation of infectious hyphal development.

### PdeL and PdeH regulate laccase activities

**Figure 8:**
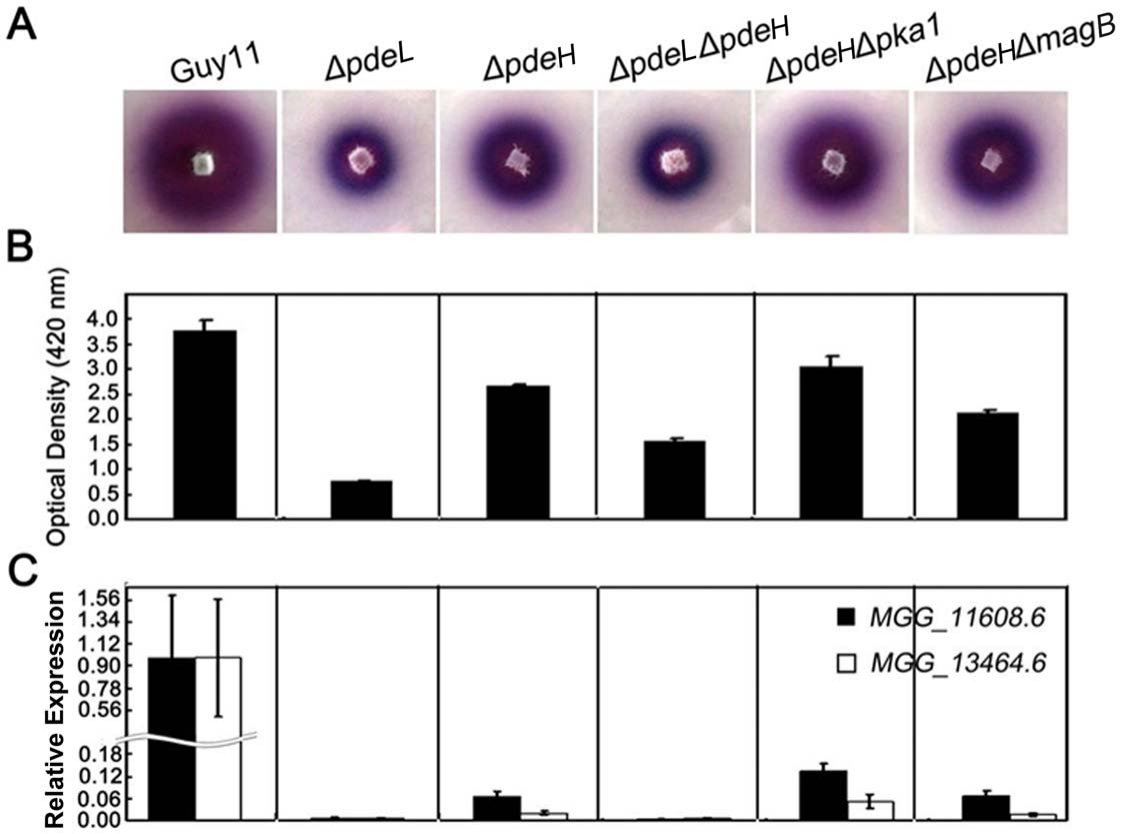
PdeL and PdeH are related to the activity of extracellular laccases. (A) Laccase activity was tested on CM agar medium containing 0.2 mM ABTS at final concentration. Discoloration was observed on day 2. (B) Laccase activity measured by ABTS oxidizing test (see Materials and Methods for details). (C) Quantitative RT-PCR analysis of two laccase genes in wild type and mutants. Expression data were normalized using the *ACTIN* gene. Error bars represent standard deviation.

A previous study indicated that laccases are involved in the pathogenicity of certain fungi [71], [97], and the laccase activity can be detected readily using the specific substrate ABTS (2,2'-azino-di-3-ethylbenzthiazoline-6-sulfonate) [72], [73]. To determine whether PDEases are involved in the regulation of laccase activity, we tested *ΔpdeL* and *ΔpdeH* mutant strains on CM agar plates and in liquid medium supplemented with 0.2 mM ABTS. In each case, decreases in laccase activity were observed in *ΔpdeL*, *ΔpdeH*, and *ΔpdeLΔpdeH* mutants, with a less-oxidized dark purple stain around the colonies of the mutants and a lower level of the laccase activity in the culture filtrate compared with the wild-type strain (Figure 8A and 8B). Consistent with these observations, the expression of two extracellular peroxidase genes, MGG_08200.6 and MGG_01924.6, was significantly down-regulated in all the mutants (Figure 8C). Meanwhile, the results also indicated that the laccase activity was decreased to a greater extent in the *ΔpdeL* mutant than in the *ΔpdeLΔpdeH* and *ΔpdeH* mutants (Figure 8A and 8B), suggesting that PdeL may have a more prominent role in regulating the enzyme activity.

### Genome-wide identification of genes regulated by PdeL and PdeH

**Figure 9:**
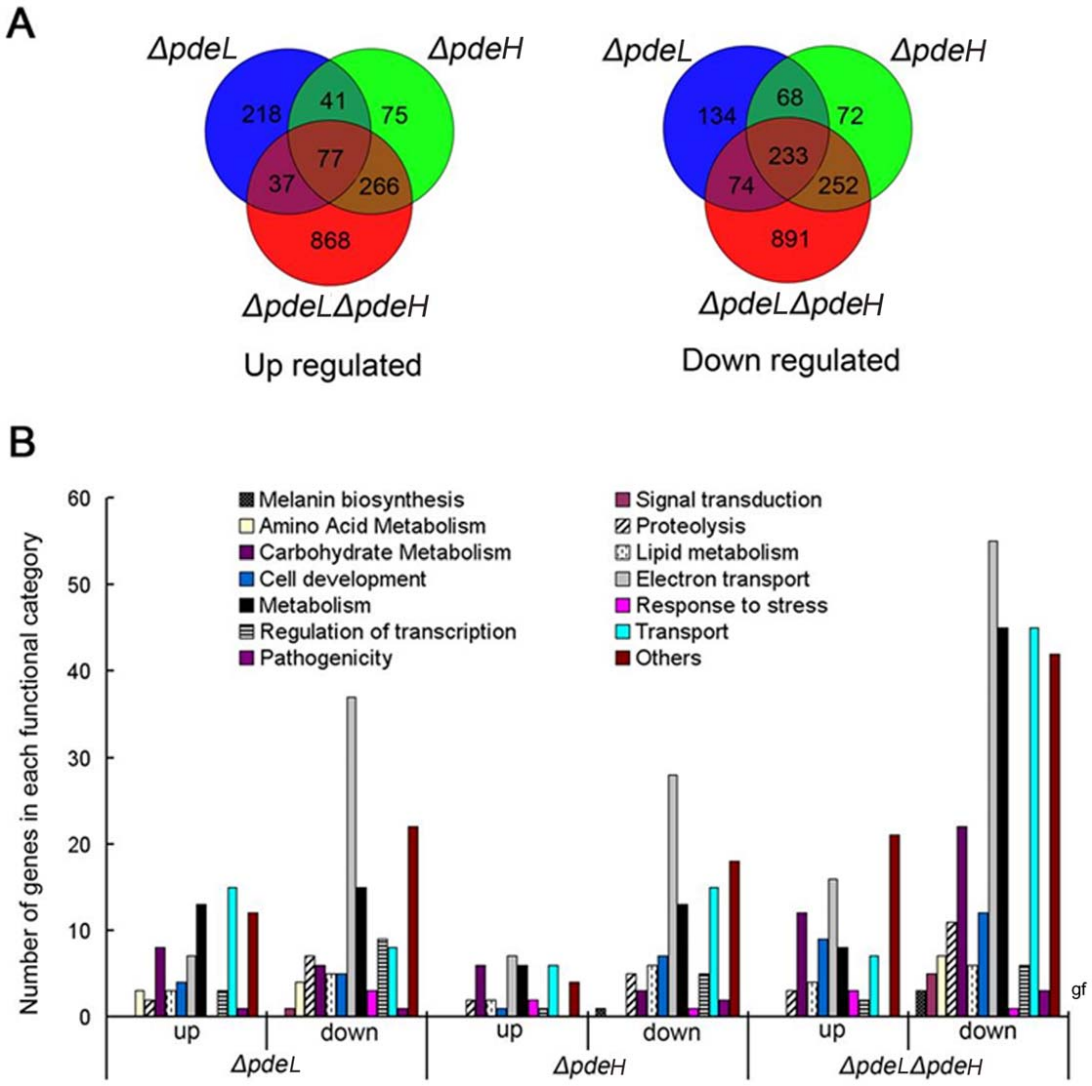
Functional categorization of the consensus genes. (A) Expression profiles were combined and showing PdeL- and PdeH-dependent. (B) Up-regulation and down-regulation of more than two-fold change genes were grouped according to their putative function (see Supplemental data for details).

To identify genes regulated by PdeL and PdeH, we compared gene expression profiles of *ΔpdeL*, *ΔpdeH*, and *ΔpdeLΔpdeH* mutants with the wild-type strain during the hyphal stage. In total, 1582 genes were up-regulated and 1724 genes were down-regulated in *ΔpdeL*, *ΔpdeH*, and *ΔpdeLΔpdeH* mutants (Figure 9A). Of the 1582 up-regulated genes, we found 373 that were regulated by PdeL, 459 by PdeH, and 1248 by PdeL and PdeH together. Of the 1724 down-regulated genes, the corresponding numbers were 509, 652, and 1450, respectively. Those genes in which the expression ratio was greater than 2-fold were functionally grouped into GO categories as described in the Materials and Methods (Figure 9B, Tables S2, S3 and S4). Overall, we noted that genes associated with protein and amino acid degradation, lipid degradation, secondary metabolism including melanin biosynthesis, and cellular transportation exhibited marked decreases in expression. Among genes that were up-regulated by PdeL, PdeH, or PdeL and PdeH, we were able to positively assign two genes, MGG_07881.6 and MGG_03508.6, to PdeH, in contrast to many by PdeL or PdeL and PdeH (Table S5).

**Figure 10:**
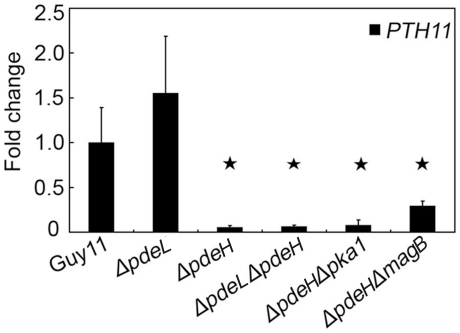
*PTH11* gene expression in *ΔpdeH* and *ΔpdeL* mutants. RNA was extracted from mycelia cultured in liquid CM medium for 2 days. *ACTIN* was used for normalization, and the values were calculated by 2^-ddCT^ methods with quantitative RT-PCR data. Values represent mean ±SD from two independent experiments with three replicates each. Asterisk indicates significant differences at *P* = 0.01.

To confirm gene expression patterns derived from our microarray experiments, we performed real-time RT-PCR with four selected genes that were down-regulated in the *ΔpdeH* mutant. Two genes, *MPG1* and *PTH11*, which are required for pathogenesis, were significantly down-regulated (∼10-fold; Figure 4B and 10). Two laccase genes, MGG_11608.6 and MGG_13464.6, were down-regulated to a greater extent (∼100-fold) in the *ΔpdeH* mutant (Figure 8C). Although the magnitudes of fold changes in laccase gene expression were much higher than the microarray results, the real-time RT-PCR data supported those of the microarray analyses.

### Characterization of novel genes regulated by PdeH

**Figure 11:**
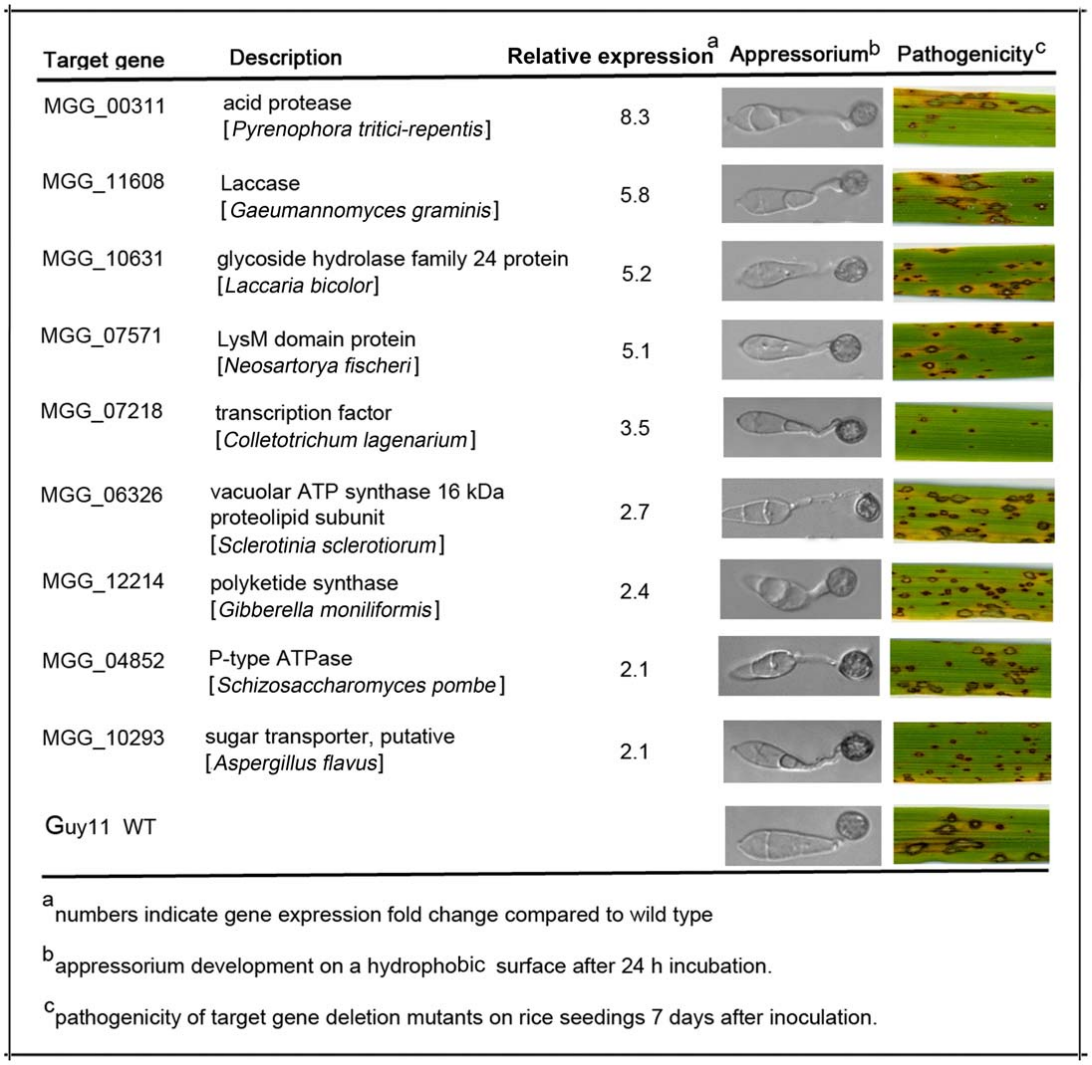
Assessment for appressorium formation and pathogenicity of PdeH targeted gene deletion mutants. Gene expression was assessed according to description in text. Appressorium formation and pathogenicity assessment were also performed as described in text.

To further explore the role of PdeH in *M. oryzae*, we deleted nine down-regulated genes identified from the *ΔpdeH* mutant microarray profile. MGG_00311.6 encodes an acid protease involved in protein and amino acid degradation, MGG_11608.6 encodes an oxidase enzyme laccase that is found in many plants, fungi, and microorganisms, MGG_10631.6 encodes a glycoside hydrolase involved in carbohydrate metabolism, MGG_07571.6 encodes a putative cell wall degrading protein with a LysM domain, MGG_07218.6 encodes a transcription factor containing a GAL4-like Zn_2_-Cys_6_-binuclear cluster DNA-binding domain at the N-terminal, MGG_06326.6 encodes a vacuolar ATP synthase 16-kDa proteolipid subunit, and, finally, MGG_12214.6encodes a polyketide synthase. Two putative ion transporters, P-type ATPase MGG_04852.6 and sugar transporter MGG_10293.6, were also characterized. Overall, mutant conidia produced normal melanized appressoria on hydrophobic surfaces, and none of them formed appressoria on noninductive surfaces (Figure 11 and data not shown). However, the mutant strain for transcription factor MGG_07218.6 showed significantly reduced pathogenicity with tiny and slow-spread blast lesions, a phenotype similar to that of the *ΔpdeH* mutant (Figure 11).

## Discussion

Cyclic AMP signaling plays an important role in regulating the growth and differentiation of eukaryotic organisms, including rice blast pathogen *M. oryzae*. A recent study by Ramanujam and Naqvi [54] has demonstrated that phosphodiesterase PdeL and PdeH have both conserved and distinct functions in regulating cAMP levels and pathogenicity of *M. oryzae*. Here, our presented our independent study that not only corroborates with that of recent publication but also further illustrates roles of PdeL and PdeH in this fungus. We found that deletion of *PDEH* resulted in defects in conidial morphology, cell wall integrity, and surface hydrophobicity, as well as a significant reduction in pathogenicity. We also found that *PDEH* disruption partially rescued the *ΔmagB* and *Δpka1* mutant phenotypes. Moreover, we propose that PdeH might function through a feedback mechanism to regulate the expression of Mpg1, which is a demonstrated pathogenicity factor involved in surface hydrophobicity and pathogenic development of *M. oryzae*.

The fungal cell wall plays an important role in maintaining cell shape and mediating exchanges between the cell and its environment [74]. Although it is rigid, its organization and structure must be remodeled constantly for growth and development [63]. Thus, in pathogenic fungi, the ability to maintain cell wall integrity is critical to the establishment of disease within the host. Several cell wall integrity-associated genes have been characterized in *S. cerevisiae*, including MEKK (Bck1), a pair of redundant MEKs (Mkk1/2), and a MAP kinase (Slt2) [75]. The Bck1 and Slt2 homologs Mck1 and Mps1 were described as also essential for cell wall integrity and pathogenicity of *M. oryzae* [76], [63]. In both *S. cerevisiae* and *C. albicans*, deletion of *PDE2* affected cell wall integrity, stress response, hyphal development, and/or virulence [25], [77], [78]. As we failed to reveal any phenotypic changes between the *ΔpdeH* mutant and the wild-type control in the response to stress agents, such as ionic stress (Na^+^ and Cu^2+^), oxidative stress (H_2_O_2_), heavy metal (CoCl_2_) stress, osmotic stress (sorbitol), or cell wall-disturbing agents (CFW, SDS, and lysing enzyme) *M. oryzae* may have an alternative approach of responding to stress signals or may possess different stress sensors.

We found that *ΔpdeLΔpdeH* double mutant undergo faster autolysis than the *ΔpdeH* mutant on CM agar plates, and the *ΔpdeLΔpdeH* mutant also showed a higher level of cAMP level than the *ΔpdeH* mutant. This suggests that cAMP plays an important role in the maintenance of cell wall integrity and, while both are involved in regulating intracellular cAMP levels and cell wall integrity, PdeH might have a more prominent role. Moreover, the *ΔpdeH* single and *ΔpdeLΔpdeH* double mutants did not undergo autolysis or showed only very slight autolysis when cultured on SDC media (poor nutrition), suggesting that PdeH may act as a nutrition sensor in modulating intracellular cAMP levels, which may affect cell wall integrity. Regardless, further studies are required to determine whether and how PdeH senses nutritional signals.

Most hydrophobins confer surface hydrophobicity on fungi forming a spore rodlet layer. Disruption of several hydrophobin genes, including Mpg1, resulted in a water- or detergent-soaked easily wettable phenotype [64], [65], [66], [55], [67], [68]. The *Mpg1* mutant is defective in conidiation and appressorium formation, and less pathogenic than wild-type strain, indicating that Mpg1 plays an important role in multiple infection-related processes. Here, deletion of *PDEH* resulted in a significantly reduced level of *MPG1* expression and defects in surface hydrophobicity and pathogenicity [55]. In *Δmpg1* mutants, the defect in appressorium formation can be restored by adding exogenous cAMP, suggesting that Mpg1 functions upstream of cAMP signal for appressorium formation [55]. Here, over-expression of *MPG1* in the *ΔpdeH* mutant partially rescued the surface hydrophobicity and pathogenicity defects, suggesting that Mpg1 could function downstream of PdeH and cAMP signaling. Thus, we propose that PdeH may function through a feedback mechanism to activate Mpg1 and regulates its expression for surface hydrophobicity and pathogenic development.

Several induced genes in plants have been reported to affect fungal pathogenicity, directly or indirectly [79], [55], [80], [81], [69]. The high level of *PDEH* expression at late infection stages in infected rice leaves also suggests its roles in infectious growth and pathogenicity. Targeted disruption of *PDEH* significantly reduced virulence in infection assays. The *ΔpdeH* mutant produced smaller and less numerous lesions than the wild-type strain. The results presented above indicated that appressoria formed by *ΔpdeH* mutants are probably defective in penetration. It is likely that PdeH regulates the processes involved in a late stage of appressorium development, such as turgor generation or appressorium pore formation. The qRT-PCR results, indicating that *PDEH* was expressed at high levels in the appressoria, also supported this possibility. We assayed appressorium turgor using the cytorrhysis method described previously [44]. Preliminary data do not indicate any difference in cytorrhysis between appressoria formed by *ΔpdeH* mutant and wild-type strains (data not shown), suggesting that appressoria of *ΔpdeH* mutants do not have defects in the generation of turgor pressure. Another possibility is that PdeH may regulate the early stages of appressorium penetration, such as development of the penetration peg or differentiation of infectious hyphae. The reduction in pathogenicity may be due to the reduction in development at the pre-penetration stages or a reduction in infectious growth of *ΔpdeH* mutants in host cells. This may also explain the high level of *PDEH* expression during the late stages of infection.

Defense responses induced by the recognition of microbe-associated molecules (pathogen-associated molecular patterns, PAMPs) are often associated with cell wall strengthening, rapid production of reactive oxygen species (ROS), and the transcriptional activation of PR genes [82]. In plants, accumulation of ROS at the site of infection is considered one of the earliest responses during plant PAMP-triggered immunity (PTI) [83], [82]. Plant-derived ROS have various functions during plant–microbe interactions [84], [85], [86], [87], [88], [89]. For pathogens to survive in harsh environments and successfully invade host cells, they must develop mechanisms to scavenge ROS and protect against ROS-induced damage [90], [91], [92], [93]. In *M. oryzae*, several well-characterized genes have been reported to suppress basal host defenses and to be responsible for ROS detoxification at the site of infection [58], [59], [94]. As rice cells infected by the *ΔpdeH* mutant showed brown granule accumulation and cell death, it is likely that plant defense responses are involved in virulence attenuation of *ΔpdeH* mutant. Thus, the defense responses against the wild-type and mutant strains were compared with regard to two genes, *PR1a* and *PBZ1*. These two genes are important components of the jasmonic acid (JA) and salicylic acid (SA) pathways, which are involved in JA- and SA-induced plant defense, respectively [95], [96]. The stronger activation of both *PR1a* and *PBZ1* in mutant-inoculated plants compared with wild-type controls suggested that *ΔpdeH* mutant can still elicit plant defenses, which may be involved in attenuation of the virulence of the *ΔpdeH* mutant.

In *M. oryzae*, many evidences suggest that MagB may sense surface cues, stimulate cAMP synthesis, and activate the cAMP signal pathway [50], [51], [52]. Because PdeH can degrade intracellular cAMP, we hypothesize that the decrease in the PdeH activity in the *ΔmagB* mutant may maintain the balance of intracellular cAMP levels and restore its defects. The result of partially restoration in appressorium formation in the *ΔpdeHΔmagB* double mutant supported this hypothesis (Table 2). Additionally, the appressorium formation rate on hydrophylic surfaces was reduced to 10% compared with *ΔpdeH* mutants, suggesting that MagB and PdeH can complement each other in some defects. However, no other defect was restored in the *ΔpdeHΔmagB* double mutant, all of which were similar to those in the *ΔpdeH* mutant (Figure 1, 2, 3 and 8). These observations may have been due to the high cAMP level in the *ΔpdeH ΔmagB* double mutant, because the cAMP level was still much higher in *ΔpdeH ΔmagB* than in the wild-type (Figure 5). Since cAMP-dependent protein kinase PKA plays a pivotal role in cAMP-dependent pathways of *M. oryzae*, we compared the phenotype of *Δpka1* with that of the *ΔpdeH Δpka1* double mutant. The *ΔpdeHΔpka1* mutant formed small, misshapen appressoria on hydrophobic surfaces similar to the *Δpka1* mutant, while very few appressoria were formed on hydropholic surfaces (Figure 6). However, other phenotypes, such as cell wall integrity and surface hydrophobicity, which may also be due to the high cAMP level, were similar to those of the *ΔpdeH* mutant (Figure 1, 2, 3 and 8). Based on these results, we conclude that, while MagB and Pka1 activate the cAMP signaling pathway to mediate appressorium formation and pathogenicity, PdeH plays a more critical role in regulating intracellular cAMP levels to affect cell wall integrity and surface hydrophobicity, bypassing the PKA signaling pathway.

Genome-wide analysis of gene expression changes during spore germination and appressorium formation on a hydrophobic surface compared with induction by cAMP revealed new insight into appressorium formation and function in *M. oryzae* [98]. During appressorium formation, the genes that respond to both stimuli are known to be involved in protein and amino acid degradation, lipid metabolism, secondary metabolism, and cellular transportation. In this study, the levels of expression of two pathogenicity-related genes were significantly decreased in the appressoria treated with cAMP. Targeted deletion of several other changed genes, such as polyketide synthase (MGG_07219.6), subtilisin-like protease (MGG_03670.6), and a transcription factor (MGG_07218.6), affected virulence and other characteristics related to pathogenicity. Similar to these results, our microarray data also provided some novel insight to identify pathogenicity factors. Three genes, *MPG1* (MGG_10315.6), *PTH11* (MGG_05871.6), and *COS1* (MGG_03977.6), which have been reported to be involved in pathogenicity [55], [99], [100], were significantly down-regulated in the *ΔpdeH* single and *ΔpdeLΔpdeH* double mutants, but not in the *ΔpdeL* mutant. Furthermore, deletion of a down-regulated transcription factor, from the *ΔpdeH* microarray data, also showed reduced pathogenicity, similar to that of the *ΔpdeH* mutant. These data may explain why *ΔpdeH* and *ΔpdeLΔpdeH* mutants attenuated virulence.

In summary, we continued the characterization of the low- and high-affinity cAMP PDEases PdeL and PdeH in *M. oryzae*. We showed that, while PdeL and PdeH share certain functions, PdeH indeedplays a more prominent role than PdeL in regulating cell wall integrity, surface hydrophobicity and pathogenicity through modulation of intracellular cAMP levels. Our findings also reveal that PdeH may function through a feedback mechanism to regulate the expression of pathogenicity factor Mpg1, which is involved in surface hydrophobicity and pathogenic development in *M. oryzae.*

## Supporting Information

Figure S1 **Generation of** *Δ* ***pdeL*** **and** *Δ* ***pdeH*** **deletion mutants.** (A) Restriction map of the *PDEL* and *PDEH* genomic region and knockout construct. Thick arrows indicate orientations of the *PDEL, PDEH* and hygromycin phosphotransferase (*hph)* genes. Thin lines below the arrows indicate the probe sequences of each gene. The restriction enzymes are *EcoR*V (EV), *EcoR*I (EI), *Xba*I (XI) and *Kpn*I (KI). (B) Southern blot and RT-PCR analyses of *ΔpdeL*, *ΔpdeH* (top) and double-gene (bottom) knockout mutants. Genomic DNA of the wild-type strain and the knockout mutants was digested with corresponding restriction enzymes. Total RNAs of the wild-type strain and the knockout mutants were isolated and the expression levels of target gene were detected using *ACTIN* as control. WT: wild type; T: transformant.
(TIF)

Figure S2 **Confirmation of target gene replacement by PCR.**
(PDF)

Table S1 **Primers used in this study.**
(DOC)

Table S2 **Categorization of** ***PDEL*** **regulated genes with known function.**
(DOC)

Table S3 **Categorization of** ***PDEH*** **regulated genes with known function.**
(DOC)

Table S4 **Categorization of** ***PDEL&PDEH*** **regulated genes with known function.**
(DOC)

Table S5 **Categorization of genes only dependent on** ***PDEL*** **,** ***PDEH*** **and** ***PDEL & PDEH*** **, respectively.**
(DOC)
