# Supplementary figures and images for: Two Phosphodiesterase Genes, PDEL and PDEH, Regulate Development and Pathogenicity by Modulating Intracellular Cyclic AMP Levels in Magnaporthe oryzae
Source: PLoS One. 2011 Feb 28;6(2):e17241. doi: 10.1371/journal.pone.0017241 (PMC3046207; doi:10.1371/journal.pone.0017241)

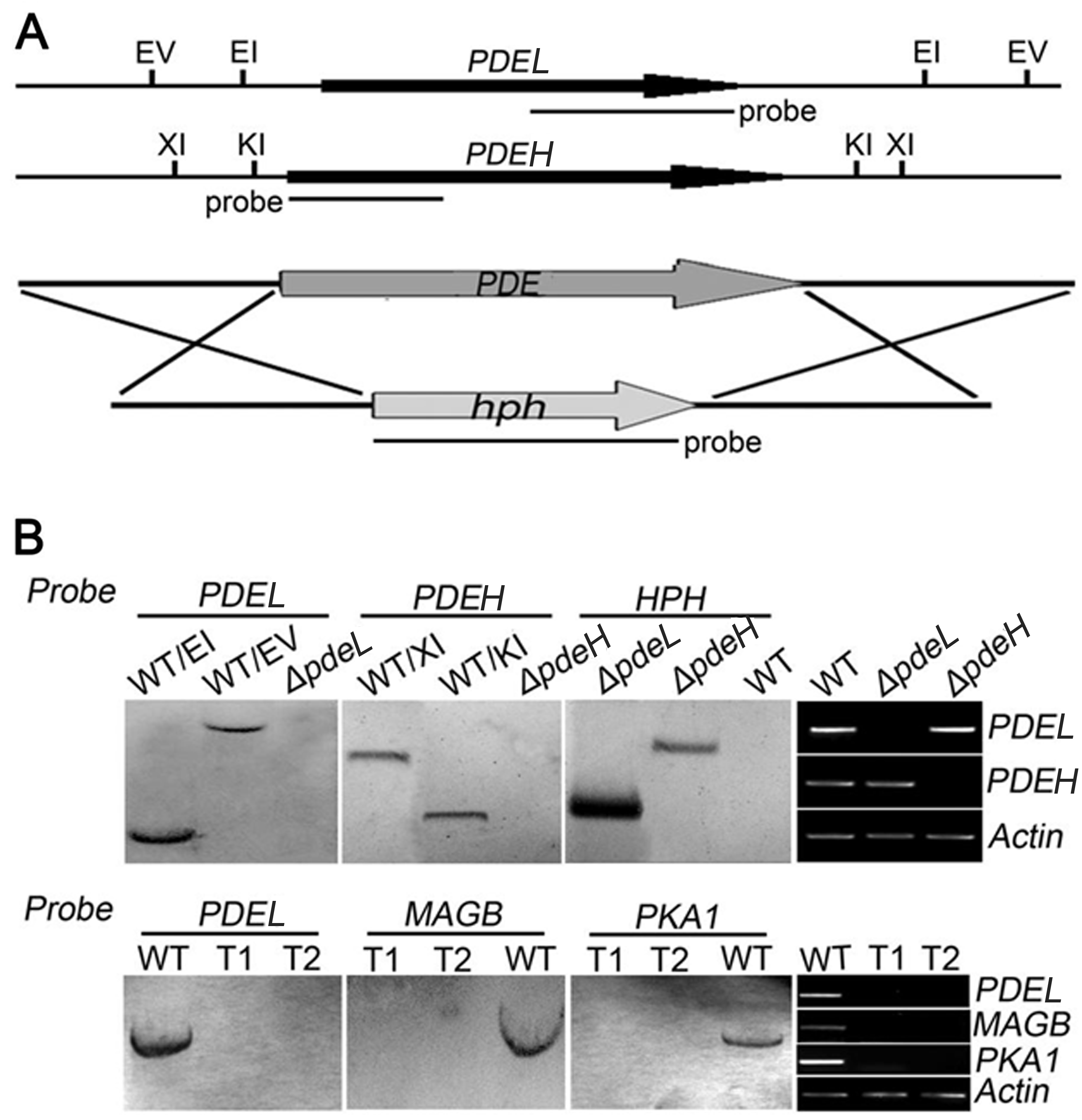

Supplement: Figure S1 — Generation of Δ pdeL and Δ pdeH deletion mutants. (A) Restriction map of the PDEL and PDEH genomic region and knockout construct. Thick arrows indicate orientations of the PDEL, PDEH and hygromycin phosphotransferase (hph) genes. Thin lines below the arrows indicate the probe sequences of each gene. The restriction enzymes are EcoRV (EV), EcoRI (EI), XbaI (XI) and KpnI (KI). (B) Southern blot and RT-PCR analyses of ΔpdeL, ΔpdeH (top) and double-gene (bottom) knockout mutants. Genomic DNA of the wild-type strain and the knockout mutants was digested with corresponding restriction enzymes. Total RNAs of the wild-type strain and the knockout mutants were isolated and the expression levels of target gene were detected using ACTIN as control. WT: wild type; T: transformant. (TIF) [file pone.0017241.s001.tif]
