# Supplementary material for: Two Phosphodiesterase Genes, PDEL and PDEH, Regulate Development and Pathogenicity by Modulating Intracellular Cyclic AMP Levels in Magnaporthe oryzae
Source: PLoS One. 2011 Feb 28;6(2):e17241. doi: 10.1371/journal.pone.0017241 (PMC3046207; doi:10.1371/journal.pone.0017241)

Confirmation of target gene replacement by PCR.

The correct replacement of target gene with hygromycin dehydrogenase gene in mutants (M1, M2) was verified by the absence of PCR products of the expected size using target gene specific primers and *ACTIN* primers. Two pairs of primers were added in the same tube. Result: Presence of the *ACTIN* gene in Guy11 (WT), ectopic (EI) and mutants (M1, M2) strains but absence of the target gene in mutants (M1, M2). The mutants (MGG_07218) which reduced virulence were further confirmed by RT-PCR.

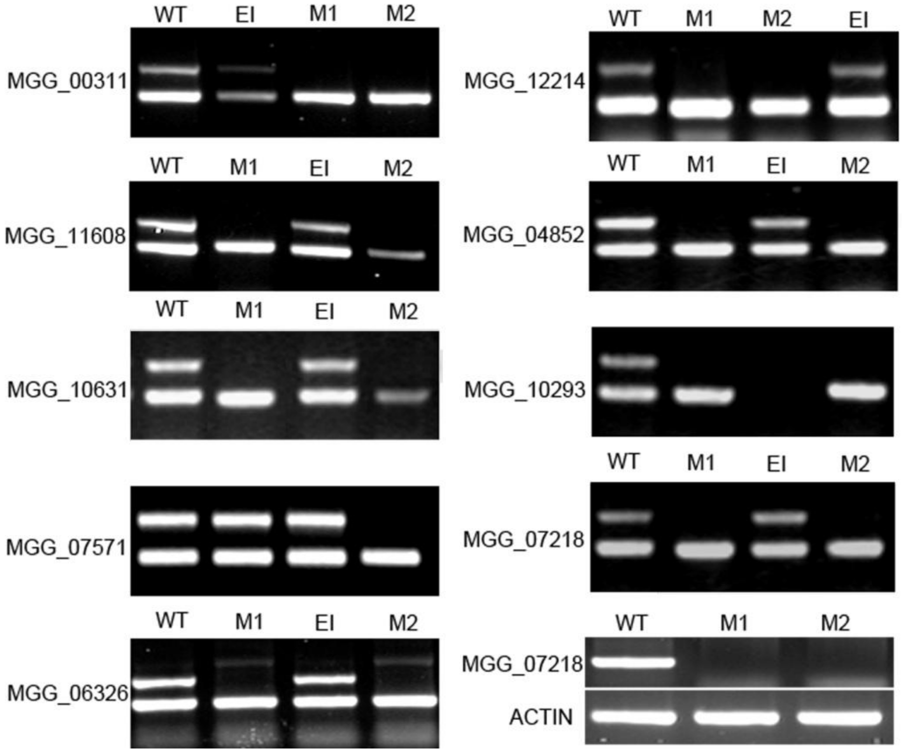

Supplement: Figure S2 — Confirmation of target gene replacement by PCR. (PDF) [file pone.0017241.s002.pdf]
