# Supplementary material for: Two Phosphodiesterase Genes, PDEL and PDEH, Regulate Development and Pathogenicity by Modulating Intracellular Cyclic AMP Levels in Magnaporthe oryzae
Source: PLoS One. 2011 Feb 28;6(2):e17241. doi: 10.1371/journal.pone.0017241 (PMC3046207; doi:10.1371/journal.pone.0017241)
Supplement: Table S1 — Primers used in this study. (DOC) [file pone.0017241.s003.doc]

**Table S1. Primers used in this study.**

| Primer | Sequence (5’-3’) |
| --- | --- |
| FL656 | cgtttgctacctgtggccaacgccg |
| FL657 | caggggactgtcatagggtcgcagatatcctcgatgtctggacaaaacgtac |
| FL658 | gtacgttttgtccagacatcgaggatatctgcgaccctatgacagtcccctg |
| FL659 | tttcgttgaggccgccaatgccacg |
| FL660 | aacgcacgaaaatctgtacacatcac |
| FL661 | ccgacaaattcttctggtctccgtttaaaccgtgggcccaaagagcgtccagt |
| FL662 | actggacgctctttgggcccacggtttaaacggagaccagaagaatttgtcgg |
| FL663 | aacagatgatccacagcgttcaatttc |
| FL1193 | gacttgttgacctggttcgagct |
| FL1194 | agcgatcgatcttgttcaggaagagatatcatgacacgcatagactgtacagtg |
| FL1195 | cactgtacagtctatgcgtgtcatgatatctcttcctgaacaagatcgatcgct |
| FL1196 | tgatccatttcgtatcccaccaag |
| FL3214 | ttcaattcgtatcgactaacg |
| FL3215 | gaccacagtcatcatcttggtgatatcacgtcgatgtgctccaccaac |
| FL3216 | gttggtggagcacatcgacgtgatatcaccaagatgatgactgtggtc |
| FL3217 | gcataagcattgtgtcccttg |
| FL467 | tcacaagtacaatgcctcgcca |
| FL2180 | tcgccatccatgaatgccatg |
| FL468 | atggagaatgctgcctgcaattt |
| FL2181 | tgggacactgagatcctaac |
| FL3705 | aggaccaaaggatggagtac |
| FL3706 | tgatgaaccacctcgagttg |
| FL3707 | cagcagcctcagatgaactc |
| FL3708 | gaccacctgagccttcttca |
| FL1033 | tatgaattcctgttcctatccatccgacaatcc |
| FL1034 | tatgtcgacgatccagcccaggattggagagat |
| FL1035 | tattctagaaggttctaccacgcaacattcgtg |
| FL1036 | tatactagtcataccaacaggacctcgtgcttc |
| FL3972 | aagcttatggctcagcagaagtgcg |
| FL3973 | ggtaccttagctcaggataggagtgc |
| ACTIN_QF | ccatgtaccctggtctttcg |
| ACTIN_QR | ttcgagatccacatctgctg |
| MPG1_QF | gagaaggtcgtctcttgctg |
| MPG1_QR | tgtccgagcagaagttgttg |
| PTH11_QF | atcaccatctgctcggacac |
| PTH11_QR | agactctcgacgacggattg |
| MGG11608_QF | cgtccactgccacatcgc |
| MGG11608_QR | agtcgtcctggtggaaagg |
| MGG13464_QF | tgttccactgccacatcg |
| MGG13464_QR | ctcagaccagagtcgtgctg |
| OsEF1α_QF | cttcaacacccctgctatg [59] |
| OsEF1α_QR | ccgttgtggtgaatgagtaa [59] |
| OsPR1a_QF | tcttcatcacctgcaactactc [59] |
| OsPR1a_QR | attcatcggatttattctcacc [59] |
| OsPBZ1_QF | ctactatggcatgctcaagat [59] |
| OsPBZ1_QR | atagaaaggcacataaacacaa [59] |
