# Supplementary material for: Two Phosphodiesterase Genes, PDEL and PDEH, Regulate Development and Pathogenicity by Modulating Intracellular Cyclic AMP Levels in Magnaporthe oryzae
Source: PLoS One. 2011 Feb 28;6(2):e17241. doi: 10.1371/journal.pone.0017241 (PMC3046207; doi:10.1371/journal.pone.0017241)
Supplement: Table S2 — Categorization of PDEL regulated genes with known function. (DOC) [file pone.0017241.s004.doc]

| **Table S2. Categorization of *PDEL* regulated genes with known function** | | | | | |
| --- | --- | --- | --- | --- | --- |
| **Category** | **Gene ID** | **Exp.** | **Signal P** | **Blast hit** | **NCBI_ID** |
| **Melanin biosynthesis (GO:0042438)** | | | | | |
| **Signal transduction (GO:0007165)** | | | | | |
|  | MGG_10544.6 | DR | N | G protein-coupled receptor GprD [Aspergillus flavus NRRL3357] | EED50805 |
| **Amino Acid Metabolism (GO:0006520)** | | | | | |
|  | MGG_05485.6 | UR | N | amine oxidase, putative [Aspergillus flavus NRRL3357] | EED48219 |
|  | MGG_05912.6 | UR | N | amidohydrolase, putative [Penicillium marneffei ATCC 18224] | XP_002149455 |
|  | MGG_00671.6 | UR | Y | arginase family protein [Aspergillus clavatus NRRL 1] | XP_001273079 |
|  | MGG_06207.6 | DR | N | leucine aminopeptidase [Aspergillus sojae] | AAN31395 |
|  | MGG_13798.6 | DR | N | putative amino acid permease [Glomus mosseae] | AAX81451 |
|  | MGG_02243.6 | DR | N | amidohydrolase family protein [Aspergillus fumigatus A1163] | EDP53371 |
|  | MGG_02295 | DR | Y | amine oxidase, flavin-containing superfamily [Penicillium marneffeiATCC 18224] | XP_002144367 |
| **Proteolysis (GO:0006508)** | | | | | |
|  | MGG_10498.6 | UR | Y | endopeptidase [Rhodococcus jostii RHA1] | YP_703158 |
|  | MGG_10459.6 | UR | Y | aspartic-type endopeptidase[Talaromyces stipitatus ATCC10500] | EED14742 |
|  | MGG_11021.6 | DR | N | AAA family ATPase, putative [Aspergillus clavatus NRRL 1] | XP_001273411 |
|  | MGG_12068.6 | DR | N | regulatory P domain-containing protein [Hahella chejuensis KCTC2396] | YP_433708 |
|  | MGG_00822.6 | DR | Y | COG3591: V8-like Glu-specific endopeptidase [Magnetospirillum magnetotacticum MS-1] | ZP_00208343 |
|  | MGG_00311.6 | DR | Y | acid protease [Pyrenophora tritici-repentis Pt-1C-BFP] | XP_001932923 |
|  | MGG_03817.6 | DR | Y | metalloprotease [Pleurotus ostreatus] | AAU94648 |
|  | MGG_00578.6 | DR | Y | protease [Streptomyces sp. Mg1] | YP_002177821 |
|  | MGG_03817.6 | DR | Y | metalloprotease [Pleurotus ostreatus] | AAU94648 |
| **Carbohydrate Metabolism (GO:0005975)** | | | | | |
|  | MGG_05364.6 | UR | N | endoglucanase IV precursor [Neurospora crassa OR74A] | XP_958254 |
|  | MGG_03287.6 | UR | N | related to alpha-amylase [Neurospora crassa] | CAE75731 |
|  | MGG_12291.6 | UR | Y | beta-hexosaminidase, putative [Aspergillus flavus NRRL3357] | EED53191 |
|  | MGG_07631.6 | UR | Y | endoglucanase [Aspergillus fumigatus Af293] | XP_755787 |
|  | MGG_07631.6 | UR | Y | endoglucanase [Aspergillus fumigatus Af293] | XP_755787 |
|  | MGG_07666.6 | UR | Y | class I alpha-mannosidase [Ophiostoma novo-ulmi] | AAG48158 |
|  | MGG_08052.6 | UR | Y | Capsular polysaccharide biosynthesis protein[Aspergillus fumigatus Af293] | XP_751886 |
|  | MGG_02562.6 | UR | Y | Glycosyltransferase sugar-binding region containingDXD motif[Pyrenophora tritici-repentis Pt-1C-BFP] | XP_001935586 |
|  | MGG_06593.6 | DR | Y | endoxylanase II; pI 9 [Hypocrea jecorina] | AAB29346 |
|  | MGG_00863.6 | DR | Y | cell wall glucanase (Scw4), putative [Penicillium marneffei ATCC18224] | XP_002147497 |
|  | MGG_09681.6 | DR | N | gluconolactonase precursor [Pyrenophora tritici-repentis Pt-1C-BFP] | XP_001942293 |
|  | MGG_09189.6 | DR | Y | choline dehydrogenase [Pyrenophora tritici-repentis Pt-1C-BFP] | XP_001937164 |
|  | MGG_14061.6 | DR | Y | oxalate decarboxylase, putative [Aspergillus flavus NRRL3357] | EED56627 |
|  | MGG_09681.6 | DR | N | gluconolactonase precursor [Pyrenophora tritici-repentis Pt-1C-BFP] | XP_001942293 |
| **Lipid metabolism (GO:0006629)** | | | | | |
|  | MGG_00806.6 | UR | N | polyketide synthase [Xylaria sp. BCC 1067] | AAY40862 |
|  | MGG_03081.6 | UR | N | Glycosyltransferase family 25 (LPS biosynthesis protein)[Penicillium chrysogenum Wisconsin 54-1255] | CAP93867 |
|  | MGG_02269.6 | UR | N | LPS glycosyltransferase [Aspergillus fumigatus Af293] | XP_747140 |
|  | MGG_08232.6 | DR | Y | LPS glycosyltransferase [Aspergillus fumigatus Af293] | XP_747140 |
|  | MGG_12214.6 | DR | N | polyketide synthase [Gibberella moniliformis] | AAR92209 |
|  | MGG_08416.6 | DR | N | triacylglycerol lipase, putative [Aspergillus flavus NRRL3357] | EED45307 |
|  | MGG_09019.6 | DR | N | secretory phospholipase A2 [Aspergillus oryzae] | BAD01582 |
|  | MGG_04194.6 | DR | Y | acetyl esterase [Hypocrea jecorina] | ABI34466 |
| **Cell development (GO:0007275)** | | | | | |
|  | MGG_04344.6 | UR | N | regulatory P domain-containing protein [Hahella chejuensis KCTC2396] | YP_433708 |
|  | MGG_13261.6 | UR | N | serine-threonine rich protein, putative [Talaromyces stipitatus ATCC 10500] | EED23712 |
|  | MGG_09806.6 | UR | Y | alpha-1,3-mannosyltransferase, putative [Aspergillus fumigatusA1163] | EDP55339 |
|  | MGG_10750.6 | UR | N | OPT oligopeptide transporter protein[Yarrowia lipolytica] | XP_501979 |
|  | MGG_08003.6 | DR | N | UbiA prenyltransferase [Chloroflexus sp. Y-400-fl] | YP_002568665 |
|  | MGG_07367.6 | DR | Y | cell surface spherulin 4-like protein, putative [Talaromyces stipitatus ATCC 10500] | EED17159 |
|  | MGG_05824 | DR | N | UDP-glucuronosyl/UDP-glucosyltransferase [Mycobacterium vanbaalenii PYR-1] | YP_955665 |
|  | MGG_10908 | DR | N | FAD-dependent monooxygenase (PaxM) [Aspergillus fumigatus Af293 | XP_751271 |
|  | MGG_05232.6 | DR | Y | IgE-binding protein [Aspergillus fumigatus Af293] | XP_731512 |
| **Electron transport (GO:0006118)** | | | | | |
|  | MGG_13262.6 | UR | Y | 6-hydroxy-D-nicotine oxidase, putative [Aspergillus flavusNRRL3357] | EED53119 |
|  | MGG_09103.6 | UR | N | L-ascorbate oxidase, putative [Aspergillus flavus NRRL3357] | EED57720 |
|  | MGG_03707.6 | UR | N | quinone oxidoreductase [Pichia stipitis CBS 6054] | XP_001387895 |
|  | MGG_02336.6 | UR | N | isoflavone reductase family protein [Talaromyces stipitatus ATCC 10500] | EED18078 |
|  | MGG_10751.6 | UR | N | peroxisomal copper amine oxidase [Neurospora crassa OR74A] | XP_960480 |
|  | MGG_13262.6 | UR | Y | 6-hydroxy-D-nicotine oxidase, putative [Aspergillus flavus NRRL3357] | EED53119 |
|  | MGG_13764.6 | UR | N | Bilirubin oxidase[Myrothecium verrucaria] | Q12737 |
|  | MGG_05084.6 | DR | N | NAD(P)H-dependent FMN reductase LOT6, putative [Aspergillus flavus NRRL3357] | EED54383 |
|  | MGG_01353.6 | DR | N | cytochrome P450 monooxygenase [Pyrenophora tritici-repentis Pt-1C-BFP] | XP_001932099 |
|  | MGG_14010.6 | DR | N | acyl-Coenzyme A binding domain containing 6 [Rattus norvegicus] | NP_001011906 |
|  | MGG_05854.6 | DR | N | cytochrome P450 monooxygenase, putative [Aspergillus clavatus NRRL 1] | XP_001271830 |
|  | MGG_08046.6 | DR | Y | bilirubin oxidase precursor [Neurospora crassa OR74A] | XP_956350 |
|  | MGG_08349.6 | DR | N | short-chain dehydrogenase, putative [Talaromyces stipitatus ATCC10500] | EED21280 |
|  | MGG_08498.6 | DR | Y | cytochrome P450 monooxygenase [Gibberella zeae] | BAD98712 |
|  | MGG_03828.6 | DR | N | cytochrome P450, putative [Neosartorya fischeri NRRL 181] | XP_001266395 |
|  | MGG_01544.6 | DR | Y | cytochrome P450 monooxygenase, putative [Magnaporthe grisea 70-15] | XP_367715 |
|  | MGG_11663.6 | DR | N | zinc-binding oxidoreductase CipB [Pyrenophora tritici-repentis Pt-1C-BFP] | XP_001930726 |
|  | MGG_07626.6 | DR | Y | cytochrome P450 monooxygenase [Botryotinia fuckeliana] | CAE76652 |
|  | MGG_11608.6 | DR | Y | laccase [Gaeumannomyces graminis var. tritici] | CAD10747 |
|  | MGG_13464.6 | DR | Y | laccase [Gaeumannomyces graminis var. tritici] | CAD10749 |
|  | MGG_02818.6 | DR | Y | FAD binding domain protein [Neosartorya fischeri NRRL 181] | XP_001262117 |
|  | MGG_00402.6 | DR | N | short-chain dehydrogenase, putative [Penicillium marneffei ATCC 18224] | XP_002149745 |
|  | MGG_07626.6 | DR | Y | cytochrome P450 monooxygenase [Botryotinia fuckeliana] | CAE76652 |
|  | MGG_09189.6 | DR | Y | choline dehydrogenase [Pyrenophora tritici-repentis Pt-1C-BFP] | XP_001937164 |
|  | MGG_10239.6 | DR | N | thymine dioxygenase [Pyrenophora tritici-repentis Pt-1C-BFP] | XP_001936794 |
|  | MGG_08349.6 | DR | N | short-chain dehydrogenase, putative [Talaromyces stipitatus ATCC10500] | EED21280 |
|  | MGG_10907.6 | DR | N | FAD-dependent oxygenase, putative [Aspergillus flavus NRRL3357] | EED53712 |
|  | MGG_05010.6 | DR | N | NAD dependent epimerase/dehydratase, putative [Aspergillus flavus NRRL3357] | EED54484 |
|  | MGG_09632.6 | DR | N | oxidoreductase, zinc-binding dehydrogenase family superfamily [Penicillium marneffei ATCC 18224] | XP_002149740 |
|  | MGG_10083.6 | DR | N | endoglucanase 3 precursor [Neurospora crassa OR74A] | XP_964159 |
|  | MGG_05457.6 | DR | N | taurine catabolism dioxygenase TauD, TfdA family protein[Neosartorya fischeri NRRL 181] | XP_001266665 |
|  | MGG_07587.6 | DR | N | isoflavone reductase family protein [Aspergillus clavatus NRRL1] | XP_001274110 |
|  | MGG_07982.6 | DR | N | cytochrome P450 monoxygenase [Botryotinia fuckeliana] | CAH64679 |
|  | MGG_13573.6 | DR | N | FAD binding oxidoreductase, putative [Aspergillus fumigatus A1163] | EDP50847 |
|  | MGG_08072.6 | DR | N | related to cholesterol oxidase precursor [Neurospora crassa] | CAD21388 |
|  | MGG_09188.6 | DR | Y | malate dehydrogenase [Aspergillus fumigatus Af293] | XP_755713 |
|  | MGG_05826.6 | DR | N | epoxide hydrolase [Stigmatella aurantiaca DW4/3-1] | ZP_01461444 |
|  | MGG_00889.6 | DR | N | putative DNA cytosine methyltransferase [Sordaria macrospora] | CAJ40945 |
|  | MGG_07602.6 | DR | N | phosphoprotein phosphatase (predicted) [Schizosaccharomyces pombe 972h-] | NP_595700 |
|  | MGG_11075.6 | DR | N | cytochrome P450 monooxygenase [Gibberella fujikuroi] | CAA75566 |
|  | MGG_02210 | DR | N | Chain A, Crystal Structure Of A Trapped Phosphate-Intermediate In Vanadium Apochloroperoxidase Catalyzing A Dephosphorylation Reaction.[Curvularia Inaequalis] | 3BB0_A |
| **Metabolism (GO:0008152)** | | | | | |
|  | MGG_05798.6 | UR | Y | cutinase precursor [Pyrenophora tritici-repentis Pt-1C-BFP] | XP_001935104 |
|  | MGG_04944.6 | UR | Y | malate dehydrogenase [Aspergillus fumigatus Af293] | XP_755713 |
|  | MGG_10497.6 | UR | N | bli-3 protein [Pyrenophora tritici-repentis Pt-1C-BFP] | XP_001941956 |
|  | MGG_00931.6 | UR | N | alpha/beta hydrolase fold [Ochrobactrum anthropi ATCC 49188] | YP_001369382 |
|  | MGG_10005.6 | UR | N | glycerol kinase, putative [Talaromyces stipitatus ATCC 10500] | EED22001 |
|  | MGG_01692.6 | UR | Y | carbonic anhydrase, putative [Penicillium marneffei ATCC 18224] | XP_002148277 |
|  | MGG_10005.6 | UR | N | glycerol kinase, putative [Talaromyces stipitatus ATCC 10500] | EED22001 |
|  | MGG_10671.6 | UR | N | Isoprenoid Biosynthesis enzymes, Class 1[Botryotinia fuckeliana] | AAQ16575 |
|  | MGG_11860.6 | UR | N | NACHT and WD domain protein [Aspergillus fumigatus Af293] | XP_754863 |
|  | MGG_02072.6 | UR | N | Amino-acid permease inda1[Trichoderma atroviride] | P34054 |
|  | MGG_09072.6 | UR | N | alcohol oxidase p68 [Cochliobolus victoriae] | AAK14990 |
|  | MGG_00806.6 | UR | N | polyketide synthase [Xylaria sp. BCC 1067] | AAY40862 |
|  | MGG_09668.6 | UR | N | carboxylesterase, putative [Talaromyces stipitatus ATCC 10500] | EED17829 |
|  | MGG_08589.6 | DR | Y | hydrolase, alpha/beta fold family protein [Stigmatella aurantiaca DW4/3-1] | ZP_01462220 |
|  | MGG_09836.6 | DR | N | NAD dependent epimerase/dehydratase, putative [Aspergillus flavus NRRL3357] | EED47405 |
|  | MGG_05803.6 | DR | N | ankyrin, putative [Talaromyces stipitatus ATCC 10500] | EED22530 |
|  | MGG_07571.6 | DR | Y | LysM domain protein [Neosartorya fischeri NRRL 181] | XP_001257349 |
|  | MGG_09785.6 | DR | N | short-chain dehydrogenase, putative [Aspergillus flavus NRRL3357 | EED51778 |
|  | MGG_02787.6 | DR | N | similar to CG5065 CG5065-PA [Tribolium castaneum] | XP_967752 |
|  | MGG_08989.6 | DR | N | short chain dehydrogenase (AtsC), putative [Aspergillus flavus NRRL3357] | EED51702 |
|  | MGG_08660.6 | DR | N | putative sialidase [Trichophyton rubrum] | ABG67894 |
|  | MGG_05759.6 | DR | N | related to hxB protein [Neurospora crassa] | CAB97294 |
|  | MGG_14692.6 | DR | Y | mutanase [Aspergillus fumigatus A1163] | EDP49885 |
|  | MGG_09945.6 | DR | N | cytochrome P450 46A1 [Pyrenophora tritici-repentis Pt-1C-BFP] | XP_001938675 |
|  | MGG_05632.6 | DR | Y | siderophore biosynthesis enzyme, putative [Talaromyces stipitatus ATCC 10500] | EED19426 |
|  | MGG_10913.6 | DR | N | short-chain dehydrogenase, putative [Talaromyces stipitatus ATCC10500] | EED21280 |
|  | MGG_10631.6 | DR | N | glycoside hydrolase family 24 protein [Laccaria bicolor S238N-H82] | XP_001887554 |
|  | MGG_01544.6 | DR | N | cytochrome P450 monooxygenase, putative [Magnaporthe grisea 70-15] | XP_367715 |
| **Response to stress (GO:0006965)** | | | | | |
|  | MGG_07790.6 | DR | Y | ligninase H2 precursor [Pyrenophora tritici-repentis Pt-1C-BFP] | XP_001933374 |
|  | MGG_11754.6 | DR | N | heavy metal tolerance protein precursor [Pyrenophora tritici-repentis Pt-1C-BFP] | XP_001936908 |
|  | MGG_09834.6 | DR | Y | peroxidase/catalase [Gibberella moniliformis] | ACJ72867 |
| **Regulation of transcription (GO:0006355)** | | | | | |
|  | MGG_07517.6 | UR | N | white collar, putative [Aspergillus flavus NRRL3357] | EED45439 |
|  | MGG_15023.6 | UR | N | C6 finger domain protein, putative [Talaromyces stipitatus ATCC10500] | EED21828 |
|  | MGG_02866.6 | UR | N | nitrogen assimilation transcription factor nirA [Aspergillus terreus NIH2624] | XP_001211251 |
|  | MGG_10422.6 | DR | N | C6 transcription factor OefC [Aspergillus flavus NRRL3357] | EED57819 |
|  | MGG_07314.6 | DR | N | C2H2 finger domain protein, putative [Aspergillus clavatus NRRL 1] | XP_001275629 |
|  | MGG_07305.6 | DR | N | bZIP transcription factor (Fcr3), putative [Talaromyces stipitatus ATCC 10500] | EED20182 |
|  | MGG_00587.6 | DR | N | serine/threonine-protein kinase ripk4, putative [Penicillium marneffei ATCC 18224] | XP_002146313 |
|  | MGG_07218.6 | DR | N | transcription factor [Colletotrichum lagenarium] | BAE98094 |
|  | MGG_10422.6 | DR | N | C6 transcription factor OefC [Aspergillus flavus NRRL3357] | EED57819 |
|  | MGG_03133.6 | DR | N | potential zinc finger transcription factor [Candida albicans SC5314] | XP_712367 |
|  | MGG_07218.6 | DR | N | transcription factor [Colletotrichum lagenarium] | BAE98094 |
|  | MGG_14358.6 | DR | N | zinc finger protein [Aedes aegypti] | XP_001662330 |
| **Transport (GO:0006810)** | | | | | |
|  | MGG_10750.6 | UR | N | OPT oligopeptide transporter protein[Yarrowia lipolytica] | XP_501979 |
|  | MGG_15435.6 | UR | N | efflux pump antibiotic resistance protein, putative [Talaromyces stipitatus ATCC 10500] | EED19841 |
|  | MGG_12612.6 | UR | N | MFS multidrug transporter, putative [Talaromyces stipitatus ATCC10500] | EED14464 |
|  | MGG_10046.6 | UR | N | pantothenate transporter, putative [Talaromyces stipitatus ATCC10500] | EED19237 |
|  | MGG_05946.6 | UR | N | putative sugar transporter [Gibberella moniliformis] | ABV60281 |
|  | MGG_11611.6 | UR | N | amino acid transporter, putative [Penicillium marneffei ATCC18224] | XP_002153282 |
|  | MGG_04407.6 | UR | N | cation diffusion facilitator 10 [Pyrenophora tritici-repentis Pt-1C-BFP] | XP_001930927 |
|  | MGG_10481.6 | UR | N | NACHT and WD domain protein [Aspergillus fumigatus Af293] | XP_754863 |
|  | MGG_12612.6 | UR | N | MFS multidrug transporter, putative [Talaromyces stipitatus ATCC10500] | EED14464 |
|  | MGG_03349.6 | UR | N | Auxin Efflux Carrier superfamily [Aspergillus clavatus NRRL 1] | XP_001276302 |
|  | MGG_03706.6 | UR | Y | integral membrane protein [Penicillium marneffei ATCC 18224] | XP_002148740 |
|  | MGG_13669.6 | UR | N | MFS peptide transporter, putative [Aspergillus flavus NRRL3357] | EED49889 |
|  | MGG_03706.6 | UR | Y | integral membrane protein [Penicillium marneffei ATCC 18224] | XP_002148740 |
|  | MGG_11209.6 | UR | N | ABC transporter, putative [Aspergillus clavatus NRRL 1] | XP_001268636 |
|  | MGG_11496.6 | UR | N | sphingoid long-chain base transporter, putative [Talaromyces stipitatus ATCC 10500] | EED20880 |
|  | MGG_02346.6 | DR | N | sugar transporter, putative [Aspergillus flavus NRRL3357] | EED53463 |
|  | MGG_01511.6 | DR | N | a multdrug transfer [Monascus pilosus] | BAE44306 |
|  | MGG_01778.6 | DR | N | probable aflatoxin efflux pump AFLT [Neurospora crassa] | CAF06057 |
|  | MGG_07946.6 | DR | N | integral membrane protein, putative [Talaromyces stipitatus ATCC10500] | EED17823 |
|  | MGG_01764.6 | DR | Y | integral membrane protein [Talaromyces stipitatus ATCC 10500] | EED17189 |
|  | MGG_09931.6 | DR | N | ABC multidrug transporter, putative [Penicillium marneffei ATCC18224] | XP_002144372 |
|  | MGG_04864.6 | DR | Y | allantoin permease, putative [Talaromyces stipitatus ATCC 10500] | EED14067 |
|  | MGG_01511.6 | DR | N | a multdrug transfer [Monascus pilosus] | BAE44306 |
| **Pathogenicity (GO:0009405)** | | | | | |
|  | MGG_09667.6 | UR | N | plasma membrane protein Pth11, putative [Neosartorya fischeri NRRL 181] | XP_001261560 |
|  | MGG_10407.6 | DR | N | integral membrane protein (Pth11), putative [Aspergillus flavus NRRL3357] | EED45803 |
| **Others** | | | | | |
|  | MGG_05165.6 | UR | Y | actin filament organization protein App1-like [Aspergillus fumigatus Af293] | XP_750414 |
|  | MGG_09165.6 | UR | N | sialidase [Trichophyton equinum] | ACJ04076 |
|  | MGG_05499.6 | UR | N | XMEK kinase (MEKK) Mkh1 (PMID 9199286) [Schizosaccharomyces pombe 972h-] | NP_593005 |
|  | MGG_04076.6 | UR | N | low-density lipoprotein receptor YWTD repeat [Burkholderia sp. H160]. | ZP_03266143 |
|  | MGG_05767.6 | UR | N | Phosphorylase superfamily[Aspergillus flavus NRRL3357] | EED46522 |
|  | MGG_03082.6 | UR | N | Heterokaryon incompatibility protein (HET)[Neurospora crassa] | CAD70324 |
|  | MGG_10482.6 | UR | N | Protein Kinases, catalytic domain[Penicillium chrysogenum Wisconsin 54-1255] | CAP80182 |
|  | MGG_14657.6 | UR | N | methyltransferase type 12 [Salmonella enterica subsp. Enterica serovar Heidelberg str. SL486] | ZP_02666126 |
|  | MGG_02114.6 | UR | N | interferon-induced GTP-binding protein Mx2 [Pyrenophora tritici-repentis Pt-1C-BFP] | XP_001942266 |
|  | MGG_07676.6 | UR | N | chitin binding protein, putative [Aspergillus flavus NRRL3357] | EED52495 |
|  | MGG_04406.6 | UR | N | NACHT and Ankyrin domain protein [Aspergillus fumigatus Af293] | XP_747379 |
|  | MGG_07787.6 | UR | N | regulatory P domain-containing protein [Hahella chejuensis KCTC 2396] | YP_433708 |
|  | MGG_13598.6 | DR | Y | endothiapepsin precursor [Neurospora crassa OR74A] | XP_963600 |
|  | MGG_06326.6 | DR | N | vacuolar ATP synthase 16 kDa proteolipid subunit [Sclerotinia sclerotiorum 1980] | XP_001588693 |
|  | MGG_03806.6 | DR | Y | calmodulin-related protein, putative [Arabidopsis thaliana] | NP_179170 |
|  | MGG_03995.6 | DR | N | carboxypeptidase S1, putative [Aspergillus clavatus NRRL 1] | XP_001274058 |
|  | MGG_02647.6 | DR | Y | UVI-1 [Bipolaris oryzae] | BAA96293 |
|  | MGG_08850.6 | DR | N | Ryp1 [Ajellomyces capsulatus] | ABX74945 |
|  | MGG_12480.6 | DR | N | aromatic prenyl transferase [Neotyphodium lolii] | ABF20224 |
|  | MGG_12981.6 | DR | N | Cupin domain protein [Aspergillus flavus NRRL3357] | EED49549 |
|  | MGG_10237.6 | DR | Y | accumulation-associated protein [Staphylococcus epidermidis RP62A] | CAB77251 |
|  | MGG_03364.6 | DR | N | secreted protein [Streptomyces sviceus ATCC 29083] | YP_002206261 |
|  | MGG_12480.6 | DR | N | aromatic prenyl transferase [Neotyphodium lolii] | ABF20224 |
|  | MGG_09384.6 | DR | N | LysR family regulatory protein, putative [Talaromyces stipitatus ATCC 10500] | EED19052 |
|  | MGG_04209.6 | DR | Y | related to exo-alpha-sialidase / neuraminidase [Neurospora crassa] | CAD70852 |
|  | MGG_15081.6 | DR | N | UMTA methyltransferase family protein [Aspergillus flavus NRRL3357] | EED50662 |
|  | MGG_03526.6 | DR | N | N-6 adenine-specific DNA methyltransferase 2 [Saccharomyces cerevisiae YJM789] | EDN61601 |
|  | MGG_05596.6 | DR | N | hydrolase, carbon-nitrogen family protein [Aspergillus clavatus NRRL 1] | XP_001274249 |
|  | MGG_05101.6 | DR | N | NmrA-like family protein [Neosartorya fischeri NRRL 181] | XP_001260891 |
|  | MGG_07861.6 | DR | Y | carboxypeptidase S1, putative [Penicillium marneffei ATCC 18224] | XP_002147986 |
|  | MGG_06326.6 | DR | N | vacuolar ATP synthase 16 kDa proteolipid subunit [Sclerotinia sclerotiorum 1980] | XP_001588693 |
|  | MGG_07946.6 | DR | N | integral membrane protein, putative [Talaromyces stipitatus ATCC10500] | EED17823 |
|  | MGG_03806.6 | DR | Y | calmodulin-related protein, putative [Arabidopsis thaliana] | NP_179170 |
|  | MGG_05632.6 | DR | Y | siderophore biosynthesis enzyme, putative [Talaromyces stipitatus ATCC 10500] | EED19426 |
