# Supplementary material for: Two Phosphodiesterase Genes, PDEL and PDEH, Regulate Development and Pathogenicity by Modulating Intracellular Cyclic AMP Levels in Magnaporthe oryzae
Source: PLoS One. 2011 Feb 28;6(2):e17241. doi: 10.1371/journal.pone.0017241 (PMC3046207; doi:10.1371/journal.pone.0017241)
Supplement: Table S3 — Categorization of PDEH regulated genes with known function. (DOC) [file pone.0017241.s005.doc]

| **Table S3. Categorization of *PDEH* regulated genes with known function** | | | | | | |
| --- | --- | --- | --- | --- | --- | --- |
| **Category** | **Gene ID** | **Exp.** | **Signal P** | | **Blast hit** | **NCBI_ID** |
| **Melanin biosynthesis (GO:0042438)** | | | | | | |
|  | MGG_07216.6 | DR | | N | Chain A, Crystal Structure Of 1,3,6,8-Tetrahydroxynaphthalene Reductase In Complex With Nadph And Pyroquilon[Magnaporthe grisea] | 1JA9_A |
| **Signal transduction (GO:0007165)** | | | | | | |
| **Amino Acid Metabolism (GO:0006520)** | | | | | | |
| **Proteolysis (GO:0006508)** | | | | | | |
|  | MGG_02531.6 | UR | | Y | subtilisin-like serine protease [Verticillium dahliae] | AAS45251 |
|  | MGG_10459.6 | UR | | Y | aspartic-type endopeptidase, putative [Talaromyces stipitatus ATCC10500] | EED14742 |
|  | MGG_00822.6 | DR | | N | V8-like Glu-specific endopeptidase [Magnetospirillum magnetotacticum] | ZP_00208343 |
|  | MGG_05803.6 | DR | | N | ankyrin, putative [Talaromyces stipitatus ATCC 10500] | EED22530 |
|  | MGG_00578.6 | DR | | Y | protease [Streptomyces sp. Mg1] | YP_002177821 |
|  | MGG_00311.6 | DR | | Y | acid protease [Pyrenophora tritici-repentis Pt-1C-BFP] | XP_001932923 |
|  | MGG_05855.6 | DR | | N | proteinase, putative [Talaromyces stipitatus ATCC 10500] | EED18776 |
|  | MGG_03817.6 | DR | | Y | metalloprotease [Pleurotus ostreatus] | AAU94648 |
| **Carbohydrate Metabolism (GO:0005975)** | | | | | | |
|  | MGG_12291.6 | UR | | Y | beta-hexosaminidase, putative [Aspergillus flavus NRRL3357] | EED53191 |
|  | MGG_03508.6 | UR | | Y | Glycosyl hydrolase family 3 N terminal domain Cel3e [Hypocrea jecorina] | AAP57760 |
|  | MGG_01542.6 | UR | | Y | putative endo-beta-1,4-D-xylanase precursor [Magnaporthe grisea] | AAC41684 |
|  | MGG_08424.6 | UR | | Y | endo-1,4-betaxylanase [Ascochyta pisi] | CAA93120 |
|  | MGG_08020.6 | UR | | N | endoglucanase II [Pyrenophora tritici-repentis Pt-1C-BFP] | XP_001933274 |
|  | MGG_07631.6 | UR | | N | endoglucanase [Aspergillus fumigatus Af293] | XP_755787 |
|  | MGG_00863.6 | DR | | Y | cell wall glucanase (Scw4), putative [Penicillium marneffei ATCC18224] | XP_002147497 |
|  | MGG_06593.6 | DR | | Y | endoxylanase II; pI 9 [Hypocrea jecorina] | AAB29346 |
|  | MGG_08232.6 | DR | | Y | LPS glycosyltransferase [Aspergillus fumigatus Af293] | XP_747140 |
| **Lipid metabolism (GO:0006629)** | | | | | | |
|  | MGG_03081.6 | UR | | N | LPS glycosyltransferase [Aspergillus fumigatus Af293] | XP_747140 |
|  | MGG_10477.6 | UR | | Y | esterase [Aspergillus fumigatus Af293] | XP_750067 |
|  | MGG_04194.6 | DR | | Y | acetyl esterase [Hypocrea jecorina] | ABI34466 |
|  | MGG_12214.6 | DR | | N | polyketide synthase [Gibberella moniliformis] | AAR92209 |
|  | MGG_02543.6 | DR | | Y | FG-GAP repeat domain-containing protein [Streptomyces sviceus ATCC29083] | YP_002205181 |
|  | MGG_01369.6 | DR | | N | hormone-sensitive lipase [Magnaporthe grisea] | ABG79927 |
|  | MGG_02933.6 | DR | | N | DHHC zinc finger membrane protein [Aspergillus fumigatus A1163] | EDP51616 |
|  | MGG_05788.6 | DR | | Y | ferulic acid esterase (FaeA), putative [Aspergillus flavus NRRL3357] | EED57501 |
| **Cell development (GO:0007275)** | | | | | | |
|  | MGG_00703.6 | UR | | Y | Gas1-like protein [Monacrosporium haptotylum] | AAU06196 |
|  | MGG_15113.6 | DR | | N | aldo/keto reductase, putative [Talaromyces stipitatus ATCC 10500] | EED22571 |
|  | MGG_00832.6 | DR | | Y | related to cytochrome p450 [imported] - Neurospora crassa | T49758 |
|  | MGG_14692.6 | DR | | Y | mutanase [Aspergillus fumigatus A1163] | EDP49885 |
|  | MGG_05824.6 | DR | | N | UDP-glucuronosyl/UDP-glucosyltransferase [Mycobacterium vanbaalenii PYR-1] | YP_955665 |
|  | MGG_06862.6 | DR | | N | LEA domain containing protein [Pyrenophora tritici-repentis Pt-1C-BFP] | XP_001938142 |
|  | MGG_09830.6 | DR | | N | AAA family ATPase, putative [Aspergillus clavatus NRRL 1] | XP_001273411 |
|  | MGG_05232.6 | DR | | Y | IgE-binding protein [Aspergillus fumigatus Af293] | XP_731512 |
| **Electron transport (GO:0006118)** | | | | | | |
|  | MGG_07356.6 | UR | | Y | isoamyl alcohol oxidase [Aspergillus fumigatus Af293] | XP_746836 |
|  | MGG_02336.6 | UR | | N | isoflavone reductase family protein [Talaromyces stipitatus ATCC10500] | EED18078 |
|  | MGG_07949.6 | UR | | N | choline dehydrogenase [Pyrenophora tritici-repentis Pt-1C-BFP] | XP_001937164 |
|  | MGG_02337.6 | UR | | N | 3-oxoacyl-[acyl-carrier-protein] reductase, putative [Penicillium marneffei ATCC 18224] | XP_002144230 |
|  | MGG_13764.6 | UR | | N | Bilirubin oxidase[Myrothecium verrucaria] | Q12737 |
|  | MGG_02336.6 | UR | | N | isoflavone reductase family protein [Talaromyces stipitatus ATCC10500] | EED18078 |
|  | MGG_12275.6 | UR | | Y | rds1p-like protein [Melampsora medusae f. sp. deltoidis] | ABS86600 |
|  | MGG_10961.6 | DR | | Y | FAD-dependent oxidase, putative [Penicillium marneffei ATCC 18224] | XP_002145721 |
|  | MGG_10907.6 | DR | | N | FAD-dependent oxygenase, putative [Aspergillus flavus NRRL3357] | EED53712 |
|  | MGG_13464.6 | DR | | Y | laccase [Gaeumannomyces graminis var. tritici] | CAD10749 |
|  | MGG_11608.6 | DR | | Y | laccase [Gaeumannomyces graminis var. tritici]. | CAD10747 |
|  | MGG_01544.6 | DR | | N | cytochrome P450 monooxygenase, putative [Magnaporthe grisea 70-15]. | XP_367715 |
|  | MGG_13764.6 | DR | | N | Bilirubin oxidase[Myrothecium verrucaria] | Q12737 |
|  | MGG_02818.6 | DR | | Y | FAD binding domain protein [Neosartorya fischeri NRRL 181]. | XP_001262117 |
|  | MGG_09189.6 | DR | | Y | choline dehydrogenase [Pyrenophora tritici-repentis Pt-1C-BFP]. | XP_001937164 |
|  | MGG_08349.6 | DR | | N | short-chain dehydrogenase, putative [Talaromyces stipitatus ATCC10500]. | EED21280 |
|  | MGG_11663.6 | DR | | N | zinc-binding oxidoreductase CipB [Pyrenophora tritici-repentis Pt-1C-BFP]. | XP_001930726 |
|  | MGG_11075.6 | DR | | N | cytochrome P450 monooxygenase [Gibberella fujikuroi]. | CAA75566 |
|  | MGG_00276.6 | DR | | N | FAD binding domain protein [Neosartorya fischeri NRRL 181]. | XP_001258713 |
|  | MGG_08046.6 | DR | | Y | bilirubin oxidase precursor [Neurospora crassa OR74A]. | XP_956350 |
|  | MGG_02792.6 | DR | | Y | related to n-alkane-inducible cytochrome P450 [Neurospora crassa]. | CAC10088 |
|  | MGG_12228.6 | DR | | N | alcohol dehydrogenase, putative [Penicillium marneffei ATCC 18224]. | XP_002144973 |
|  | MGG_04345.6 | DR | | N | pisatin demethylase [Pyrenophora tritici-repentis Pt-1C-BFP] | XP_001939116 |
|  | MGG_07626.6 | DR | | Y | cytochrome P450 monooxygenase [Botryotinia fuckeliana]. | CAE76652 |
|  | MGG_15026.6 | DR | | Y | glucose-methanol-choline (gmc) oxidoreductase, putative [Aspergillus flavus NRRL3357]. | EED49404 |
|  | MGG_08494.6 | DR | | Y | putative cytochrome P450 [Fusarium heterosporum]. | AAV66104 |
|  | MGG_10792.6 | DR | | N | salicylate hydroxylase, putative [Aspergillus clavatus NRRL 1]. | XP_001273741 |
|  | MGG_04751.6 | DR | | N | monooxygenase, putative [Talaromyces stipitatus ATCC 10500]. | EED21311 |
|  | MGG_08072.6 | DR | | N | related to cholesterol oxidase precursor [Neurospora crassa]. | CAD21388 |
|  | MGG_08349.6 | DR | | N | short-chain dehydrogenase, putative [Talaromyces stipitatus ATCC10500]. | EED21280 |
|  | MGG_04404.6 | DR | | Y | pisatin demethylase [Pyrenophora tritici-repentis Pt-1C-BFP]. | XP_001938784 |
|  | MGG_11663.6 | DR | | N | zinc-binding oxidoreductase CipB [Pyrenophora tritici-repentis Pt-1C-BFP]. | XP_001930726 |
|  | MGG_01297.6 | DR | | N | flavin-nucleotide-binding protein [Pseudovibrio sp. JE062]. | EEA96114 |
|  | MGG_15026.6 | DR | | N | glucose-methanol-choline (gmc) oxidoreductase, putative [Aspergillus flavus NRRL3357]. | EED49404 |
|  | MGG_10792.6 | DR | | N | salicylate hydroxylase, putative [Aspergillus clavatus NRRL 1]. | XP_001273741 |
| **Metabolism (GO:0008152)** | | | | | | |
|  | MGG_01986.6 | UR | | Y | short-chain dehydrogenase/reductase [Aspergillus fumigatus Af293]. | XP_748530 |
|  | MGG_00806.6 | UR | | N | polyketide synthase [Xylaria sp. BCC 1067]. | AAY40862 |
|  | MGG_07881.6 | UR | | N | GA15419 [Drosophila pseudoobscura pseudoobscura]. | XP_001359800 |
|  | MGG_10275.6 | UR | | N | acid sphingomyelinase, putative [Penicillium marneffei ATCC 18224]. | XP_002145882 |
|  | MGG_05759.6 | UR | | N | related to hxB protein [Neurospora crassa]. | CAB97294 |
|  | MGG_09836.6 | UR | | N | NAD dependent epimerase/dehydratase, putative [Aspergillus flavus NRRL3357]. | EED47405 |
|  | MGG_11636.6 | DR | | N | ankyrin repeat domain containing protein [Pyrenophora tritici-repentis Pt-1C-BFP]. | XP_001939919 |
|  | MGG_07571.6 | DR | | Y | LysM domain protein [Neosartorya fischeri NRRL 181]. | XP_001257349 |
|  | MGG_10631.6 | DR | | N | glycoside hydrolase family 24 protein [Laccaria bicolor S238N-H82]. | XP_001887554 |
|  | MGG_12983.6 | DR | | N | short-chain dehydrogenase, putative [Talaromyces stipitatus ATCC10500]. | EED21264 |
|  | MGG_10547.6 | DR | | N | ankyrin repeat-containing protein, putative [Penicillium marneffeiATCC 18224]. | XP_002151313 |
|  | MGG_12983.6 | DR | | N | short-chain dehydrogenase, putative [Talaromyces stipitatus ATCC10500]. | EED21264 |
|  | MGG_09785.6 | DR | | N | short-chain dehydrogenase, putative [Aspergillus flavus NRRL3357]. | EED51778 |
|  | MGG_10913.6 | DR | | N | short-chain dehydrogenase, putative [Talaromyces stipitatus ATCC10500]. | EED21280 |
|  | MGG_08989.6 | DR | | N | short chain dehydrogenase (AtsC), putative [Aspergillus flavus NRRL3357]. | EED51702 |
|  | MGG_05759.6 | DR | | N | related to hxB protein [Neurospora crassa]. | CAB97294 |
|  | MGG_10412.6 | DR | | N | allantoinase [Neurospora crassa OR74A]. | XP_959940 |
|  | MGG_09945.6 | DR | | N | cytochrome P450 46A1 [Pyrenophora tritici-repentis Pt-1C-BFP]. | XP_001938675 |
|  | MGG_08589.6 | DR | | Y | hydrolase, alpha/beta fold family protein [Stigmatella aurantiaca DW4/3-1]. | ZP_01462220 |
| **Response to stress (GO:0006965)** | | | | | | |
|  | MGG_03329.6 | UR | | N | small heat shock protein [Hypocrea lixii]. | AAX55622 |
|  | MGG_10368.6 | UR | | N | cytochrome c peroxidase, mitochondrial precursor [Ajellomyces capsulatus NAm1]. | XP_001536337 |
|  | MGG_07790.6 | DR | | Y | ligninase H2 precursor [Pyrenophora tritici-repentis Pt-1C-BFP]. | XP_001933374 |
| **Regulation of transcription (GO:0006355)** | | | | | | |
|  | MGG_07305.6 | UR | | N | bZIP transcription factor (Fcr3), putative [Talaromyces stipitatus ATCC 10500]. | EED20182 |
|  | MGG_10422.6 | DR | | N | C6 transcription factor OefC [Aspergillus flavus NRRL3357]. | EED57819 |
|  | MGG_14358.6 | DR | | N | zinc finger protein [Aedes aegypti]. | XP_001662330 |
|  | MGG_07218.6 | DR | | N | transcription factor [Colletotrichum lagenarium]. | BAE98094 |
|  | MGG_03133.6 | DR | | N | potential zinc finger transcription factor [Candida albicans SC5314]. | XP_712367 |
|  | MGG_03977.6 | DR | | N | Zinc-finger transcription factor, involved in induction of CLN3 transcription in response to glucose; genetic and physical interactions indicate a possible role in mitochondrialtranscription or genome maintenance; Azf1p [Saccharomyces cerevisiae]. | NP_014756 |
| **Transport (GO:0006810)** | | | | | | |
|  | MGG_15435.6 | UR | | N | efflux pump antibiotic resistance protein, putative [Talaromyces stipitatus ATCC 10500]. | EED19841 |
|  | MGG_10783.6 | UR | | N | aquaporin [Pyrenophora tritici-repentis Pt-1C-BFP]. | XP_001934329 |
|  | MGG_03706.6 | UR | | Y | integral membrane protein [Penicillium marneffei ATCC 18224]. | XP_002148740 |
|  | MGG_02885.6 | UR | | N | membrane protein, putative [Rhodobacterales bacterium HTCC2654]. | ZP_01015675 |
|  | MGG_09354.6 | UR | | N | oligopeptide transporter, putative [Penicillium marneffei ATCC18224]. | XP_002149499 |
|  | MGG_10750.6 | UR | | N | OPT oligopeptide transporter protein,YALI0C18491p [Yarrowia lipolytica]. | XP_501979 |
|  | MGG_03360.6 | DR | | N | related to carboxylic acid transport protein JEN1 [Neurospora crassa]. | CAB88550 |
|  | MGG_10293.6 | DR | | N | sugar transporter, putative [Aspergillus flavus NRRL3357]. | EED53374 |
|  | MGG_04852.6 | DR | | N | P-type ATPase [Schizosaccharomyces pombe]. | XP_001713045 |
|  | MGG_01778.6 | DR | | N | probable aflatoxin efflux pump AFLT [Neurospora crassa]. | CAF06057 |
|  | MGG_07639.6 | DR | | N | excitatory amino acid transporter 1 [Pyrenophora tritici-repentis Pt-1C-BFP]. | XP_001930712 |
|  | MGG_03123.6 | DR | | N | MATE efflux family protein subfamily, putative [Aspergillus clavatus NRRL 1]. | XP_001268211 |
|  | MGG_04251.6 | DR | | Y | sodium/phosphate symporter [Aspergillus fumigatus Af293]. | XP_748875 |
|  | MGG_07494.6 | DR | | N | cation diffusion facilitator 1 [Aspergillus clavatus NRRL 1]. | XP_001268816 |
|  | MGG_07606.6 | DR | | N | dicarboxylic amino acid permease [Aspergillus terreus NIH2624]. | XP_001218343 |
|  | MGG_02346.6 | DR | | N | sugar transporter, putative [Aspergillus flavus NRRL3357] | EED53463 |
|  | MGG_01511.6 | DR | | N | a multdrug transfer [Monascus pilosus]. | BAE44306 |
|  | MGG_01764.6 | DR | | Y | integral membrane protein [Talaromyces stipitatus ATCC 10500]. | EED17189 |
|  | MGG_09941.6 | DR | | N | ABC transporter [Aspergillus fumigatus Af293]. | XP_753691 |
|  | MGG_02093.6 | DR | | N | MFS multidrug transporter, putative [Aspergillus clavatus NRRL 1]. | XP_001275966 |
|  | MGG_08918.6 | DR | | N | ABC transporter, putative [Talaromyces stipitatus ATCC 10500]. | EED19262 |
| **Pathogenicity (GO:0009405)** | | | | | | |
|  | MGG_10315.6 | DR | | N | hydrophobin MPG1 [Magnaporthe oryzae]. | AAX53646 |
|  | MGG_05871.6 | DR | | Y | integral membrane protein PTH11 [Magnaporthe grisea] | AAD30436 |
| **Others** | | | | | | |
|  | MGG_07452.6 | UR | | N | GPI transamidase component PIG-S, putative [Penicillium marneffei ATCC 18224]. | XP_002150637 |
|  | MGG_00992.6 | UR | | Y | N-glycosylation site corresponding to basepairs 208-210 [Blumeria graminis]. | AAB05211 |
|  | MGG_07632.6 | UR | | Y | endonuclease/exonuclease/phosphatase family protein [Aspergillus fumigatus Af293]. | XP_756020 |
|  | MGG_09467.6 | UR | | Y | integral membrane protein, putative [Neosartorya fischeri NRRL181] | XP_001264348 |
|  | MGG_03364.6 | DR | | N | candidate effector 16 [Venturia inaequalis]. | ACM90105 |
|  | MGG_04631.6 | DR | | N | pentalenene synthase, putative [Aspergillus flavus NRRL3357]. | EED51984 |
|  | MGG_02647.6 | DR | | N | UVI-1 [Bipolaris oryzae]. | BAA96293 |
|  | MGG_03826.6 | DR | | Y | kelch repeat-containing protein [Methylobacterium extorquens PA1]. | YP_001638060 |
|  | MGG_02933.6 | DR | | N | DHHC zinc finger membrane protein [Aspergillus fumigatus A1163]. | EDP51616 |
|  | MGG_11305.6 | DR | | Y | YqcI/YcgG family [Rhodobacterales bacterium Y4I]. | EDZ48499 |
|  | MGG_04209.6 | DR | | Y | related to exo-alpha-sialidase / neuraminidase [Neurospora crassa]. | CAD70852 |
|  | MGG_03501.6 | DR | | N | DUF1264 domain protein [Penicillium marneffei ATCC 18224]. | XP_002145074 |
|  | MGG_06538.6 | DR | | Y | BYS1 domain protein, putative [Aspergillus fumigatus A1163]. | EDP51049 |
|  | MGG_08488.6 | DR | | N | Pfs domain protein [Aspergillus flavus NRRL3357]. | EED46522 |
|  | MGG_02648.6 | DR | | N | dynamin family GTPase, putative [Aspergillus fumigatus A1163]. | EDP55311 |
|  | MGG_04369.6 | DR | | N | AAA ATPase, putative [Aspergillus flavus NRRL3357]. | EED51143 |
|  | MGG_08495.6 | DR | | N | tol related protein [Magnaporthe grisea]. | BAD67182 |
|  | MGG_10480.6 | DR | | N | ankyrin repeat protein [Aspergillus fumigatus Af293]. | XP_752820 |
|  | MGG_09384.6 | DR | | N | LysR family regulatory protein, putative [Aspergillus clavatus NRRL1]. | XP_001275054 |
|  | MGG_07561.6 | DR | | N | Atu related protein [imported] - Neurospora crassa. | T49516 |
|  | MGG_06326.6 | DR | | N | vacuolar ATP synthase 16 kDa proteolipid subunit [Sclerotinia sclerotiorum 1980] | XP_001588693 |
|  | MGG_14010.6 | DR | | N | serine/threonine-protein kinase ripk4, putative [Penicillium marneffei ATCC 18224]. | XP_002146313 |
|  | MGG_05632.6 | DR | | Y | siderophore biosynthesis enzyme, putative [Talaromyces stipitatus ATCC 10500]. | EED19426 |
