# Supplementary material for: Two Phosphodiesterase Genes, PDEL and PDEH, Regulate Development and Pathogenicity by Modulating Intracellular Cyclic AMP Levels in Magnaporthe oryzae
Source: PLoS One. 2011 Feb 28;6(2):e17241. doi: 10.1371/journal.pone.0017241 (PMC3046207; doi:10.1371/journal.pone.0017241)
Supplement: Table S4 — Categorization of PDEL&PDEH regulated genes with known function. (DOC) [file pone.0017241.s006.doc]

| **Table S4. Categorization of *PDEL&PDEH* regulated genes with known function** | | | | | | |
| --- | --- | --- | --- | --- | --- | --- |
| **Category** | **Gene ID** | **Exp.** | **Signal P** | | **Blast hit** | **NCBI_ID** |
| **Melanin biosynthesis (GO:0042438)** | | | | | | |
|  | MGG_07216.6 | DR | | N | Chain A, Crystal Structure Of 1,3,6,8-Tetrahydroxynaphthalene Reductase In Complex With Nadph And Pyroquilon[Magnaporthe grisea] | 1JA9_A |
|  | MGG_07219.6 | DR | | N | polyketide synthase [Ophiostoma piceae] | ABD47522 |
|  | MGG_07218.6 | DR | | N | transcription factor [Colletotrichum lagenarium] | BAE98094 |
| **Signal transduction (GO:0007165)** | | | | | | |
|  | MGG_01094.6 | DR | | N | C2 domain protein [Penicillium marneffei ATCC 18224] | XP_002151241 |
|  | MGG_05664.6 | DR | | N | high affinity cAMP phosphodiesterase, putative [Aspergillus clavatus NRRL1]. | XP_001269639 |
|  | MGG_10544.6 | DR | | N | G protein-coupled receptor GprD [Aspergillus flavus NRRL3357] | EED50805 |
|  | MGG_06738.6 | DR | | N | G-protein coupled receptor [Trichoderma atroviride] | ABD46750 |
|  | MGG_09445.6 | DR | | N | similar to synaptotagmin, putative [Tribolium castaneum] | XP_974305 |
| **Amino Acid Metabolism (GO:0006520)** | | | | | | |
|  | MGG_03492.6 | DR | | N | acetamidase [Coccidioides immitis RS] | XP_001248603 |
|  | MGG_05024.6 | DR | | N | N-carbamoyl-L-amino acid hydrolase [Pyrenophora tritici-repentis Pt-1C-BFP] | XP_001935695 |
|  | MGG_10299.6 | DR | | N | L-lysine 2,3-aminomutase, putative [Aspergillus flavus NRRL3357] | EED47871 |
|  | MGG_10533.6 | DR | | Y | agmatinase [Aspergillus fumigatus Af293] | XP_753336 |
|  | MGG_02817.6 | DR | | N | glutamate decarboxylase [Neurospora crassa OR74A] | XP_965818 |
|  | MGG_04173.6 | DR | | N | EF-hand calcium-binding domain protein, putative [Aspergillus flavus NRRL3357]. | EED51602 |
|  | MGG_02812.6 | DR | | N | 1-aminocyclopropane-1-carboxylate deaminase, putative [Talaromyces stipitatus ATCC 10500] | EED13104 |
| **Proteolysis (GO:0006508)** | | | | | | |
|  | MGG_03056.6 | UR | | Y | aorsin endoprotease precursor [Laccaria bicolor S238N-H82] | XP_001876156 |
|  | MGG_03144.6 | UR | | N | Ulp1 protease family protein [Talaromyces stipitatus ATCC 10500] | EED13023 |
|  | MGG_09351.6 | UR | | Y | penicillopepsin [Neurospora crassa OR74A] | XP_959401 |
|  | MGG_00311.6 | DR | | Y | acid protease [Pyrenophora tritici-repentis Pt-1C-BFP] | XP_001932923 |
|  | MGG_13765.6 | DR | | Y | extracelular serine carboxypeptidase, putative [Talaromyces stipitatus ATCC 10500] | EED18491 |
|  | MGG_07877.6 | DR | | Y | secreted dipeptidyl peptidase [Neosartorya fischeri NRRL 181] | XP_001260402 |
|  | MGG_05128.6 | DR | | N | amino acid transporter (predicted) [Schizosaccharomyces pombe 972h-] | NP_588250 |
|  | MGG_08758.6 | DR | | Y | leupeptin-inactivating enzyme 1 precursor [Pyrenophora tritici-repentis Pt-1C-BFP] | NP_588250 |
|  | MGG_12773.6 | DR | | Y | leupeptin-inactivating enzyme 1 precursor [Pyrenophora tritici-repentis Pt-1C-BFP] | XP_001942412 |
|  | MGG_02275.6 | DR | | Y | serine peptidase, putative [Aspergillus clavatus NRRL 1] | XP_001273182 |
|  | MGG_02531.6 | DR | | Y | subtilisin-like serine protease [Verticillium dahliae] | AAS45251 |
|  | MGG_03817.6 | DR | | Y | metalloprotease [Pleurotus ostreatus] | AAU94648 |
|  | MGG_12068.6 | DR | | Y | regulatory P domain-containing protein [Hahella chejuensis KCTC 2396] | YP_433708 |
|  | MGG_05855.6 | DR | | N | proteinase, putative [Penicillium marneffei ATCC 18224] | XP_002152181 |
| **Carbohydrate Metabolism (GO:0005975)** | | | | | | |
|  | MGG_08424.6 | UR | | Y | endo-1,4-betaxylanase [Ascochyta pisi] | CAA93120 |
|  | MGG_01542.6 | UR | | Y | putative endo-beta-1,4-D-xylanase precursor [Magnaporthe grisea] |  |
|  | MGG_04732.6 | UR | | Y | chitinase [Coprinellus congregatus] | CAQ51152 |
|  | MGG_13801.6 | UR | | Y | beta-galactosidase [Pyrenophora tritici-repentis Pt-1C-BFP] | XP_001936859 |
|  | MGG_08331.6 | UR | | Y | endo-1,4-beta-xylanase (XlnA), putative [Neosartorya fischeri NRRL 181] | XP_001258638 |
|  | MGG_05364.6 | UR | | N | endoglucanase IV precursor [Neurospora crassa OR74A] | XP_958254 |
|  | MGG_07631.6 | UR | | N | endoglucanase [Aspergillus fumigatus Af293] | XP_755787 |
|  | MGG_06069.6 | UR | | N | endoglucanase, putative [Aspergillus clavatus NRRL 1] | XP_001275694 |
|  | MGG_08944.6 | UR | | Y | 1,4-alpha-glucan branching enzyme [Shewanella amazonensis SB2B] | YP_928327 |
|  | MGG_14243.6 | UR | | Y | putative xylanase 17 [Gibberella zeae] | AAT84255 |
|  | MGG_08066.6 | UR | | N | fungal cellulose binding domain containing protein [Pyrenophora tritici-repentis Pt-1C-BFP] | XP_001937817 |
|  | MGG_14061.6 | UR | | Y | oxalate decarboxylase, putative [Aspergillus flavus NRRL3357] | EED56627 |
|  | MGG_07868.6 | DR | | N | endo-1,4-beta-xylanase precursor [Aspergillus terreus NIH2624] | XP_001212588 |
|  | MGG_07884.6 | DR | | N | YALI0F01628p [Yarrowia lipolytica] | XP_504869 |
|  | MGG_09272.6 | DR | | Y | beta-glucosidase 1 precursor [Neurospora crassa OR74A] | XP_956104 |
|  | MGG_06593.6 | DR | | Y | endoxylanase II; pI 9 [Hypocrea jecorina] | AAB29346 |
|  | MGG_03287.6 | DR | | N | related to alpha-amylase [Neurospora crassa] | CAE75731 |
|  | MGG_09601.6 | DR | | Y | glycosyl hydrolase, putative [Aspergillus flavus NRRL3357] | EED57325 |
|  | MGG_12939.6 | DR | | Y | chitin binding protein [Magnaporthe grisea] | BAB79692 |
|  | MGG_08985.6 | DR | | Y | beta-xylosidase [Pyrenophora tritici-repentis Pt-1C-BFP] | XP_001937121 |
|  | MGG_09433.6 | DR | | N | glucanase, putative [Aspergillus clavatus NRRL 1] | XP_001273400 |
|  | MGG_01922.6 | DR | | N | polysaccharide deacetylase family protein [Pyrenophora tritici-repentis Pt-1C-BFP] | XP_001932774 |
|  | MGG_12983.6 | DR | | N | short-chain dehydrogenase, putative [Talaromyces stipitatus ATCC 10500] | EED21264 |
|  | MGG_05599.6 | DR | | Y | glycosyl hydrolase, putative [Neosartorya fischeri NRRL 181] | XP_001264012 |
|  | MGG_09664.6 | DR | | N | beta-mannosidase [Emericella nidulans] | ABF50864 |
|  | MGG_04172.6 | DR | | Y | glycosyl hydrolase [Pyrenophora tritici-repentis Pt-1C-BFP] | XP_001940096 |
|  | MGG_10189.6 | DR | | Y | beta-glucosidase [Neurospora crassa OR74A] | XP_956183 |
|  | MGG_08232.6 | DR | | Y | LPS glycosyltransferase [Aspergillus fumigatus Af293]. | XP_747140 |
|  | MGG_12939.6 | DR | | Y | chitin binding protein [Magnaporthe grisea] | BAB79692 |
|  | MGG_15452.6 | DR | | N | glycosyl hydrolase family 88, putative [Penicillium marneffei ATCC 18224] | XP_002143913 |
|  | MGG_09272.6 | DR | | Y | beta-glucosidase 1 precursor [Neurospora crassa OR74A] | XP_956104 |
|  | MGG_03880.6 | DR | | N | alcohol dehydrogenase 1 [Pyrenophora tritici-repentis Pt-1C-BFP] | XP_001939279 |
|  | MGG_09608.6 | DR | | N | coagulation factor 5/8 type domain protein [Geobacillus sp.Y412MC10] | ZP_03037141 |
|  | MGG_00050.6 | DR | | N | alpha-L-fucosidase 2 precursor, putative [Aspergillus flavus NRRL3357] | EED47314 |
| **Lipid metabolism (GO:0006629)** | | | | | | |
|  | MGG_03081.6 | UR | | N | LPS glycosyltransferase [Aspergillus fumigatus Af293] | XP_747140 |
|  | MGG_04075.6 | UR | | N | 3-hydroxyacyl-CoA dehydrogenase [Burkholderia sp. 383] | YP_372153 |
|  | MGG_04191.6 | UR | | N | heat shock 70 kDa protein, mitochondrial precursor [Neurospora crassa OR74A] | XP_961753 |
|  | MGG_04346.6 | UR | | N | sterol 24-C-methyltransferase-like protein [Magnaporthe grisea] | AAX07631 |
|  | MGG_04194.6 | DR | | Y | acetyl esterase [Hypocrea jecorina] | ABI34466 |
|  | MGG_01369.6 | DR | | N | hormone-sensitive lipase [Magnaporthe grisea] | ABG79927 |
|  | MGG_02543.6 | DR | | Y | FG-GAP repeat domain-containing protein [Streptomyces sviceus ATCC 29083] | YP_002205181 |
|  | MGG_06610.6 | DR | | Y | lipase [Streptomyces clavuligerus ATCC 27064] | YP_002193099 |
|  | MGG_07627.6 | DR | | N | homoserine acetyltransferase family protein [Pyrenophora tritici-repentis Pt-1C-BFP] | XP_001940705 |
|  | MGG_02933.6 | DR | | N | DHHC zinc finger membrane protein [Aspergillus fumigatus A1163] | EDP51616 |
| **Cell development (GO:0007275)** | | | | | | |
|  | MGG_06167.6 | UR | | Y | phytase [Aspergillus flavus NRRL3357] | EED53727 |
|  | MGG_06759.6 | UR | | Y | heat shock protein 90 [Humicola fuscoatra] | ACF93232 |
|  | MGG_08180.6 | UR | | N | DNAJ heat shock family protein [Pyrenophora tritici-repentis Pt-1C-BFP] | XP_001931291 |
|  | MGG_06759.6 | UR | | N | heat shock protein 90 [Humicola fuscoatra] | ACF93232 |
|  | MGG_03089.6 | UR | | N | Bax Inhibitor family protein [Neosartorya fischeri NRRL 181] | XP_001259759 |
|  | MGG_09527.6 | UR | | Y | endoglucanase, putative [Talaromyces stipitatus ATCC 10500]. | EED18911 |
|  | MGG_00408.6 | UR | | N | Ran exchange factor Prp20/Pim1, putative [Aspergillus clavatus NRRL1]. | XP_001274740 |
|  | MGG_08809.6 | UR | | N | YMR098Cp-like protein [Saccharomyces cerevisiae AWRI1631] | EDZ70131 |
|  | MGG_03329.6 | UR | | N | small heat shock protein [Hypocrea lixii] | AAX55622 |
|  | MGG_06862.6 | DR | | N | LEA domain containing protein [Pyrenophora tritici-repentis Pt-1C-BFP] | XP_001938142 |
|  | MGG_08537.6 | DR | | Y | endoglucanase [Humicola grisea] | AAM77714 |
|  | MGG_03038.6 | DR | | N | NB-ARC and TPR domain protein [Aspergillus flavus NRRL3357] | EED48493 |
|  | MGG_00194.6 | DR | | Y | protein rds1 [Neurospora crassa OR74A] | XP_956641 |
|  | MGG_00832.6 | DR | | Y | cytochrome P450 2C30 [Pyrenophora tritici-repentis Pt-1C-BFP] | XP_001941477 |
|  | MGG_09260.6 | DR | | Y | integral membrane protein [Aspergillus fumigatus Af293] | XP_753773 |
|  | MGG_09330.6 | DR | | Y | putative phosphatidic acid phosphatase [Pleurotus sp. 'Florida'] | CAD10795 |
|  | MGG_02110.6 | DR | | N | caleosin domain containing protein [Pyrenophora tritici-repentis Pt-1C-BFP] | XP_001932418 |
|  | MGG_02574.6 | DR | | N | NCP1 pseudogene [Cryptococcus neoformans var. neoformans] | AAN75605 |
|  | MGG_14692.6 | DR | | Y | mutanase [Aspergillus fumigatus A1163] | EDP49885 |
|  | MGG_08774.6 | DR | | Y | vi polysaccharide biosynthesis protein vipA/tviB [Pyrenophora tritici-repentis Pt-1C-BFP] | XP_001933886 |
|  | MGG_07005.6 | DR | | Y | CFEM domain protein [Aspergillus clavatus NRRL 1] | XP_001268442 |
| **Electron transport (GO:0006118)** | | | | | | |
|  | MGG_01986.6 | UR | | Y | short-chain dehydrogenase/reductase [Aspergillus fumigatus Af293] | XP_748530 |
|  | MGG_14956.6 | UR | | N | cytochrome b5 reductase, putative [Aspergillus flavus NRRL3357] | EED48869 |
|  | MGG_08487.6 | UR | | Y | cellobiose dehydrogenase, putative [Aspergillus flavus NRRL3357] | EED49453 |
|  | MGG_12722.6 | UR | | Y | isoamyl alcohol oxidase, putative [Aspergillus fumigatus A1163] | EDP48041 |
|  | MGG_02337.6 | UR | | N | 3-oxoacyl-[acyl-carrier-protein] reductase, putative [Penicillium marneffei ATCC 18224] | XP_002144230 |
|  | MGG_04349.6 | UR | | N | cytochrome P450, putative [Penicillium marneffei ATCC 18224] | XP_002149366 |
|  | MGG_07356.6 | UR | | Y | amino acid permease [Aspergillus fumigatus Af293] | XP_748191 |
|  | MGG_00246.6 | UR | | N | NADP-dependent alcohol dehydrogenase C [Pyrenophora tritici-repentis Pt-1C-BFP] | XP_001941135 |
|  | MGG_11936.6 | UR | | Y | FAD binding domain containing protein [Pyrenophora tritici-repentis Pt-1C-BFP] | XP_001941500 |
|  | MGG_03707.6 | UR | | N | quinone oxidoreductase [Pichia stipitis CBS 6054] | XP_001387895 |
|  | MGG_10408.6 | UR | | Y | FAD binding domain containing protein [Pyrenophora tritici-repentis Pt-1C-BFP] | XP_001934605 |
|  | MGG_14956.6 | UR | | N | cytochrome b5 reductase, putative [Aspergillus flavus NRRL3357] | EED48869 |
|  | MGG_11856.6 | UR | | N | peroxidase, putative [Talaromyces stipitatus ATCC 10500] | EED21063 |
|  | MGG_00973.6 | UR | | Y | FAD dependent oxidoreductase, putative [Aspergillus clavatus NRRL1] | XP_001272391 |
|  | MGG_00973.6 | UR | | Y | FAD dependent oxidoreductase, putative [Aspergillus clavatus NRRL1] | XP_001272391 |
|  | MGG_07949.6 | UR | | N | choline dehydrogenase [Pyrenophora tritici-repentis Pt-1C-BFP] | XP_001937164 |
|  | MGG_13573.6 | DR | | N | FAD binding oxidoreductase, putative [Aspergillus fumigatus A1163] | EDP50847 |
|  | MGG_07626.6 | DR | | Y | cytochrome P450 monooxygenase [Botryotinia fuckeliana] | CAE76652 |
|  | MGG_12003.6 | DR | | N | aldo/keto reductase, putative [Talaromyces stipitatus ATCC 10500] | EED15849 |
|  | MGG_09715.6 | DR | | N | aldo-keto reductase (AKR13), puatative [Neosartorya fischeri NRRL181] | XP_001262703 |
|  | MGG_07937.6 | DR | | N | ketopantoate reductase family protein, putative [Penicillium marneffei ATCC 18224] | XP_002152325 |
|  | MGG_00402.6 | DR | | N | short-chain dehydrogenase, putative [Penicillium marneffei ATCC 18224] | XP_002149745 |
|  | MGG_11274.6 | DR | | Y | monoxygenase [Pyrenophora tritici-repentis Pt-1C-BFP] | XP_001931985 |
|  | MGG_13464.6 | DR | | Y | laccase [Gaeumannomyces graminis var. tritici] | CAD10749 |
|  | MGG_11608.6 | DR | | Y | laccase [Gaeumannomyces graminis var. tritici] | CAD10747 |
|  | MGG_05140.6 | DR | | N | 2OG-Fe(II) oxygenase family oxidoreductase, putative [Aspergillus clavatus NRRL 1] | XP_001270320 |
|  | MGG_02329.6 | DR | | N | cytochrome P450 3A17 [Pyrenophora tritici-repentis Pt-1C-BFP] | XP_001942298 |
|  | MGG_02818.6 | DR | | Y | FAD binding domain protein [Neosartorya fischeri NRRL 181] | XP_001262117 |
|  | MGG_07629.6 | DR | | N | flavin-binding monooxygenase-like protein [Aspergillus flavus NRRL3357] | EED53649 |
|  | MGG_09188.6 | DR | | Y | malate dehydrogenase [Aspergillus fumigatus Af293] | XP_755713 |
|  | MGG_07580.6 | DR | | Y | GMC oxidoreductase [Aspergillus clavatus NRRL 1] | XP_001273036 |
|  | MGG_04684.6 | DR | | Y | cytochrome P450 phenylacetate 2-hydroxylase [Aspergillus fumigatus Af293] | XP_748171 |
|  | MGG_15026.6 | DR | | N | glucose-methanol-choline (gmc) oxidoreductase, putative[Aspergillus flavus NRRL3357] | EED49404 |
|  | MGG_05084.6 | DR | | N | NAD(P)H-dependent FMN reductase LOT6, putative [Aspergillus flavus NRRL3357] | EED54383 |
|  | MGG_10359.6 | DR | | N | GNAT family acetyltransferase, putative [Aspergillus clavatus NRRL1]. | XP_001275874 |
|  | MGG_00678.6 | DR | | N | methyltransferase (predicted) [Schizosaccharomyces pombe]. | NP_588543 |
|  | MGG_01297.6 | DR | | N | flavin-nucleotide-binding protein [Pseudovibrio sp. JE062]. | EEA96114 |
|  | MGG_04469.6 | DR | | Y | cytochrome P450 78A3 [Pyrenophora tritici-repentis Pt-1C-BFP] | XP_001936907 |
|  | MGG_01923.6 | DR | | N | putative alcohol dehydrogenase [Colletotrichum cereale] | ABR20285 |
|  | MGG_12742.6 | DR | | N | periplasmic nitrate reductase, putative [Aspergillus flavus NRRL3357] | EED55085 |
|  | MGG_10254.6 | DR | | N | taurine catabolism dioxygenase TauD [Pyrenophora tritici-repentis Pt-1C-BFP] | XP_001936704 |
|  | MGG_08519.6 | DR | | N | aldehyde reductase (GliO), putative [Neosartorya fischeri NRRL 181] | XP_001266331 |
|  | MGG_00276.6 | DR | | N | FAD binding domain protein [Neosartorya fischeri NRRL 181] | XP_001258713 |
|  | MGG_10907.6 | DR | | N | FAD-dependent oxygenase, putative [Aspergillus flavus NRRL3357] | EED53712 |
|  | MGG_06784.6 | DR | | N | aldehyde reductase I (ARI), putative [Aspergillus fumigatus A1163] | EDP52824 |
|  | MGG_07626.6 | DR | | Y | cytochrome P450 monooxygenase [Botryotinia fuckeliana] | CAE76652 |
|  | MGG_10518.6 | DR | | N | acyl-CoA dehydrogenase family protein [Aspergillus flavus NRRL3357]. | EED53053 |
|  | MGG_13764.6 | DR | | Y | RecName: Full=Bilirubin oxidase; Flags: Precursor | Q12737 |
|  | MGG_00220.6 | DR | | N | oxidoreductase, zinc-binding [Neosartorya fischeri NRRL 181] | XP_001263273 |
|  | MGG_03414.6 | DR | | N | short chain dehydrogenase/reductase family [Penicillium marneffei ATCC 18224] | XP_002147732 |
|  | MGG_09398.6 | DR | | Y | L-ascorbate oxidase [Laccaria bicolor S238N-H82] | XP_001875324 |
|  | MGG_11075.6 | DR | | Y | cytochrome P450 monooxygenase [Gibberella fujikuroi] | CAA75566 |
|  | MGG_10961.6 | DR | | Y | FAD-dependent oxidase, putative [Penicillium marneffei ATCC 18224] | XP_002145721 |
|  | MGG_09189.6 | DR | | Y | choline dehydrogenase [Pyrenophora tritici-repentis Pt-1C-BFP] | XP_001937164 |
|  | MGG_08349.6 | DR | | N | short-chain dehydrogenase, putative [Talaromyces stipitatus ATCC 10500] | EED21280 |
|  | MGG_01368.6 | DR | | N | short-chain dehydrogenases/reductase, putative [Talaromyces stipitatus ATCC 10500] | EED13656 |
|  | MGG_04751.6 | DR | | N | monooxygenase, putative [Talaromyces stipitatus ATCC 10500] | EED21311 |
|  | MGG_02792.6 | DR | | Y | related to n-alkane-inducible cytochrome P450 [Neurospora crassa] | CAC10088 |
|  | MGG_11663.6 | DR | | Y | zinc-binding oxidoreductase CipB [Pyrenophora tritici-repentis Pt-1C-BFP] | XP_001930726 |
|  | MGG_05940.6 | DR | | N | short chain dehydrogenase/reductase, putative [Neosartorya fischeri NRRL 181] | XP_001262718 |
|  | MGG_08074.6 | DR | | N | NADP-dependent glutamate dehydrogenase | Q9HGU4 |
|  | MGG_08297.6 | DR | | N | NADPH dehydrogenase [Botryotinia fuckeliana B05.10] | XP_001558622 |
|  | MGG_12742.6 | DR | | N | periplasmic nitrate reductase, putative [Aspergillus flavus NRRL3357] | EED55085 |
|  | MGG_09139.6 | DR | | Y | laccase [Gaeumannomyces graminis var. tritici] | CAD10748 |
|  | MGG_09297.6 | DR | | N | acohol dehydrogenase domain protein [Klebsiella pneumoniae 342] | YP_002239015 |
|  | MGG_01713.6 | DR | | N | norsolorinic acid reductase [Neurospora crassa OR74A] | XP_961973 |
|  | MGG_08494.6 | DR | | Y | putative cytochrome P450 [Fusarium heterosporum] | AAV66104 |
|  | MGG_11274.6 | DR | | Y | monoxygenase [Pyrenophora tritici-repentis Pt-1C-BFP] | XP_001931985 |
|  | MGG_04345.6 | DR | | N | pisatin demethylase [Pyrenophora tritici-repentis Pt-1C-BFP] | XP_001939116 |
|  | MGG_01569.6 | DR | | N | minor allergen Alt a 7 [Neurospora crassa OR74A] | XP_965630 |
|  | MGG_04404.6 | DR | | Y | pisatin demethylase [Pyrenophora tritici-repentis Pt-1C-BFP] | XP_001940087 |
| **Metabolism (GO:0008152)** | | | | | | |
|  | MGG_14590.6 | UR | | N | FAD binding domain containing protein [Pyrenophora tritici-repentis Pt-1C-BFP] | XP_001935120 |
|  | MGG_06868.6 | UR | | N | acetolactate synthase [Magnaporthe grisea] | AAB81248 |
|  | MGG_10671.6 | UR | | N | CND15p [Botryotinia fuckeliana] | AAQ16575 |
|  | MGG_07078.6 | UR | | N | DUF775 domain protein [Penicillium marneffei ATCC 18224] | XP_002145022 |
|  | MGG_04248.6 | UR | | N | oligopeptide transporter [Laccaria bicolor S238N-H82] | XP_001881083 |
|  | MGG_15248.6 | UR | | N | nonribosomal peptide synthase GliP-like, putative [Aspergillus flavus NRRL3357] | EED49635 |
|  | MGG_10671.6 | UR | | N | CND15p [Botryotinia fuckeliana] | AAQ16575 |
|  | MGG_04437.6 | UR | | N | heat shock protein 78, mitochondrial precursor [Neurospora crassaOR74A] | XP_964055 |
|  | MGG_07571.6 | DR | | Y | LysM domain protein [Neosartorya fischeri NRRL 181] | XP_00125734 |
|  | MGG_15113.6 | DR | | N | aldo/keto reductase, putative [Talaromyces stipitatus ATCC 10500] | EED22571 |
|  | MGG_12983.6 | DR | | N | short-chain dehydrogenase, putative [Talaromyces stipitatus ATCC10500] | EED21264 |
|  | MGG_10631.6 | DR | | N | glycoside hydrolase family 24 protein [Laccaria bicolor S238N-H82] | XP_001887554 |
|  | MGG_10547.6 | DR | | N | ankyrin repeat-containing protein, putative [Penicillium marneffei ATCC 18224] | XP_002151313 |
|  | MGG_09945.6 | DR | | N | cytochrome P450 46A1 [Pyrenophora tritici-repentis Pt-1C-BFP] | XP_001938675 |
|  | MGG_07883.6 | DR | | N | short-chain dehydrogenase, putative [Aspergillus flavus NRRL3357] | EED49906 |
|  | MGG_13518.6 | DR | | N | short chain dehydrogenase, putative [Penicillium marneffei ATCC 18224] | XP_002150505 |
|  | MGG_12228.6 | DR | | N | alcohol dehydrogenase, putative [Penicillium marneffei ATCC 18224] | XP_002144973 |
|  | MGG_07573.6 | DR | | N | calpain-like protein [Talaromyces stipitatus ATCC 10500] | EED22037 |
|  | MGG_10023.6 | DR | | Y | short-chain dehydrogenase, putative [Penicillium marneffei ATCC 18224] | XP_002150110 |
|  | MGG_10360.6 | DR | | N | haloalkanoic acid dehalogenase [Aspergillus fumigatus Af293] | XP_751320 |
|  | MGG_07935.6 | DR | | N | mandelate racemase/muconate lactonizing enzyme family protein[Penicillium marneffei ATCC 18224] | XP_002153081 |
|  | MGG_08989.6 | DR | | N | short chain dehydrogenase (AtsC), putative [Aspergillus flavus NRRL3357] | EED51702 |
|  | MGG_00357.6 | DR | | N | short-chain dehydrogenase/reductase family protein, putative [Talaromyces stipitatus ATCC 10500] | EED21262 |
|  | MGG_13518.6 | DR | | N | short chain dehydrogenase, putative [Penicillium marneffei ATCC 18224 | XP_002150505 |
|  | MGG_14767.6 | DR | | N | related to AM-toxin synthetase (AMT) [Neurospora crassa] | CAD70509 |
|  | MGG_03773.6 | DR | | N | dioxygenase, putative [Neosartorya fischeri NRRL 181] | XP_001265856 |
|  | MGG_08527.6 | DR | | N | nucleoside-diphosphate-sugar epimerase, putative [Neosartorya fischeri NRRL 181] | XP_001263186 |
|  | MGG_07491.6 | DR | | N | short-chain dehydrogenase, putative [Aspergillus clavatus NRRL 1] | XP_001268764 |
|  | MGG_05988.6 | DR | | N | putative phosphatidic acid phosphatase [Pleurotus sp. 'Florida'] | CAD10795 |
|  | MGG_08297.6 | DR | | N | NADPH dehydrogenase [Botryotinia fuckeliana B05.10] | XP_001558622 |
|  | MGG_05163.6 | DR | | N | Hsp70 family protein [Penicillium marneffei ATCC 18224] | XP_002147340 |
|  | MGG_07261.6 | DR | | N | 2-nitropropane dioxygenase precursor [Neurospora crassa OR74A] | XP_957588 |
|  | MGG_05759.6 | DR | | N | related to hxB protein [Neurospora crassa] | CAB97294 |
|  | MGG_10792.6 | DR | | N | salicylate hydroxylase, putative [Aspergillus clavatus NRRL 1] | XP_001273741 |
|  | MGG_13895.6 | DR | | N | fructose-bisphosphate aldolase, putative [Aspergillus flavus NRRL3357] | EED47090 |
|  | MGG_08695.6 | DR | | N | NAD-binding Rossmann fold oxidoreductase family protein [Neosartorya fischeri NRRL 181] | XP_001267589 |
|  | MGG_09836.6 | DR | | N | NAD dependent epimerase/dehydratase, putative [Aspergillus flavus NRRL3357] | EED47405 |
|  | MGG_01506.6 | DR | | N | 6-phosphogluconate dehydrogenase 2 [Pyrenophora tritici-repentis Pt-1C-BFP] | XP_001941482 |
|  | MGG_07933.6 | DR | | N | dihydrodipicolinate synthetase family protein [Aspergillus clavatus NRRL 1] | XP_001276612 |
|  | MGG_07009.6 | DR | | N | short chain dehydrogenase [Botryotinia fuckeliana B05.10] | XP_001556323 |
|  | MGG_02302.6 | DR | | N | HhH-GPD family base excision DNA repair protein [Aspergillus flavus NRRL3357] | EED51369 |
|  | MGG_06006.6 | DR | | N | lipase/serine esterase, putative [Talaromyces stipitatus ATCC 10500] | EED14390 |
|  | MGG_00953.6 | DR | | N | 2OG-Fe(II) oxygenase family oxidoreductase [Pseudomonas fluorescens Pf-5] | YP_259456 |
|  | MGG_01564.6 | DR | | N | aspartate aminotransferase, putative [Aspergillus flavus NRRL3357] | EED58109 |
|  | MGG_11468.6 | DR | | N | NmrA-like family protein [Aspergillus fumigatus Af293] | XP_731527 |
|  | MGG_01544.6 | DR | | N | cytochrome P450 monooxygenase, putative [Magnaporthe grisea 70-15] | XP_367715 |
|  | MGG_05125.6 | DR | | N | Ankyrin repeat protein [Neosartorya fischeri NRRL 181] | XP_001258750 |
|  | MGG_00385.6 | DR | | N | NRPS-like enzyme, putative [Aspergillus flavus NRRL3357] | EED52459 |
|  | MGG_10412.6 | DR | | N | allantoinase [Neurospora crassa OR74A] | XP_959940 |
|  | MGG_06917.6 | DR | | N | GTP cyclohydrolase II [Pyrenophora tritici-repentis Pt-1C-BFP] | XP_001931331 |
|  | MGG_01903.6 | DR | | N | alpha/beta hydrolase fold domain containing protein [Pyrenophora tritici-repentis Pt-1C-BFP] | XP_001942195 |
|  | MGG_10913.6 | DR | | N | short-chain dehydrogenase, putative [Talaromyces stipitatus ATCC10500] | EED21280 |
|  | MGG_03130.6 | DR | | N | dual specificity protein phosphatase PPS1 [Pyrenophora tritici-repentis Pt-1C-BFP] | XP_001939285 |
| **Response to stress (GO:0006965)** | | | | | | |
|  | MGG_10368.6 | UR | | N | cytochrome c peroxidase, mitochondrial precursor [Ajellomyces capsulatus NAm1] | XP_001536337 |
|  | MGG_12848.6 | UR | | N | RTA1 domain protein, putative [Neosartorya fischeri NRRL 181] | XP_001266412 |
|  | MGG_08980.6 | UR | | N | heat shock protein (Sti1), putative [Penicillium marneffei ATCC 18224] | XP_002146473 |
|  | MGG_09138.6 | DR | | N | glutathione S-transferase Ure2-like [Aspergillus fumigatus Af293] | XP_751380 |
| **Regulation of transcription (GO:0006355)** | | | | | | |
|  | MGG_13800.6 | UR | | N | mitochondrial exoribonuclease Cyt-4 [Aspergillus fumigatus Af293]. | XP_746777 |
|  | MGG_02436.6 | UR | | N | fungal specific transcription factor, putative [Penicillium marneffei ATCC 18224] | XP_002144445 |
|  | MGG_14358.6 | DR | | N | zinc finger protein [Aedes aegypti] | XP_001662330 |
|  | MGG_10548.6 | DR | | N | Pfs, NACHT and WD domain protein [Aspergillus fumigatus Af293] | XP_748801 |
|  | MGG_02006.6 | DR | | N | bZIP transcription factor (Atf7), putative [Aspergillus clavatus NRRL 1] | XP_001268265 |
|  | MGG_10422.6 | DR | | N | C6 transcription factor OefC [Aspergillus flavus NRRL3357]. | EED57819 |
|  | MGG_03133.6 | DR | | N | potential zinc finger transcription factor [Candida albicans SC5314] | XP_712367 |
|  | MGG_12424.6 | DR | | N | C6 transcription factor, putative [Aspergillus flavus NRRL3357] | EED48127 |
| **Transport (GO:0006810)** | | | | | | |
|  | MGG_10783.6 | UR | | N | aquaporin [Pyrenophora tritici-repentis Pt-1C-BFP] | XP_001934329 |
|  | MGG_04899.6 | UR | | N | multidrug resistance protein 2 [Pyrenophora tritici-repentis Pt-1C-BFP] | XP_001937339 |
|  | MGG_00040.6 | UR | | N | high affinity glucose transporter ght1 [Pyrenophora tritici-repentis Pt-1C-BFP] | XP_001933283 |
|  | MGG_04657.6 | UR | | N | maltose permease MAL61 [Pyrenophora tritici-repentis Pt-1C-BFP] | XP_001941424 |
|  | MGG_15435.6 | UR | | N | MFS transporter, putative [Aspergillus clavatus NRRL 1] | XP_001276296 |
|  | MGG_04216.6 | UR | | N | amino acid permease [Aspergillus fumigatus Af293] | XP_748191 |
|  | MGG_09354.6 | UR | | N | oligopeptide transporter, putative [Penicillium marneffei ATCC18224] | XP_002149499 |
|  | MGG_05803.6 | DR | | N | subtilisin, putative [Penicillium marneffei ATCC 18224] | XP_002152751 |
|  | MGG_03360.6 | DR | | N | related to carboxylic acid transport protein JEN1 [Neurospora crassa] | CAB88550 |
|  | MGG_09015.6 | DR | | N | opsin-like protein [Gibberella fujikuroi] | CAD97459 |
|  | MGG_07885.6 | DR | | N | vitamin H transporter, putative [Aspergillus flavus NRRL3357] | EED47092 |
|  | MGG_07606.6 | DR | | N | dicarboxylic amino acid permease [Aspergillus terreus NIH2624] | XP_001218343 |
|  | MGG_00156.6 | DR | | N | NmrA family transcriptional regulator, putative [Penicillium marneffei ATCC 18224] | XP_002148633 |
|  | MGG_03360.6 | DR | | N | related to carboxylic acid transport protein JEN1 [Neurospora crassa] | CAB88550 |
|  | MGG_03123.6 | DR | | N | MATE efflux family protein subfamily, putative [Aspergillus clavatus NRRL 1] | XP_001268211 |
|  | MGG_10293.6 | DR | | N | sugar transporter, putative [Aspergillus flavus NRRL3357] | EED53374 |
|  | MGG_08446.6 | DR | | N | major myo-inositol transporter iolT [Pyrenophora tritici-repentis Pt-1C-BFP] | XP_001933447 |
|  | MGG_01568.6 | DR | | N | MFS monocarboxylate transporter, putative [Aspergillus clavatus NRRL 1] | XP_001273582 |
|  | MGG_13442.6 | DR | | N | pantothenate transporter, putative [Aspergillus flavus NRRL3357] | EED48090 |
|  | MGG_01511.6 | DR | | N | a multdrug transfer [Monascus pilosus] | BAE44306 |
|  | MGG_00275.6 | DR | | N | pantothenate transporter, putative [Aspergillus flavus NRRL3357] | EED46481 |
|  | MGG_05946.6 | DR | | N | putative sugar transporter [Gibberella moniliformis] | ABV60281 |
|  | MGG_04594.6 | DR | | N | magnesium and cobalt transport protein CorA [Pelobacter carbinolicus DSM 2380] | YP_358119 |
|  | MGG_07228.6 | DR | | N | oligopeptide transporter [Laccaria bicolor S238N-H82] | XP_001887846 |
|  | MGG_05929.6 | DR | | N | MFS hexose transporter, putative [Neosartorya fischeri NRRL 181] | XP_001258822 |
|  | MGG_07546.6 | DR | | N | MFS maltose permease [Aspergillus fumigatus Af293] | XP_747524 |
|  | MGG_04852.6 | DR | | N | P-type ATPase [Schizosaccharomyces pombe]. | XP_001713045/ |
|  | MGG_05445.6 | DR | | N | H /K ATPase alpha subunit, putative [Penicillium marneffei ATCC 18224] | XP_002152781 |
|  | MGG_05526.6 | DR | | N | ammonium transporter MEP2 [Neurospora crassa OR74A] | XP_961677 |
|  | MGG_02346.6 | DR | | N | sugar transporter, putative [Aspergillus flavus NRRL3357] | EED53463 |
|  | MGG_07661.6 | DR | | N | K+ homeostasis protein Kha1 [Aspergillus fumigatus Af293] | XP_747491 |
|  | MGG_04850.6 | DR | | N | efflux pump protein [Pyrenophora tritici-repentis Pt-1C-BFP] | XP_001939435 |
|  | MGG_01417.6 | DR | | N | MFS multidrug transporter, putative [Talaromyces stipitatus ATCC10500] | EED17303 |
|  | MGG_07639.6 | DR | | N | excitatory amino acid transporter 1 [Pyrenophora tritici-repentis Pt-1C-BFP] | XP_001930712 |
|  | MGG_15060.6 | DR | | N | MFS transporter, putative [Aspergillus flavus NRRL3357] | EED47578 |
|  | MGG_08948.6 | DR | | N | florfenicol exporter, putative [Talaromyces stipitatus ATCC 10500] | EED20513 |
|  | MGG_02530.6 | DR | | N | MFS quinate transporter QutD [Talaromyces stipitatus ATCC 10500] | EED13381 |
|  | MGG_11530.6 | DR | | N | MFS transporter, putative [Talaromyces stipitatus ATCC 10500] | EED14792 |
|  | MGG_10938.6 | DR | | N | MFS transporter, putative [Neosartorya fischeri NRRL 181] | XP_001262994 |
|  | MGG_07616.6 | DR | | N | succinate/fumarate mitochondrial transporter [Neurospora crassa OR74A] | XP_962756 |
|  | MGG_11530.6 | DR | | N | MFS transporter, putative [Talaromyces stipitatus ATCC 10500] | EED14792 |
|  | MGG_04251.6 | DR | | N | sodium/phosphate symporter [Aspergillus fumigatus Af293] | XP_748875 |
|  | MGG_01778.6 | DR | | N | probable aflatoxin efflux pump AFLT [Neurospora crassa] | CAF06057 |
|  | MGG_09076.6 | DR | | N | MFS monocarboxylate transporter, putative [Neosartorya fischeri NRRL 181] | XP_001263879 |
|  | MGG_02093.6 | DR | | N | MFS multidrug transporter, putative [Aspergillus clavatus NRRL 1] | XP_001275966 |
|  | MGG_11754.6 | DR | | N | ATP-binding cassette transporter, putative [Talaromyces stipitatus ATCC 10500] | EED19536 |
|  | MGG_03957.6 | DR | | N | DUF6 domain protein [Aspergillus fumigatus Af293] | XP_752298 |
|  | MGG_01446.6 | DR | | N | sugar transporter, putative [Aspergillus flavus NRRL3357] | EED49844 |
|  | MGG_09941.6 | DR | | N | ABC transporter [Aspergillus fumigatus Af293] | XP_753691 |
|  | MGG_13334.6 | DR | | N | histidine permease [Pyrenophora tritici-repentis Pt-1C-BFP] | XP_001935450 |
|  | MGG_04864.6 | DR | | Y | allantoin permease, putative [Talaromyces stipitatus ATCC 10500] | EED14067 |
|  | MGG_10896.6 | DR | | N | MFS allantoate transporter, putative [Aspergillus flavus NRRL3357] | EED56273 |
| **Pathogenicity (GO:0009405)** | | | | | | |
|  | MGG_09875.6 | DR | | Y | CAS1 [Colletotrichum gloeosporioides] | ABK41436 |
|  | MGG_05871.6 | DR | | Y | integral membrane protein PTH11[Magnaporthe grisea] | AAD30436 |
|  | MGG_10315.6 | DR | | Y | MPG1[Magnaporthe grisea] | XP_366095.1 |
| **Others** | | | | | | |
|  | MGG_05240.6 | UR | | N | MYB DNA-binding domain containing protein [Pyrenophora tritici-repentis Pt-1C-BFP] | XP_001941024 |
|  | MGG_08104.6 | UR | | N | 40 kDa peptidyl-prolyl cis-trans isomerase [Neurospora crassa] | XP_955863 |
|  | MGG_02043.6 | UR | | N | BTB/POZ domain protein [Aspergillus clavatus NRRL 1]. | XP_001267759 |
|  | MGG_08161.6 | UR | | N | SAM domain methyltransferase, putative [Aspergillus flavus NRRL3357] | EED56621 |
|  | MGG_02308.6 | UR | | N | DUF1275 domain protein [Aspergillus clavatus NRRL 1. | XP_001273117 |
|  | MGG_03439.6 | UR | | Y | acid phosphatase [Aspergillus fumigatus Af293] | XP_746350 |
|  | MGG_07632.6 | UR | | Y | endonuclease/exonuclease/phosphatase family protein [Aspergillus fumigatus Af293] | XP_756020 |
|  | MGG_03995.6 | UR | | Y | carboxypeptidase S1, putative [Aspergillus clavatus NRRL 1] | XP_001274058 |
|  | MGG_02885.6 | UR | | N | membrane protein, putative [Rhodobacterales bacterium HTCC2654] | ZP_01015675 |
|  | MGG_05216.6 | UR | | N | TBC domain protein, putative [Talaromyces stipitatus ATCC 10500] | EED20147 |
|  | MGG_03983.6 | UR | | N | Hsp70 nucleotide exchange factor (Fes1) [Aspergillus fumigatus Af293] | XP_747603 |
|  | MGG_06460.6 | UR | | N | mitochondrial ribosomal protein subunit Mrp49 (predicted) [Schizosaccharomyces pombe]. | NP_595741 |
|  | MGG_07164.6 | UR | | N | RING finger domain protein [Talaromyces stipitatus ATCC 10500] | EED15533 |
|  | MGG_05240.6 | UR | | N | MYB DNA-binding domain containing protein [Pyrenophora tritici-repentis Pt-1C-BFP] | XP_001941024 |
|  | MGG_00811.6 | UR | | N | GTP binding protein (EngB), putative [Neosartorya fischeri NRRL 181] | XP_001258441 |
|  | MGG_07676.6 | UR | | N | chitin binding protein, putative [Aspergillus flavus NRRL3357]. | EED52495 |
|  | MGG_09467.6 | UR | | Y | integral membrane protein, putative [Neosartorya fischeri NRRL181] | XP_001264348 |
|  | MGG_02332.6 | UR | | N | necrosis-inducing protein [Streptomyces sp. Mg1] | YP_002179429 |
|  | MGG_06755.6 | UR | | N | integral membrane protein, putative [Talaromyces stipitatus ATCC 10500] | EED17823 |
|  | MGG_03706.6 | UR | | Y | integral membrane protein [Penicillium marneffei ATCC 18224] | XP_002148740 |
|  | MGG_06323.6 | UR | | N | integral membrane protein [Aspergillus clavatus NRRL 1] | XP_001276041 |
|  | MGG_01764.6 | DR | | Y | integral membrane protein [Talaromyces stipitatus ATCC 10500] | EED17189 |
|  | MGG_08535.6 | DR | | N | integral membrane protein [Aspergillus fumigatus A1163] | EDP53610 |
|  | MGG_12981.6 | DR | | N | Cupin domain protein [Aspergillus flavus NRRL3357] | EED49549 |
|  | MGG_03529.6 | DR | | Y | integral membrane protein [Aspergillus clavatus NRRL 1] | XP_001268327 |
|  | MGG_09394.6 | DR | | N | LRP16 family protein [Aspergillus fumigatus Af293] | XP_754239 |
|  | MGG_15250.6 | DR | | N | inositol monophosphatase QutG, putative [Aspergillus clavatus NRRL 1] | XP_001269115 |
|  | MGG_10738.6 | DR | | N | mitochondrial chaperone bcs1, putative [Penicillium marneffei ATCC 18224] | XP_002148435 |
|  | MGG_11305.6 | DR | | Y | YqcI [Bacillus amyloliquefaciens FZB42] | YP_001419963 |
|  | MGG_11654.6 | DR | | N | surface layer protein [Bacillus cereus] | YP_001966612 |
|  | MGG_05384.6 | DR | | N | Ankyrin repeat protein [Neosartorya fischeri NRRL 181] | XP_001258750 |
|  | MGG_02559.6 | DR | | N | MOSC domain protein [Aspergillus fumigatus Af293] | XP_755117 |
|  | MGG_05861.6 | DR | | N | GNAT family acetyltransferase, putative [Neosartorya fischeri NRRL 181] | XP_001261802 |
|  | MGG_02239.6 | DR | | Y | phosphoserine aminotransferase [alpha proteobacterium BAL199]. | ZP_02191003 |
|  | MGG_11047.6 | DR | | N | DNA repair protein rad5 [Pyrenophora tritici-repentis Pt-1C-BFP] | XP_001936125 |
|  | MGG_10098.6 | DR | | N | cell wall anchored protein, putative [Aspergillus clavatus NRRL 1] | XP_001268879 |
|  | MGG_13913.6 | DR | | Y | tetraspanin Tsp3 [Sclerotinia sclerotiorum] | ABX46545 |
|  | MGG_10236.6 | DR | | N | kinesin light chain [Pyrenophora tritici-repentis Pt-1C-BFP] | XP_001942318 |
|  | MGG_05017.6 | DR | | N | glutamyl-tRNA(Gln) amidotransferase, subunit A [Penicillium marneffei ATCC 18224] | XP_002145146 |
|  | MGG_10480.6 | DR | | N | ankyrin repeat protein [Aspergillus fumigatus Af293] | XP_752820 |
|  | MGG_12480.6 | DR | | N | aromatic prenyl transferase [Neotyphodium lolii] | ABF20224 |
|  | MGG_08495.6 | DR | | N | tol related protein [Magnaporthe grisea] | BAD67182 |
|  | MGG_09404.6 | DR | | Y | feruloyl esterase B precursor, putative [Aspergillus flavus NRRL3357] | EED47503 |
|  | MGG_11084.6 | DR | | N | TPR domain protein [Aspergillus fumigatus A1163] | EDP52007 |
|  | MGG_06326.6 | DR | | N | vacuolar ATP synthase 16 kDa proteolipid subunit [Sclerotinia sclerotiorum 1980] | XP_001588693 |
|  | MGG_03364.6 | DR | | N | secreted protein [Streptomyces sviceus ATCC 29083] | YP_002206261 |
|  | MGG_03501.6 | DR | | N | DUF1264 domain protein [Penicillium marneffei ATCC 18224] | XP_002145074 |
|  | MGG_02648.6 | DR | | N | dynamin family GTPase, putative [Aspergillus fumigatus A1163] | EDP55311 |
|  | MGG_07870.6 | DR | | N | solid-state culture specific ATP-grasp domain protein [Aspergillus flavus NRRL3357] | EED54474 |
|  | MGG_01920.6 | DR | | N | C2H2 type zinc finger domain protein [Neosartorya fischeri NRRL 181] | XP_001259773 |
|  | MGG_03826.6 | DR | | Y | kelch repeat-containing protein [Methylobacterium extorquens PA1] | YP_001638060 |
|  | MGG_00715.6 | DR | | N | glucose-repressible gene protein [Botryotinia fuckeliana B05.10] | XP_001549859 |
|  | MGG_04209.6 | DR | | Y | related to exo-alpha-sialidase / neuraminidase [Neurospora crassa] | CAD70852 |
|  | MGG_09648.6 | DR | | Y | ribonuclease R [Parabacteroides distasonis ATCC 8503] | YP_001304670 |
|  | MGG_15319.6 | DR | | N | zinc finger protein, putative [Talaromyces stipitatus ATCC 10500] | EED21400 |
|  | MGG_07794.6 | DR | | N | ankyrin repeat and SAM domain containing protein 6 [Pyrenophora tritici-repentis Pt-1C-BFP] | XP_001935595 |
|  | MGG_07346.6 | DR | | N | methyltransferase [Aspergillus fumigatus Af293]. | XP_754096 |
|  | MGG_05632.6 | DR | | Y | siderophore biosynthesis enzyme, putative [Talaromyces stipitatus ATCC 10500]. | EED19426 |
|  | MGG_10764.6 | DR | | N | promyelocytic leukemia zinc finger protein [Bos taurus]. | ABH06317 |
|  | MGG_09360.6 | DR | | Y | endonuclease/exonuclease/phosphatase family protein [Talaromyces stipitatus ATCC 10500]. | EED12861 |
|  | MGG_02114.6 | DR | | N | interferon-induced GTP-binding protein Mx2 [Pyrenophora tritici-repentis Pt-1C-BFP]. | XP_001942266 |
|  | MGG_10237.6 | DR | | Y | accumulation-associated protein [Staphylococcus epidermidis RP62A]. | CAB77251 |
|  | MGG_02647.6 | DR | | Y | UVI-1 [Bipolaris oryzae] | BAA96293 |
