# Supplementary material for: Two Phosphodiesterase Genes, PDEL and PDEH, Regulate Development and Pathogenicity by Modulating Intracellular Cyclic AMP Levels in Magnaporthe oryzae
Source: PLoS One. 2011 Feb 28;6(2):e17241. doi: 10.1371/journal.pone.0017241 (PMC3046207; doi:10.1371/journal.pone.0017241)
Supplement: Table S5 — Categorization of genes only dependent on PDEL , PDEH and PDEL & PDEH , respectively. (DOC) [file pone.0017241.s007.doc]

| **Table S5. Categorization of genes only dependent on *PDEL*, *PDEH* and *PDEL & PDEH*, respectively.** | | | | | | |
| --- | --- | --- | --- | --- | --- | --- |
| **Category** | **Gene ID** | **Exp.** | **Signal P** | | **Blast hit** | **NCBI_ID** |
| ***PDEL*-dependent** | | | | | | |
|  | MGG_03082.6 | UR | | N | Heterokaryon incompatibility protein (HET)[Neurospora crassa] | CAD70324 |
|  | MGG_05767.6 | UR | | N | Phosphorylase superfamily[Aspergillus flavus NRRL3357] | EED46522 |
|  | MGG_04076.6 | UR | | N | low-density lipoprotein receptor YWTD repeat [Burkholderia sp. H160] | ZP_03266143 |
|  | MGG_10751.6 | UR | | N | peroxisomal copper amine oxidase [Neurospora crassa OR74A] | XP_960480 |
|  | MGG_04406.6 | UR | | N | NACHT and Ankyrin domain protein [Aspergillus fumigatus Af293] | XP_747379 |
|  | MGG_04407.6 | UR | | N | cation diffusion facilitator 10 [Pyrenophora tritici-repentis Pt-1C-BFP] | XP_001930927 |
|  | MGG_09072.6 | UR | | N | alcohol oxidase p68 [Cochliobolus victoriae] | AAK14990 |
|  | MGG_05798.6 | UR | | Y | cutinase precursor [Pyrenophora tritici-repentis Pt-1C-BFP] | XP_001935104 |
|  | MGG_05912.6 | UR | | N | amidohydrolase, putative [Penicillium marneffei ATCC 18224] | XP_002149455 |
|  | MGG_11209.6 | UR | | N | ABC transporter, putative [Aspergillus clavatus NRRL 1] | XP_001268636 |
|  | MGG_02072.6 | UR | | N | Amino-acid permease inda1[Trichoderma atroviride] | P34054 |
|  | MGG_15023.6 | UR | | N | C6 finger domain protein, putative [Talaromyces stipitatus ATCC10500] | EED21828 |
|  | MGG_11860.6 | UR | | N | NACHT and WD domain protein [Aspergillus fumigatus Af293] | XP_754863 |
|  | MGG_13669.6 | UR | | N | MFS peptide transporter, putative [Aspergillus flavus NRRL3357] | EED49889 |
|  | MGG_13261.6 | UR | | N | serine-threonine rich protein, putative [Talaromyces stipitatus ATCC 10500] | EED23712 |
|  | MGG_09165.6 | UR | | N | sialidase [Trichophyton equinum] | ACJ04076 |
|  | MGG_10005.6 | UR | | N | glycerol kinase, putative [Talaromyces stipitatus ATCC 10500] | EED22001 |
|  | MGG_07666.6 | UR | | Y | class I alpha-mannosidase [Ophiostoma novo-ulmi] | AAG48158 |
|  | MGG_11611.6 | UR | | N | amino acid transporter, putative [Penicillium marneffei ATCC18224] | XP_002153282 |
|  | MGG_03349.6 | UR | | N | Auxin Efflux Carrier superfamily [Aspergillus clavatus NRRL 1] | XP_001276302 |
|  | MGG_05946.6 | UR | | N | putative sugar transporter [Gibberella moniliformis] | ABV60281 |
|  | MGG_00671.6 | UR | | Y | arginase family protein [Aspergillus clavatus NRRL 1] | XP_001273079 |
|  | MGG_10005.6 | UR | | N | glycerol kinase, putative [Talaromyces stipitatus ATCC 10500] | EED22001 |
|  | MGG_02114.6 | UR | | N | interferon-induced GTP-binding protein Mx2 [Pyrenophora tritici-repentis Pt-1C-BFP] | XP_001942266 |
|  | MGG_02269.6 | UR | | N | LPS glycosyltransferase [Aspergillus fumigatus Af293] | XP_747140 |
|  | MGG_10498.6 | UR | | Y | endopeptidase [Rhodococcus jostii RHA1] | YP_703158 |
|  | MGG_07787.6 | UR | | N | regulatory P domain-containing protein [Hahella chejuensis KCTC 2396] | YP_433708 |
|  | MGG_05165.6 | UR | | Y | actin filament organization protein App1-like [Aspergillus fumigatus Af293] | XP_750414 |
|  | MGG_09806.6 | UR | | Y | alpha-1,3-mannosyltransferase, putative [Aspergillus fumigatusA1163] | EDP55339 |
|  | MGG_00931.6 | UR | | N | alpha/beta hydrolase fold [Ochrobactrum anthropi ATCC 49188] | YP_001369382 |
|  | MGG_12612.6 | UR | | N | MFS multidrug transporter, putative [Talaromyces stipitatus ATCC10500] | EED14464 |
|  | MGG_03287.6 | UR | | N | related to alpha-amylase [Neurospora crassa] | CAE75731 |
|  | MGG_12291.6 | UR | | Y | beta-hexosaminidase, putative [Aspergillus flavus NRRL3357] | EED53191 |
|  | MGG_10497.6 | UR | | N | bli-3 protein [Pyrenophora tritici-repentis Pt-1C-BFP] | XP_001941956 |
|  | MGG_04344.6 | UR | | N | regulatory P domain-containing protein [Hahella chejuensis KCTC2396] | YP_433708 |
|  | MGG_10046.6 | UR | | N | pantothenate transporter, putative [Talaromyces stipitatus ATCC10500] | EED19237 |
|  | MGG_10407.6 | DR | | N | integral membrane protein (Pth11), putative [Aspergillus flavus NRRL3357] | EED45803 |
|  | MGG_03995.6 | DR | | N | carboxypeptidase S1, putative [Aspergillus clavatus NRRL 1] | XP_001274058 |
|  | MGG_03806.6 | DR | | Y | calmodulin-related protein, putative [Arabidopsis thaliana] | NP_179170 |
|  | MGG_05854.6 | DR | | N | cytochrome P450 monooxygenase, putative [Aspergillus clavatus NRRL 1] | XP_001271830 |
|  | MGG_13598.6 | DR | | Y | endothiapepsin precursor [Neurospora crassa OR74A] | XP_963600 |
|  | MGG_03828.6 | DR | | N | cytochrome P450, putative [Neosartorya fischeri NRRL 181] | XP_001266395 |
|  | MGG_07587.6 | DR | | N | isoflavone reductase family protein [Aspergillus clavatus NRRL1] | XP_001274110 |
|  | MGG_08416.6 | DR | | N | triacylglycerol lipase, putative [Aspergillus flavus NRRL3357] | EED45307 |
|  | MGG_14061.6 | DR | | Y | oxalate decarboxylase, putative [Aspergillus flavus NRRL3357] | EED56627 |
|  | MGG_10083.6 | DR | | N | endoglucanase 3 precursor [Neurospora crassa OR74A] | XP_964159 |
|  | MGG_02243.6 | DR | | N | amidohydrolase family protein [Aspergillus fumigatus A1163] | EDP53371 |
|  | MGG_08003.6 | DR | | N | UbiA prenyltransferase [Chloroflexus sp. Y-400-fl] | YP_002568665 |
|  | MGG_13798.6 | DR | | N | putative amino acid permease [Glomus mosseae] | AAX81451 |
|  | MGG_05457.6 | DR | | N | taurine catabolism dioxygenase TauD, TfdA family protein[Neosartorya fischeri NRRL 181] | XP_001266665 |
|  | MGG_07946.6 | DR | | N | integral membrane protein, putative [Talaromyces stipitatus ATCC10500] | EED17823 |
|  | MGG_03828.6 | DR | | N | cytochrome P450, putative [Neosartorya fischeri NRRL 181]. | XP_001266395 |
| ***PDEH*-dependent** | | | | | | |
|  | MGG_07881.6 | UR | | N | GA15419 [Drosophila pseudoobscura pseudoobscura]. | XP_001359800 |
|  | MGG_03508.6 | UR | | Y | Glycosyl hydrolase family 3 N terminal domain Cel3e [Hypocrea jecorina]. | AAP57760 |
| ***PDEL & PDEH*-dependent** | | | | | | |
|  | MGG_02332.6 | UR | | N | necrosis-inducing protein [Streptomyces sp. Mg1] | YP_002179429 |
|  | MGG_05240.6 | UR | | N | MYB DNA-binding domain containing protein [Pyrenophora tritici-repentis Pt-1C-BFP] | XP_001941024 |
|  | MGG_00246.6 | UR | | N | NADP-dependent alcohol dehydrogenase C [Pyrenophora tritici-repentis Pt-1C-BFP] | XP_001941135 |
|  | MGG_14061.6 | UR | | Y | oxalate decarboxylase, putative [Aspergillus flavus NRRL3357] | EED56627 |
|  | MGG_06167.6 | UR | | Y | phytase [Aspergillus flavus NRRL3357] | EED53727 |
|  | MGG_04349.6 | UR | | N | cytochrome P450, putative [Penicillium marneffei ATCC 18224] | XP_002149366 |
|  | MGG_14590.6 | UR | | N | FAD binding domain containing protein [Pyrenophora tritici-repentis Pt-1C-BFP] | XP_001935120 |
|  | MGG_06759.6 | UR | | Y | heat shock protein 90 [Humicola fuscoatra] | ACF93232 |
|  | MGG_03439.6 | UR | | Y | acid phosphatase [Aspergillus fumigatus Af293] | XP_746350 |
|  | MGG_00811.6 | UR | | N | GTP binding protein (EngB), putative [Neosartorya fischeri NRRL 181] | XP_001258441 |
|  | MGG_08944.6 | UR | | Y | 1,4-alpha-glucan branching enzyme [Shewanella amazonensis SB2B] | YP_928327 |
|  | MGG_06868.6 | UR | | N | acetolactate synthase [Magnaporthe grisea] | AAB81248 |
|  | MGG_06868.6 | UR | | N | acetolactate synthase [Magnaporthe grisea] | AAB81248 |
|  | MGG_06069.6 | UR | | N | endoglucanase, putative [Aspergillus clavatus NRRL 1] | XP_001275694 |
|  | MGG_04899.6 | UR | | N | multidrug resistance protein 2 [Pyrenophora tritici-repentis Pt-1C-BFP] | XP_001937339 |
|  | MGG_04191.6 | UR | | N | heat shock 70 kDa protein, mitochondrial precursor [Neurospora crassa OR74A] | XP_961753 |
|  | MGG_09527.6 | UR | | Y | endoglucanase, putative [Talaromyces stipitatus ATCC 10500]. | EED18911 |
|  | MGG_03995.6 | UR | | Y | carboxypeptidase S1, putative [Aspergillus clavatus NRRL 1] | XP_001274058 |
|  | MGG_03056.6 | UR | | Y | aorsin endoprotease precursor [Laccaria bicolor S238N-H82] | XP_001876156 |
|  | MGG_04216.6 | UR | | N | amino acid permease [Aspergillus fumigatus Af293] | XP_748191 |
|  | MGG_03144.6 | UR | | N | Ulp1 protease family protein [Talaromyces stipitatus ATCC 10500] | EED13023 |
|  | MGG_10408.6 | UR | | Y | FAD binding domain containing protein [Pyrenophora tritici-repentis Pt-1C-BFP] | XP_001934605 |
|  | MGG_06755.6 | UR | | N | integral membrane protein, putative [Talaromyces stipitatus ATCC 10500] | EED17823 |
|  | MGG_08980.6 | UR | | N | heat shock protein (Sti1), putative [Penicillium marneffei ATCC 18224] | XP_002146473 |
|  | MGG_08066.6 | UR | | N | fungal cellulose binding domain containing protein [Pyrenophora tritici-repentis Pt-1C-BFP] | XP_001937817 |
|  | MGG_12848.6 | UR | | N | RTA1 domain protein, putative [Neosartorya fischeri NRRL 181] | XP_001266412 |
|  | MGG_08809.6 | UR | | N | YMR098Cp-like protein [Saccharomyces cerevisiae AWRI1631] | EDZ70131 |
|  | MGG_13800.6 | UR | | N | mitochondrial exoribonuclease Cyt-4 [Aspergillus fumigatus Af293]. | XP_746777 |
|  | MGG_07164.6 | UR | | N | RING finger domain protein [Talaromyces stipitatus ATCC 10500] | EED15533 |
|  | MGG_02043.6 | UR | | N | BTB/POZ domain protein [Aspergillus clavatus NRRL 1]. | XP_001267759 |
|  | MGG_11856.6 | UR | | N | peroxidase, putative [Talaromyces stipitatus ATCC 10500] | EED21063 |
|  | MGG_07868.6 | DR | | N | endo-1,4-beta-xylanase precursor [Aspergillus terreus NIH2624] | XP_001212588 |
|  | MGG_07884.6 | DR | | N | YALI0F01628p [Yarrowia lipolytica] | XP_504869 |
|  | MGG_03880.6 | DR | | N | alcohol dehydrogenase 1 [Pyrenophora tritici-repentis Pt-1C-BFP] | XP_001939279 |
|  | MGG_00220.6 | DR | | N | oxidoreductase, zinc-binding [Neosartorya fischeri NRRL 181] | XP_001263273 |
|  | MGG_08535.6 | DR | | N | integral membrane protein [Aspergillus fumigatus A1163] | EDP53610 |
|  | MGG_09138.6 | DR | | N | glutathione S-transferase Ure2-like [Aspergillus fumigatus Af293] | XP_751380 |
|  | MGG_10023.6 | DR | | Y | short-chain dehydrogenase, putative [Penicillium marneffei ATCC 18224] | XP_002150110 |
|  | MGG_13765.6 | DR | | Y | extracelular serine carboxypeptidase, putative [Talaromyces stipitatus ATCC 10500] | EED18491 |
|  | MGG_10360.6 | DR | | N | haloalkanoic acid dehalogenase [Aspergillus fumigatus Af293] | XP_751320 |
|  | MGG_09601.6 | DR | | Y | glycosyl hydrolase, putative [Aspergillus flavus NRRL3357] | EED57325 |
|  | MGG_07935.6 | DR | | N | mandelate racemase/muconate lactonizing enzyme family protein[Penicillium marneffei ATCC 18224] | XP_002153081 |
|  | MGG_00357.6 | DR | | N | short-chain dehydrogenase/reductase family protein, putative [Talaromyces stipitatus ATCC 10500] | EED21262 |
|  | MGG_00715.6 | DR | | N | glucose-repressible gene protein [Botryotinia fuckeliana B05.10] | XP_001549859 |
|  | MGG_01368.6 | DR | | N | short-chain dehydrogenases/reductase, putative [Talaromyces stipitatus ATCC 10500] | EED13656 |
|  | MGG_07219.6 | DR | | N | polyketide synthase [Ophiostoma piceae] | ABD47522 |
|  | MGG_03773.6 | DR | | N | dioxygenase, putative [Neosartorya fischeri NRRL 181] | XP_001265856 |
|  | MGG_12424.6 | DR | | N | C6 transcription factor, putative [Aspergillus flavus NRRL3357] | EED48127 |
|  | MGG_03957.6 | DR | | N | DUF6 domain protein [Aspergillus fumigatus Af293] | XP_752298 |
|  | MGG_05940.6 | DR | | N | short chain dehydrogenase/reductase, putative [Neosartorya fischeri NRRL 181] | XP_001262718 |
|  | MGG_08527.6 | DR | | N | nucleoside-diphosphate-sugar epimerase, putative [Neosartorya fischeri NRRL 181] | XP_001263186 |
|  | MGG_12742.6 | DR | | N | periplasmic nitrate reductase, putative [Aspergillus flavus NRRL3357] | EED55085 |
|  | MGG_09139.6 | DR | | Y | laccase [Gaeumannomyces graminis var. tritici] | CAD10748 |
|  | MGG_13334.6 | DR | | N | histidine permease [Pyrenophora tritici-repentis Pt-1C-BFP] | XP_001935450 |
|  | MGG_07794.6 | DR | | N | ankyrin repeat and SAM domain containing protein 6 [Pyrenophora tritici-repentis Pt-1C-BFP] | XP_001935595 |
|  | MGG_09445.6 | DR | | N | similar to synaptotagmin, putative [Tribolium castaneum] | XP_974305 |
|  | MGG_01446.6 | DR | | N | sugar transporter, putative [Aspergillus flavus NRRL3357] | EED49844 |
|  | MGG_08446.6 | DR | | N | major myo-inositol transporter iolT [Pyrenophora tritici-repentis Pt-1C-BFP] | XP_001933447 |
|  | MGG_07877.6 | DR | | Y | secreted dipeptidyl peptidase [Neosartorya fischeri NRRL 181] | XP_001260402 |
|  | MGG_05128.6 | DR | | N | amino acid transporter (predicted) [Schizosaccharomyces pombe 972h-] | NP_588250 |
|  | MGG_05163.6 | DR | | N | Hsp70 family protein [Penicillium marneffei ATCC 18224] | XP_002147340 |
|  | MGG_07346.6 | DR | | N | methyltransferase [Aspergillus fumigatus Af293]. | XP_754096 |
|  | MGG_01568.6 | DR | | N | MFS monocarboxylate transporter, putative [Aspergillus clavatus NRRL 1] | XP_001273582 |
|  | MGG_13442.6 | DR | | N | pantothenate transporter, putative [Aspergillus flavus NRRL3357] | EED48090 |
|  | MGG_15060.6 | DR | | N | MFS transporter, putative [Aspergillus flavus NRRL3357] | EED47578 |
|  | MGG_11274.6 | DR | | Y | monoxygenase [Pyrenophora tritici-repentis Pt-1C-BFP] | XP_001931985 |
|  | MGG_08774.6 | DR | | Y | vi polysaccharide biosynthesis protein vipA/tviB [Pyrenophora tritici-repentis Pt-1C-BFP] | XP_001933886 |
|  | MGG_10896.6 | DR | | N | MFS allantoate transporter, putative [Aspergillus flavus NRRL3357] | EED56273 |
|  | MGG_05946.6 | DR | | N | putative sugar transporter [Gibberella moniliformis] | ABV60281 |
|  | MGG_07261.6 | DR | | N | 2-nitropropane dioxygenase precursor [Neurospora crassa OR74A] | XP_957588 |
|  | MGG_09433.6 | DR | | N | glucanase, putative [Aspergillus clavatus NRRL 1] | XP_001273400 |
|  | MGG_06917.6 | DR | | N | GTP cyclohydrolase II [Pyrenophora tritici-repentis Pt-1C-BFP] | XP_001931331 |
|  | MGG_10533.6 | DR | | Y | agmatinase [Aspergillus fumigatus Af293] | XP_753336 |
|  | MGG_09404.6 | DR | | Y | feruloyl esterase B precursor, putative [Aspergillus flavus NRRL3357] | EED47503 |
|  | MGG_09076.6 | DR | | N | MFS monocarboxylate transporter, putative [Neosartorya fischeri NRRL 181] | XP_001263879 |
|  | MGG_15319.6 | DR | | N | zinc finger protein, putative [Talaromyces stipitatus ATCC 10500] | EED21400 |
|  | MGG_01922.6 | DR | | N | polysaccharide deacetylase family protein [Pyrenophora tritici-repentis Pt-1C-BFP] | XP_001932774 |
|  | MGG_10299.6 | DR | | N | L-lysine 2,3-aminomutase, putative [Aspergillus flavus NRRL3357] | EED47871 |
|  | MGG_03130.6 | DR | | N | dual specificity protein phosphatase PPS1 [Pyrenophora tritici-repentis Pt-1C-BFP] | XP_001939285 |
|  | MGG_12742.6 | DR | | N | periplasmic nitrate reductase, putative [Aspergillus flavus NRRL3357] | EED55085 |
|  | MGG_04594.6 | DR | | N | magnesium and cobalt transport protein CorA [Pelobacter carbinolicus DSM 2380] | YP_358119 |
|  | MGG_06917.6 | DR | | N | GTP cyclohydrolase II [Pyrenophora tritici-repentis Pt-1C-BFP] | XP_001931331 |
|  | MGG_00194.6 | DR | | Y | protein rds1 [Neurospora crassa OR74A] | XP_956641 |
|  | MGG_08695.6 | DR | | N | NAD-binding Rossmann fold oxidoreductase family protein [Neosartorya fischeri NRRL 181] | XP_001267589 |
|  | MGG_00678.6 | DR | | N | methyltransferase (predicted) [Schizosaccharomyces pombe]. | NP_588543 |
|  | MGG_00050.6 | DR | | N | alpha-L-fucosidase 2 precursor, putative [Aspergillus flavus NRRL3357] | EED47314 |
|  | MGG_04684.6 | DR | | Y | cytochrome P450 phenylacetate 2-hydroxylase [Aspergillus fumigatus Af293] | XP_748171 |
|  | MGG_06738.6 | DR | | N | G-protein coupled receptor [Trichoderma atroviride] | ABD46750 |
|  | MGG_07616.6 | DR | | N | succinate/fumarate mitochondrial transporter [Neurospora crassa OR74A] | XP_962756 |
|  | MGG_10518.6 | DR | | N | acyl-CoA dehydrogenase family protein [Aspergillus flavus NRRL3357]. | EED53053 |
|  | MGG_07580.6 | DR | | Y | GMC oxidoreductase [Aspergillus clavatus NRRL 1] | XP_001273036 |
|  | MGG_01506.6 | DR | | N | 6-phosphogluconate dehydrogenase 2 [Pyrenophora tritici-repentis Pt-1C-BFP] | XP_001941482 |
|  | MGG_01094.6 | DR | | N | C2 domain protein [Penicillium marneffei ATCC 18224] | XP_002151241 |
|  | MGG_10938.6 | DR | | N | MFS transporter, putative [Neosartorya fischeri NRRL 181] | XP_001262994 |
|  | MGG_05929.6 | DR | | N | MFS hexose transporter, putative [Neosartorya fischeri NRRL 181] | XP_001258822 |
|  | MGG_07005.6 | DR | | Y | CFEM domain protein [Aspergillus clavatus NRRL 1] | XP_001268442 |
|  | MGG_03529.6 | DR | | Y | integral membrane protein [Aspergillus clavatus NRRL 1] | XP_001268327 |
|  | MGG_05599.6 | DR | | Y | glycosyl hydrolase, putative [Neosartorya fischeri NRRL 181] | XP_001264012 |
|  | MGG_01564.6 | DR | | N | aspartate aminotransferase, putative [Aspergillus flavus NRRL3357] | EED58109 |
|  | MGG_07629.6 | DR | | N | flavin-binding monooxygenase-like protein [Aspergillus flavus NRRL3357] | EED53649 |
|  | MGG_05140.6 | DR | | N | 2OG-Fe(II) oxygenase family oxidoreductase, putative [Aspergillus clavatus NRRL 1] | XP_001270320 |
|  | MGG_02114.6 | DR | | N | interferon-induced GTP-binding protein Mx2 [Pyrenophora tritici-repentis Pt-1C-BFP]. | XP_001942266 |
|  | MGG_15452.6 | DR | | N | glycosyl hydrolase family 88, putative [Penicillium marneffei ATCC 18224] | XP_002143913 |
|  | MGG_04173.6 | DR | | N | EF-hand calcium-binding domain protein, putative [Aspergillus flavus NRRL3357]. | EED51602 |
|  | MGG_09664.6 | DR | | N | beta-mannosidase [Emericella nidulans] | ABF50864 |
|  | MGG_07933.6 | DR | | N | dihydrodipicolinate synthetase family protein [Aspergillus clavatus NRRL 1] | XP_001276612 |
|  | MGG_02812.6 | DR | | N | 1-aminocyclopropane-1-carboxylate deaminase, putative [Talaromyces stipitatus ATCC 10500] | EED13104 |
|  | MGG_13913.6 | DR | | Y | tetraspanin Tsp3 [Sclerotinia sclerotiorum] | ABX46545 |
|  | MGG_05445.6 | DR | | N | H /K ATPase alpha subunit, putative [Penicillium marneffei ATCC 18224] | XP_002152781 |
|  | MGG_11047.6 | DR | | N | DNA repair protein rad5 [Pyrenophora tritici-repentis Pt-1C-BFP] | XP_001936125 |
|  | MGG_02006.6 | DR | | N | bZIP transcription factor (Atf7), putative [Aspergillus clavatus NRRL 1] | XP_001268265 |
|  | MGG_02239.6 | DR | | Y | phosphoserine aminotransferase [alpha proteobacterium BAL199]. | ZP_02191003 |
|  | MGG_05861.6 | DR | | N | GNAT family acetyltransferase, putative [Neosartorya fischeri NRRL 181] | XP_001261802 |
|  | MGG_02302.6 | DR | | N | HhH-GPD family base excision DNA repair protein [Aspergillus flavus NRRL3357] | EED51369 |
|  | MGG_10548.6 | DR | | N | Pfs, NACHT and WD domain protein [Aspergillus fumigatus Af293] | XP_748801 |
|  | MGG_08758.6 | DR | | Y | leupeptin-inactivating enzyme 1 precursor [Pyrenophora tritici-repentis Pt-1C-BFP] | NP_588250 |
|  | MGG_02559.6 | DR | | N | MOSC domain protein [Aspergillus fumigatus Af293] | XP_755117 |
|  | MGG_02530.6 | DR | | N | MFS quinate transporter QutD [Talaromyces stipitatus ATCC 10500] | EED13381 |
|  | MGG_10189.6 | DR | | Y | beta-glucosidase [Neurospora crassa OR74A] | XP_956183 |
|  | MGG_05526.6 | DR | | N | ammonium transporter MEP2 [Neurospora crassa OR74A] | XP_961677 |
|  | MGG_02817.6 | DR | | N | glutamate decarboxylase [Neurospora crassa OR74A] | XP_965818 |
|  | MGG_05024.6 | DR | | N | N-carbamoyl-L-amino acid hydrolase [Pyrenophora tritici-repentis Pt-1C-BFP] | XP_001935695 |
|  | MGG_11654.6 | DR | | N | surface layer protein [Bacillus cereus] | YP_001966612 |
|  | MGG_07937.6 | DR | | N | ketopantoate reductase family protein, putative [Penicillium marneffei ATCC 18224] | XP_002152325 |
|  | MGG_10738.6 | DR | | N | mitochondrial chaperone bcs1, putative [Penicillium marneffei ATCC 18224] | XP_002148435 |
|  | MGG_03492.6 | DR | | N | acetamidase [Coccidioides immitis RS] | XP_001248603 |
|  | MGG_09715.6 | DR | | N | aldo-keto reductase (AKR13), puatative [Neosartorya fischeri NRRL181] | XP_001262703 |
|  | MGG_08948.6 | DR | | N | florfenicol exporter, putative [Talaromyces stipitatus ATCC 10500] | EED20513 |
|  | MGG_15250.6 | DR | | N | inositol monophosphatase QutG, putative [Aspergillus clavatus NRRL 1] | XP_001269115 |
|  | MGG_02275.6 | DR | | Y | serine peptidase, putative [Aspergillus clavatus NRRL 1] | XP_001273182 |
|  | MGG_09394.6 | DR | | N | LRP16 family protein [Aspergillus fumigatus Af293] | XP_754239 |
